# Synergistic inhibition of *Aspergillus flavus* by organic acid salts: growth, oxidative stress, and aflatoxin gene modulation

**DOI:** 10.3389/fvets.2025.1608792

**Published:** 2025-12-04

**Authors:** Yingying Yu, Fengming Li, Zhiqiang Cheng, Mengfei Li, Zihao Xu, Long Sun, Changjiang Zang, Xiaobin Li, Kailun Yang

**Affiliations:** College of Animal Science, Xinjiang Agricultural University, Urumqi, China

**Keywords:** *Aspergillus flavus*, aflatoxin, composite organic acid salt, cell integrity, antioxidant indicators, *aflR*, *aflS*

## Abstract

Fungal contamination represents a critical challenge in feed production. To address this issue, organic acids and their salts are widely employed as mould-inhibiting additives. Compared to traditional organic acids, their salt derivatives offer superior stability and lower corrosivity. This study conducted an *in vitro* investigation to evaluate the effects of three organic acid salts, when used in combination, on the mycelial growth, cellular integrity, antioxidant status, and toxigenic gene expression of *Aspergillus flavus* (*A. flavus*). The results demonstrated that a composite organic acid salt of sodium diacetate, sodium dehydroacetate, and sodium benzoate (2:1:1 ratio) exerted optimal antifungal efficacy. Treatment with this composite organic acid salt effectively inhibited mycelial growth and resulted in a substantial decrease in mycelial dry weight. It also compromised cellular integrity, evidenced by a concentration-dependent increase in extracellular relative conductivity and a concurrent reduction in pH. Furthermore, the composite organic acid salt potently suppressed the biosynthesis of total lipids and trehalose but had no significant effect on the ergosterol mass fraction. The composite organic acid salt also induced oxidative stress, characterized by an increase in superoxide dismutase and glutathione peroxidase activities, suppression of catalase activity, and a significant accumulation of malondialdehyde. Compared to the control group, the MIC group of the composite organic acid salt significantly reduced the relative expression levels of genes encoding the aflatoxin synthesis pathway, including *aflR*, *aflS*, *aflC*, *aflD*, *aflT*, and *aflM* genes. In conclusion, the composite organic acid salt exhibits a multi-target, synergistic mechanism against *A. flavus* by inhibiting mycelial growth, disrupting cellular structure, inducing oxidative damage, and suppressing toxigenic gene expression. Its effectiveness, particularly at the MIC, highlights its considerable potential as a feed preservative.

## 1 Introduction

*Aspergillus flavus* (*A. flavus*), a saprophytic fungus, is characterized by its yellow to yellow-green conidial spores and septate hyphae (1, 2). It is capable of rapid growth on substrates such as animal feed and various crops. During its growth, it produces aflatoxins, which are carcinogenic, teratogenic, genotoxic, and immunotoxic (3). Aflatoxicosis caused by this fungus can result in liver damage, immunosuppression, and, in severe cases, high mortality (4, 5). Globally, 60–80% of crops are contaminated with mycotoxins (6), resulting in significant economic losses and raising concerns about food and feed safety (7). Consequently, controlling *A. flavus* contamination and developing effective antifungal strategies are critical to ensuring feed safety. Currently, the primary methods for controlling aflatoxins include physical, chemical, and biological approaches (8). Although physical methods (such as microwaves, radiation, ultraviolet light, and pulse light technology) can effectively degrade toxins, they have limitations, such as high energy consumption and potential impacts on the nutritional composition of feed (9, 10). Biological methods (such as *Trichoderma* spp., bacteria, and yeast) suppress *A. flavus* through biological competition or antagonism. However, their effectiveness is influenced by environmental conditions, and they tend to have a longer action period (11, 12).

In contrast, organic acids and their salt compounds within chemical methods demonstrate significant advantages due to their high efficiency and cost-effectiveness (13). Organic acids are classified as generally recognized as safe (GRAS) substances within allowable concentrations (14–16) and exhibit broad-spectrum antifungal activity, with minimal sensory impact on the product. Moon et al. (17) reported that the growth of *A. flavus* was completely inhibited by 0.05% benzoic acid, 0.5% acetic acid, butyric acid, and propionic acid. They do not cause noticeable changes in odor or taste, are highly stable (18), cost-effective, easy to use, and suitable for long-term preservation. Organic acid salts also possess antifungal properties; however, they offer superior solubility and stability, are gentler on feed, and enhance palatability (19). Studies have shown that sodium diacetate effectively inhibits harmful microorganisms in feed, reducing nutrient loss (20). Calcium formate has been shown to decrease the number of *Escherichia coli* in the intestines of piglets (21), and citrate salts also significantly inhibit *Clostridium perfringens* (22).

The antimicrobial mechanism of organic acids and their salts primarily involves lowering the environmental pH, which facilitates the entry of undissociated acid molecules into microbial cells, thereby disrupting the cell membrane structure (23). Furthermore, through synergistic interactions among multiple components, composite organic acids and their salts can simultaneously target various microorganisms, enhancing their antimicrobial efficacy (24, 25). Studies have shown that composite organic acids and their salts not only effectively inhibit pathogenic microorganisms but also promote the growth of beneficial microbes (26). For example, adding 0.2% composite organic acids to the diet of lactating sows resulted in a 2.0% reduction in *Escherichia coli* and a 2.2% increase in lactic acid bacteria (27); the combination of potassium sorbate and sodium benzoate was found to promote the proliferation of pediococci in maize silage (28).

The cellular structure and stress response mechanisms of *A. flavus* are key targets for revealing antifungal effects. Ergosterol, a critical component of the fungal cell membrane, regulates membrane fluidity, and total lipids and trehalose are crucial for maintaining membrane stability (29). A decrease in the levels of these three substances typically indicates irreversible damage to the cell membrane (30). On the other hand, oxidative stress response is a crucial mechanism by which fungi cope with environmental stress. The activities of superoxide dismutase (SOD), catalase (CAT), and glutathione peroxidase (GSH-Px) are key enzymes responsible for eliminating reactive oxygen species (31). In contrast, the malondialdehyde (MDA) level serves as a standard biomarker for lipid peroxidation (32). At the molecular level, the biosynthesis of aflatoxins is regulated by several key genes (33). Among them, *aflR* and *aflS* are core regulatory genes that encode transcriptional regulatory proteins, which jointly activate the expression of multiple biosynthetic genes (34). Structural genes, including *aflC* (encoding polyketide synthase), *aflD* (encoding norsolorinic acid ketoreductase), *aflM* (encoding ketoreductase), and *aflT* (encoding fungal transporter), directly participate in the biochemical reactions and export processes involved in toxin synthesis (35, 36). The expression of these genes directly influences aflatoxin levels and thus can serve as molecular indicators for evaluating the effectiveness of antimicrobial agents.

Although individual organic acids have been confirmed to possess inhibitory potential against *A. flavus*, and organic acid salts offer superior application properties. Most existing studies remain focused on single components. The combined application of multiple organic acid salts and their synergistic inhibitory mechanisms against *A. flavus* have yet to be thoroughly investigated. Therefore, this study selected three organic acid salts—sodium diacetate, sodium dehydroacetate, and sodium benzoate—to explore their individual and combined effects on the inhibition of *A. flavus*. Their effects on mycelial growth, cellular integrity, the oxidative stress response, and the expression of key toxin synthesis genes were systematically evaluated. This study aims to provide a theoretical basis and practical strategies for the safe control of *A. flavus* in animal feed.

## 2 Materials and methods

### 2.1 Microorganisms

*A. flavus* was isolated from corn samples. Potato dextrose agar (PDA) was utilized for subcultures (Qingdao Hi-tech Industrial Park Hope Bio-technology Co., Ltd., Qingdao, China), while potato dextrose broth (PDB) served as the liquid culture medium (Qingdao Hi-tech Industrial Park Hope Bio-technology Co., Ltd., Qingdao, China).

### 2.2 Test material

Sodium diacetate (CAS: 126–96-5), sodium dehydroacetate (CAS: 64039–28-7), and potassium sorbate (CAS: 24634–61-5) were purchased from Shanghai Yuanye Biotechnology Co., Ltd. (Shanghai, China); sodium benzoate (CAS: 532–32-1) and sodium butyrate (CAS: 156–54-7) were obtained from Guangdong Wengjiang Chemical Reagent Co., Ltd. (Shaoguan, China). All compounds were ≥98% pure.

### 2.3 Antifungal activity assay

The method was adapted from Zhao et al. (37) and performed in a 96-well plate using a twofold dilution approach. A sodium diacetate solution (256 mg/mL, in sterile water) and a spore suspension of *A. flavus* (10^7^ spores/mL) were prepared. The procedure was as follows:

(1) 50 μL of sterilized PDB was added to wells 1–7 50 μL of sodium diacetate was added to well 1 and serially diluted (50 μL per well) across the next five wells. After mixing in the fifth well, 50 μL was discarded. 100 μL of spore suspension was added to wells 1–5, achieving final sodium diacetate concentrations of 128, 64, 32, 16, and 8 mg/mL. (2) Wells without sodium diacetate served as positive controls, and wells without spore suspension served as negative controls. (3) The plate was incubated at 28 ± 2 °C for 72 h. Absorbance at 620 nm was measured every 24 h using an Infinite M200 microplate reader (Dicken (Shanghai) Trading Co., Ltd., Shanghai, China). The minimum inhibitory concentration (MIC) was defined as the lowest concentration at which the absorbance at 620 nm was comparable to that of the negative control well (without spores), indicating complete inhibition of fungal growth. (4) The final concentrations of sodium benzoate, potassium sorbate, and sodium butyrate were set equivalent to that of sodium diacetate. The final concentrations of sodium dehydroacetate were prepared in a gradient of 32, 16, 8, 4, 2, and 1 mg/mL.

### 2.4 Determination of the combined antifungal effect

**Figure 1:**
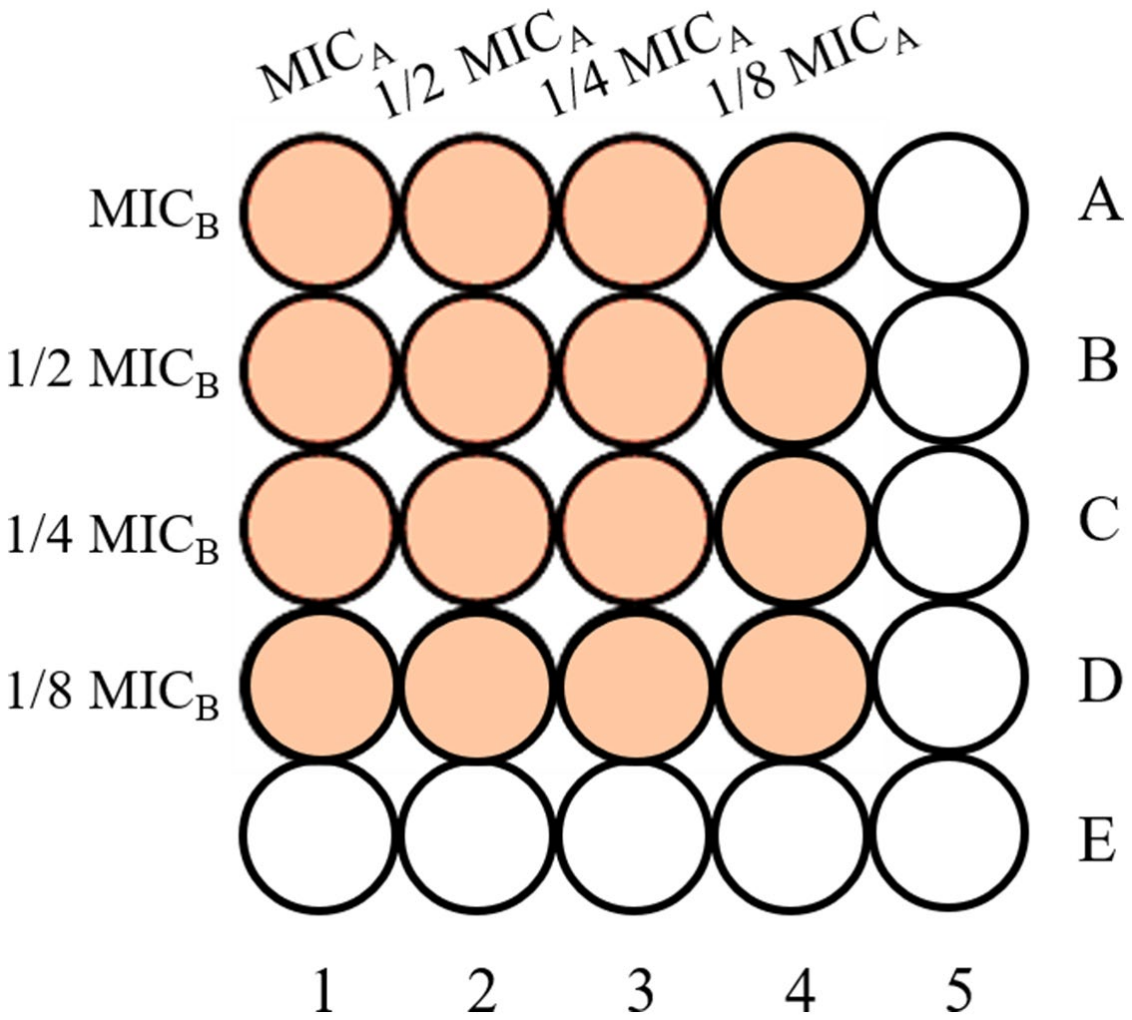
Schematic representation of the checkerboard dilution method.

The combined antimicrobial effects of two organic acid salts were assessed using the checkerboard dilution method (Figure 1) (38). Four concentration gradients were tested for each salt (1, 1/2, 1/4, and 1/8 of the MIC). In the 96-well plate, columns 1–4 contained the first salt, columns A–D the second salt, and columns five and E served as controls. After adding 50 μL of the corresponding solution to each well, 100 μL of spore suspension was added. The plate was incubated at 28 °C for 72 h, and absorbance at 620 nm was measured to determine the combined MIC. The synergistic effect was evaluated using the Fractional Inhibitory Concentration Index (FICI) (39, 40)—a key metric for quantifying the interaction strength between two antimicrobial agents. The calculated FICI clearly determines whether the combination of two organic acid salts is synergistic, additive, indifferent, or antagonistic (41). Based on the lowest FICI, the optimal salt combination was selected, and a third organic acid salt was tested in combination. The fixed ratio of this combination was used in subsequent tests, with the FICI calculated to evaluate interaction and determine the ideal ratio. The formula for calculating the FICI is shown in Equation 1.


(1) $$\text{FICI}=\frac{\mathrm{MI}C_{\mathrm{ab}}}{\mathrm{MI}C_{a}}+\frac{\mathrm{MI}C_{\mathrm{ba}}}{\mathrm{MI}C_{b}}$$

In the formula, *MIC_ab_* and *MIC_ba_* represent the MICs of the two organic acid salts when used in combination. In comparison, *MIC_a_* and *MIC_b_* represent the MICs of the two organic acid salts when used individually.

The interpretive criteria for FICI were as follows: FICI ≤ 0.5 indicates synergistic effect, 0.5 < FICI ≤ 1 indicates an additive effect, 1 < FICI ≤ 2 suggests no interaction, FICI > 2 indicates an antagonistic effect (42).

### 2.5 Determination of *A. flavus* mycelial growth

The method by Li Q et al. (43) was slightly modified to evaluate the impact of organic acid salts on *A. flavus* growth inhibition by measuring changes in mycelial biomass. The procedure was as follows:

(1) A 100 μL spore suspension (10^7^ spores/mL) was inoculated into 20 mL PDB medium and incubated at 28 ± 2 °C with shaking at 150 rpm for 24 h. (2) The culture was filtered through sterile filter paper, rinsed with sterile water, and transferred to fresh filter paper. Samples were dried at 80 °C for 24 h, cooled, and weighed to determine the dry weight of the control group. (3) For other groups, composite and individual organic acid salts were added, and incubation continued for another 24 h. The same procedure was followed, and mycelial dry weight was recorded for each group.

### 2.6 Determination of *A. flavus* cell integrity

#### 2.6.1 Determination of relative conductivity and pH values

The method of Gao et al. (44) was slightly modified. Three 6-mm *A. flavus* mycelial plugs were inoculated into 100 mL PDB and incubated at 28 °C with shaking at 150 rpm for 24 h. Different concentrations of composite organic acid salts were added, followed by an additional 24 h of incubation. The culture was filtered through gauze, washed three times with sterile water, and 0.5 g of mycelium was weighed. After adding 20 mL of sterile water, the conductivity was measured immediately. Extracellular conductivity was recorded at 0, 10, 20, 40, 80, and 120 min, then the samples were boiled for 5 min. After cooling to room temperature, the final conductivity was measured. Relative conductivity was calculated as shown in Equation 2.


(2) $$\text{Relative conductivity}\;(\%)=\frac{C_{1}-C_{W1}}{C_{2}-C_{W2}}\times 100\%$$

Where *C*_1_ and *C*_2_ represent the sample conductivities before and after boiling, respectively, *C*_*w*1_ and *C*_*w*2_ represent the conductivities of purified water before and after boiling, respectively. The pH values were measured immediately following the conductivity measurements at the aforementioned time points.

#### 2.6.2 Determination of total lipid content, ergosterol mass fraction, and trehalose content

Three 6-mm *A. flavus* mycelial plugs (cultured for 3 d) were inoculated into 75 mL of PDB and incubated at 28 °C and 150 rpm for 24 h. Composite and individual organic acid salts were added to achieve final concentrations of 1/4 MIC, 1/2 MIC, and MIC, followed by another 24 h incubation. Mycelia were harvested, filtered, washed with sterile water, and ground in liquid nitrogen to obtain mycelial powder.

Total lipid content was determined using a modified phosphovanillin method (45). Mycelial powder (10 mg) was dissolved in 1 mL sterile water, followed by 1 mL methanol-chloroform mixture (1:1) for extraction. After vigorous shaking, the mixture was centrifuged at 3,000 rpm for 10 min. The lower phase, containing lipids, was mixed with 0.2 mL saline and centrifuged again. Then, 200 μL of the lower phase was collected, and 0.2 mL of chloroform and 0.5 mL of concentrated H_2_SO_4_ were added. The mixture was heated at 100 °C for 10 min, followed by the addition of 3 mL phosphovanillin reagent. After 10 min, absorbance was measured at 520 nm. Cholesterol was used to construct the calibration curve.

The method of Li Q et al. (43) was followed. A 10 mg sample of mycelial powder was mixed with 5 mL of 25% KOH-ethanol solution, shaken for 2 min, and heated in an 85 °C water bath for 2 h. Afterwards, 1 mL of sterile water and 3 mL of n-heptane were added for extraction, and the mixture was vortexed for 3 min and left to stand for 1 h at room temperature. The upper liquid layer was collected and stored at −20 °C for 24 h. Absorbance was measured at 230 nm and 282 nm using a UV spectrophotometer. The ergosterol mass fraction was calculated as shown in Equation 3.


(3) $$\text{Ergosterol mass fraction}(\%)=\frac{\frac{A_{282}}{290}-\frac{A_{230}}{518}}{W}\times 100\%$$

In the formula, W represents the mass of mycelium in grams (g); 290 denotes the extinction coefficient of ergosterol; and 518 represents the extinction coefficient of dehydroergosterol.

Mycelia were collected, and trehalose content was measured using a trehalose assay kit (Suzhou Grace Biotechnology Co., Ltd., Suzhou, China). The anthrone colorimetric method was used to measure absorbance at 620 nm (46), and trehalose content was calculated based on the standard curve.

### 2.7 Determination of antioxidant indicators in *A. flavus*

Catalase (CAT), glutathione peroxidase (GSH-Px), and superoxide dismutase (SOD) activities, along with malondialdehyde (MDA) content, were determined using commercial assay kits purchased from Suzhou Grace Biotechnology Co., Ltd. (Suzhou, China), following the manufacturer’s instructions. According to the procedure described in Section 2.6.2, fungal mycelia were collected after being washed three times with sterile water, followed by centrifugation to discard the supernatant. A measured amount of the mycelia was then mixed with the designated extraction buffer and subjected to ultrasonic disruption in an ice bath. The resulting homogenate was centrifuged at 12,000 rpm for 10 min at 4 °C, and the supernatant was collected for biochemical analyses.

### 2.8 Determination of key gene expression levels during toxin synthesis

Total RNA was extracted from treated samples using RNA extraction and reverse transcription kits (Jiangsu Kangwei Century Biotechnology Co., Ltd., Taizhou, China) and reverse transcribed into cDNA. Quantification was performed with an RT-qPCR kit (Qiagen Bioengineering, Shenzhen, China) on a CFX96 Touch real-time PCR system (Bio-Rad Laboratories, Shanghai, China). Amplification conditions were: 95 °C for 3 min, 95 °C for 5 s, 60 °C for 30 s, with 40 cycles, followed by a melting curve analysis. The *β-tubulin* gene was used as the internal reference for data normalization. Primer sequences are listed in Table 1 (synthesized by Shanghai Bioengineering Co., Ltd., Shanghai, China). Gene expression was quantified using the 2^-ΔΔCt^ method (47).

**Table 1:** Primer sequences for RT-qPCR.

Genes	Primer sequence	Fragment lengths (bp)
*β-Tubulin*	F: CCGCTTTCTGGCAAACCATC	111
	R: TGGCCTCGTTGAAGTAGACG	
*aflR*	F: AACAAGAGGGCTACCGATGC	168
	R: ACTGTTGGTTTCTCCACCCG	
*aflS*	F: GCACTCTGGCGGGTATTCAG	128
	R: GAGCCAACTGTCGGACCAAG	
*aflC*	F: GGCCATGCTAAGGGACAGTT	120
	R: TGCTTCGTTCAGCACCAGAT	
*aflD*	F: GGTGGTTTCAACATTCCTTGAGT	82
	R: AGCCTCTCTTGACCGTGATG	
*aflT*	F: GCCCGAAAAGCAAGGAAGG	188
	R: AGTCTGGTGTTGTTTTGCGG	
*aflM*	F: CCGATGAGCAGGTAGACGAG	122
	R: TCCACTTACCCATTCGGCTG	

### 2.9 Statistical analysis

A two-factor factorial design was employed for this study. Factor A represented the type of organic acid salt (sodium diacetate, sodium dehydroacetate, sodium benzoate, or the composite organic acid salt), while Factor B corresponded to the treatment concentration (1/4 MIC, 1/2 MIC, and MIC). All experiments were performed in at least triplicate (*n* = 3). Data were presented as the mean ± standard deviation (SD). Statistical analyses were conducted using SPSS software (version 27.0; IBM Corp., New York, USA). A General Linear Model (GLM) was utilized to assess the main and interactive effects of organic acid salt type and concentration on the cellular integrity and oxidative stress indicators of *A. flavus*. A one-way analysis of variance (ANOVA) was used to compare the effects of the different organic acid salt treatments on mycelial growth and the relative expression of key genes. Post-hoc multiple comparisons were performed using Tukey’s Honestly Significant Difference (HSD) test. A significant level of *p* < 0.05 was established for all analyses. All figures were generated using Origin 2024 (OriginLab Corp., Massachusetts, USA). Detailed results, including 95% confidence intervals and information on outlier detection and data normalization, are provided in the Supplementary materials.

## 3 Results and analysis

### 3.1 MICs of the five organic acid salts

**Figure 2:**
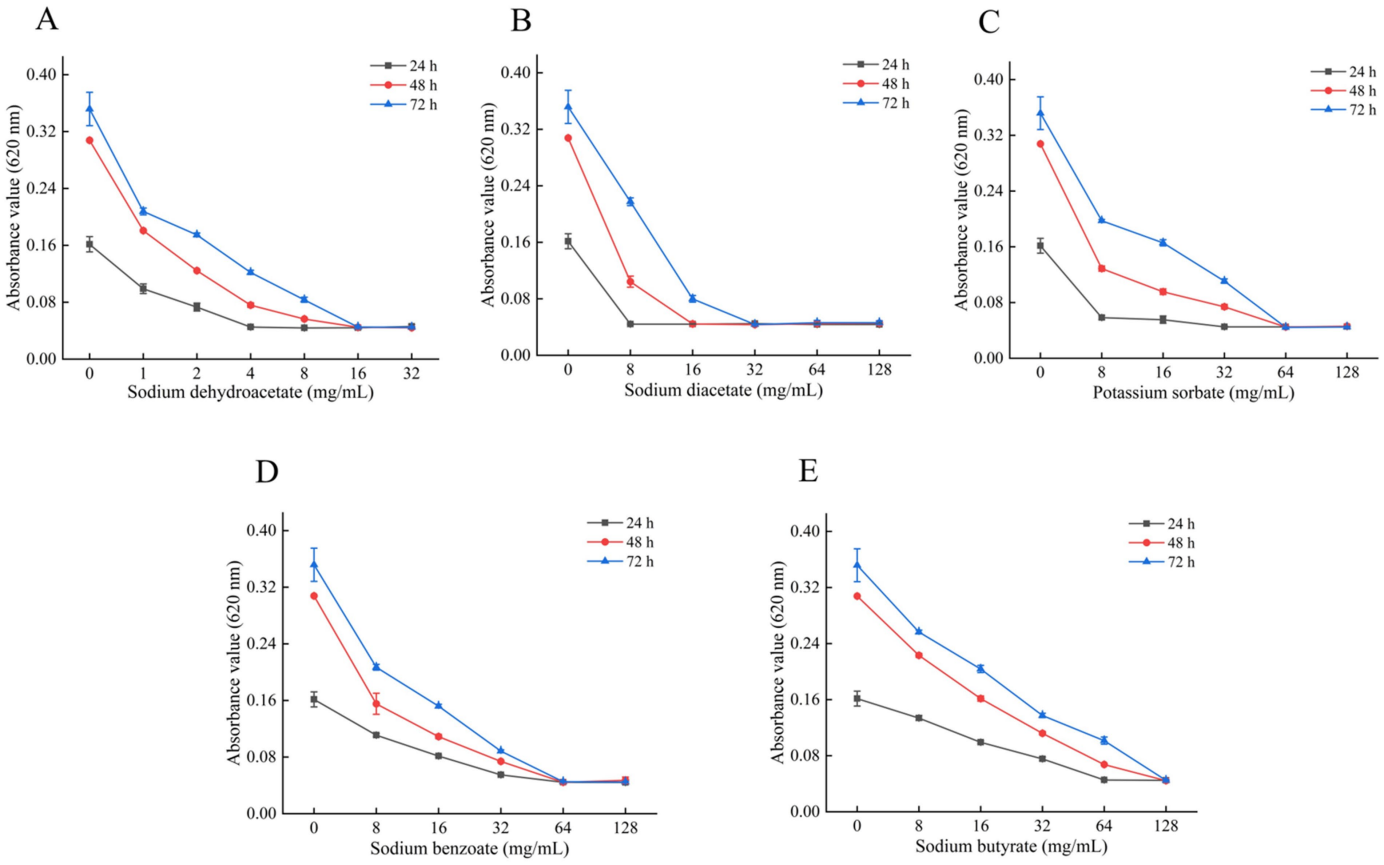
Inhibitory effects of five organic acid salts on the growth of *A. flavus.* **(A)** Sodium dehydroacetate. **(B)** Sodium diacetate. **(C)** Potassium sorbate. **(D)** Sodium benzoate. **(E)** Sodium butyrate. The minimum inhibitory concentration (MIC) was defined as the lowest concentration at which the absorbance at 620 nm was comparable to that of the negative control well (without spores), indicating complete inhibition of fungal growth. The results are expressed as Mean ± Standard Deviation. The MICs of the Sodium dehydroacetate, Sodium diacetate, Potassium sorbate, Sodium benzoate, and Sodium butyrate were 16, 32, 64, 64, and 128 mg/mL, respectively.

Inhibitory effects of five organic acid salts on the growth of *A. flavus* are shown in Figure 2. As the concentrations increased, absorbance values of *A. flavus* spores decreased, indicating inhibition of spore germination and growth in a dose-dependent manner. No absorbance increase was observed after 72 h in cultures treated with 16 mg/mL sodium dehydroacetate, 32 mg/mL sodium diacetate, 64 mg/mL potassium sorbate and sodium benzoate, and 128 mg/mL sodium butyrate, indicating complete growth inhibition at these concentrations. The MICs of the salts against *A. flavus* were 16, 32, 64, 64, and 128 mg/mL, respectively.

### 3.2 Combined antifungal effect

Table 2 details the inhibitory effects of five organic acid salt combinations. Sodium diacetate exhibited significant synergy, particularly with sodium dehydroacetate (1:1) and sodium benzoate (1:1), yielding a FICI of 0.38 for both. A stronger synergy was observed with potassium sorbate (4:1), resulting in a FICI of 0.28, while its combination with sodium butyrate (1:2) showed an additive effect (FICI = 0.75). Other combinations had FICI ≥ 2.0, indicating indifference or antagonism. The most effective synergy occurred with a 2:1:1 ratio of sodium diacetate, sodium dehydroacetate, and sodium benzoate, yielding a FICI of 0.28. The total MIC for this composite was 8 mg/mL, with sodium dehydroacetate and sodium benzoate concentrations reduced by half and fourfold, respectively, compared to their individual MICs. These results show that the composite organic acid salts have superior antimicrobial activity, with the 2:1:1 combination being the most effective.

**Table 2:** Antifungal effects of the five organic acid salt combinations against *A. flavus.*

Inhibitor concentration(mg/mL)					Total MIC	FICI	Antifungal effect
Sodium diacetate	Sodium dehydroacetate	Potassium sorbate	Sodium benzoate	Sodium benzoate			
4	4	-	-	-	8	0.38	Synergistic effect
8	-	2	-	-	10	0.28	Synergistic effect
8	-	-	8	-	16	0.38	Synergistic effect
16	-	-	-	32	48	0.75	Additive effect
-	16	64	-	-	80	2.00	No interaction effect
-	16	-	64	-	80	2.00	No interaction effect
-	16	-	-	128	144	>2.00	Antagonistic effect
-	-	64	64	-	128	>2.00	Antagonistic effect
-	-	64	-	128	192	2.00	No interaction effect
-	-	-	64	128	192	2.00	No interaction effect
4	2	-	2	-	8	0.28	Synergistic effect

FICI, fractional inhibitory concentration index. FICI ≤ 0.5 indicates synergistic effect, 0.5 < FICI ≤ 1 indicates an additive effect, 1 < FICI ≤ 2 suggests no interaction, FICI > 2 indicates an antagonistic effect.

### 3.3 Effects of different organic acid salts on mycelial dry weight

**Figure 3:**
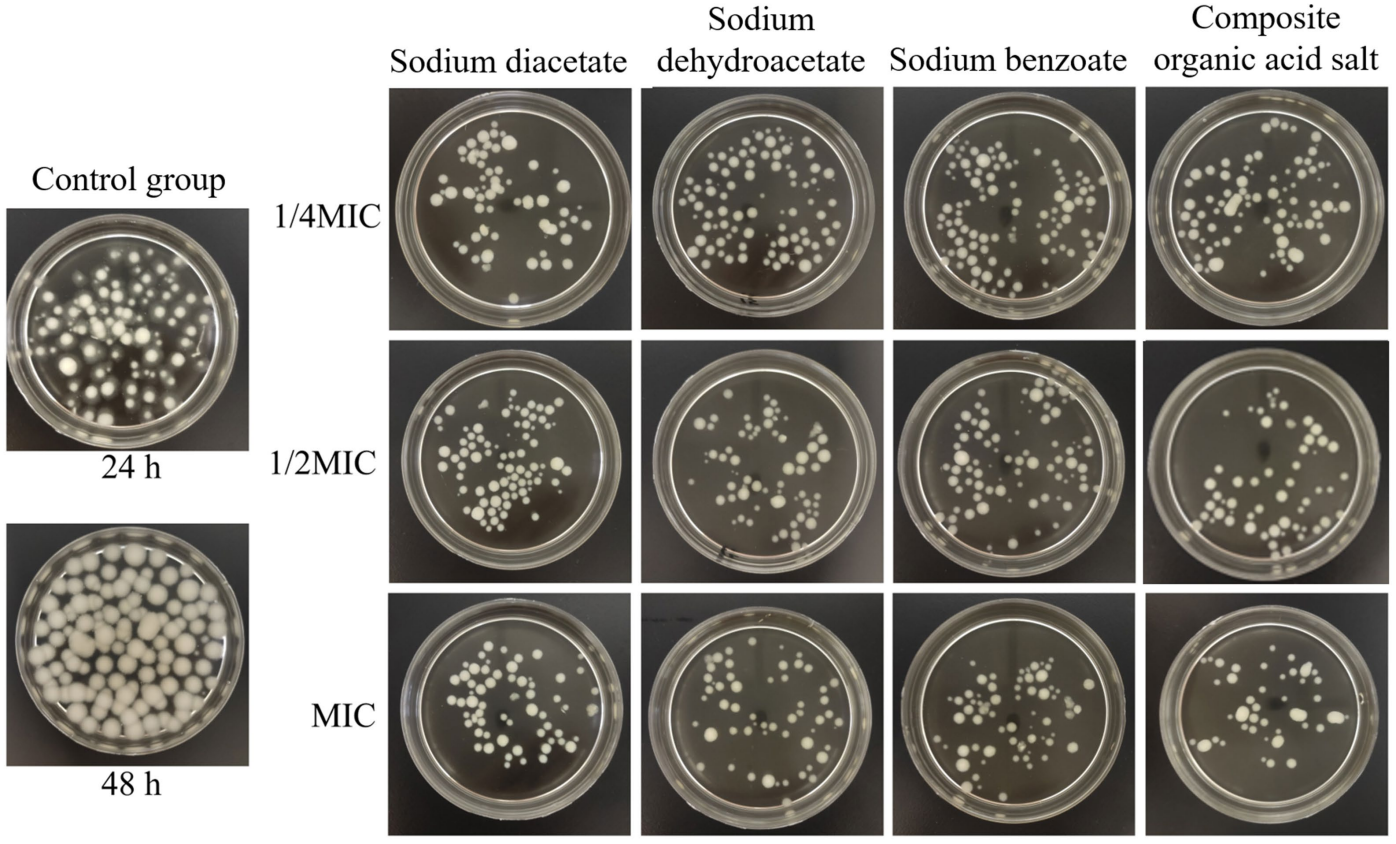
Effects of different treatments on mycelial growth. MIC, minimum inhibitory concentration.

The impact of incorporating different organic acid salts on mycelial growth is shown in Figure 3. After 48 h of incubation, the control group exhibited vigorous *A. flavus* mycelial growth, forming large, distinct spherical colonies. In contrast, the treated groups showed a marked reduction in colony size, and the number of mycelial aggregates progressively decreased with increasing concentrations of the inhibitors.

**Figure 4:**
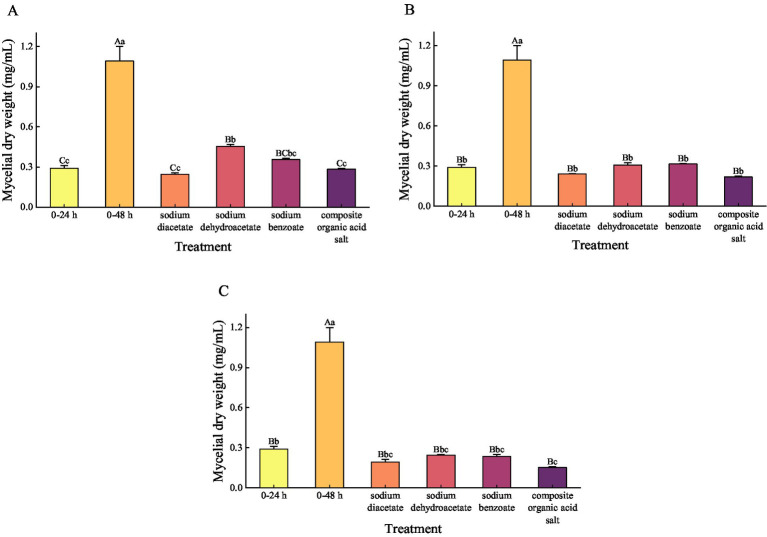
The mycelial dry weight under different treatments at a concentration of 1/4 MIC **(A)**, 1/2 MIC **(B)**, and MIC **(C)**. MIC, minimum inhibitory concentration. Mycelial dry weight was analyzed using one-way ANOVA, and results are expressed as Mean ± Standard Deviation, with multiple comparisons performed using Tukey’s test. *p* < 0.05 is significant. At the same concentration, different lowercase letters indicate a significant difference (*p* < 0.05), while different uppercase letters indicate a highly significant difference (*p* < 0.01) between treatments.

Quantitative analysis of mycelial dry weight (Figure 4) demonstrated that all organic acid salt treatments significantly inhibited the growth of *A. flavus*, with biomass levels markedly lower than those of the 48-h control group, showing a reduction ranging from 58.41 to 86.09% (*p* < 0.001). At 1/4 MIC, the dry weight of mycelia treated with sodium diacetate and the composite organic acid salts was significantly lower than that treated with sodium dehydroacetate, with reductions of 45.96% (*p* = 0.001) and 37.50% (*p* = 0.007), respectively. At 1/2 MIC and MIC concentrations, the mycelial dry weights of all treatment groups were further reduced compared to 1/4 MIC; however, no statistically significant differences were observed among the treatments at these higher concentrations (*p* > 0.05). Overall, the composite organic acid salts treatment at MIC exhibited the most pronounced inhibitory effect, reducing mycelial dry weight by 86.09% relative to the control. These results indicate that the composite treatment effectively suppresses *A. flavus* growth through both morphological disruption and biomass reduction.

### 3.4 The effects of different treatments on cell integrity

#### 3.4.1 Extracellular relative conductivity and pH

**Figure 5:**
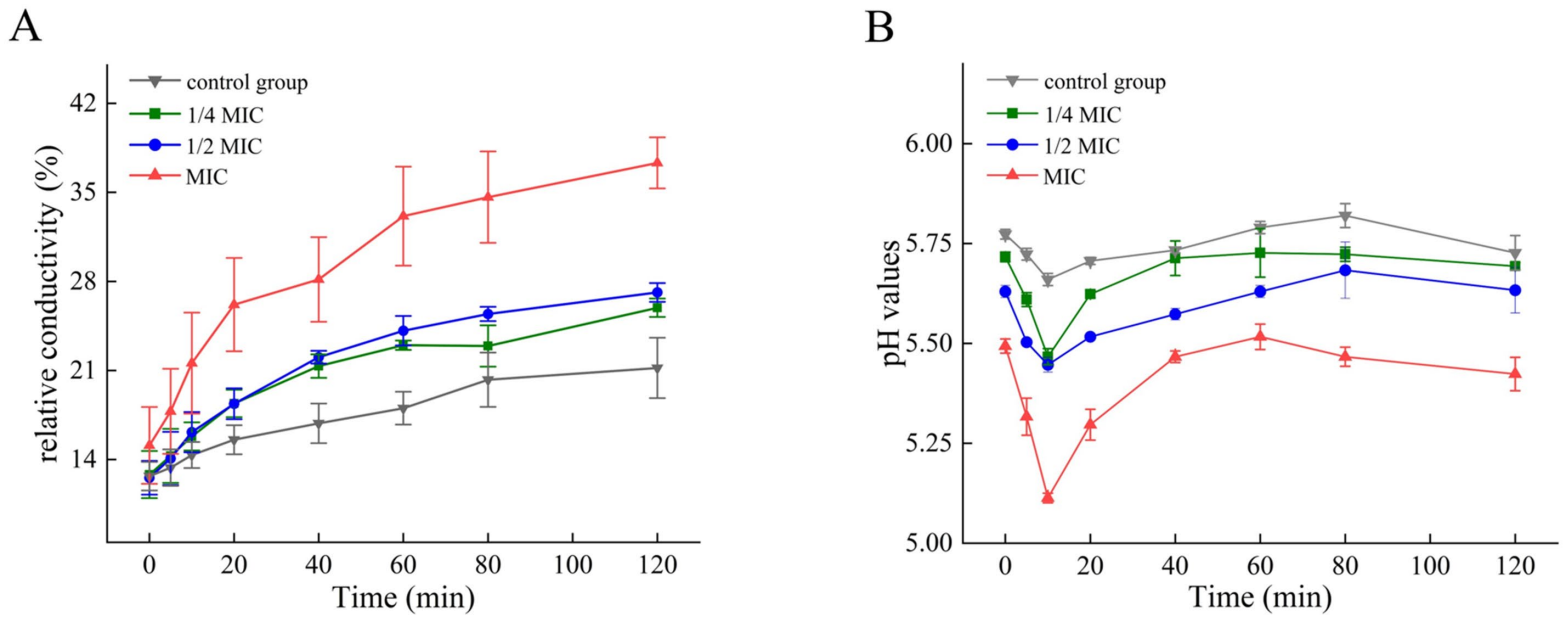
Effects of different treatments on relative conductivity **(A)** and pH values **(B)**. MIC, minimum inhibitory concentration. The relative conductivity and pH values are expressed as Mean ± standard deviation.

Figure 5 demonstrates the significant impact of composite organic acid salts on *A. flavus* cell membrane permeability and extracellular microenvironment. Extracellular relative conductivity (Figure 5A) increased over time in all groups, with the most significant rise observed at MIC, showing a dose-dependent effect. Monitoring of extracellular pH (Figure 5B) revealed that, throughout the incubation period, all treatment groups maintained lower pH values than the control, with further pH reduction observed as the concentration of composite organic acid salts increased. In all groups, pH initially decreased during the first 10 min, followed by a gradual rise. Notably, the initial pH drop was more pronounced in the treatment groups and became progressively greater with higher concentrations of the composite organic acid salts. These results indicate that the composite organic acid salts increase cell membrane permeability and acidify the extracellular environment of *A. flavus*, with both effects positively correlated with salt concentration.

#### 3.4.2 Total lipid, ergosterol and trehalose

As shown in Table 3, the concentration of the organic acid salts, the type of salt, and their interaction all had a highly significant effect on total lipid content (*p* < 0.001). Analysis of the main effects revealed that total lipid content decreased significantly as the treatment concentration increased (*p* < 0.001). Furthermore, the overall total lipid content in the composite organic acid salt group was significantly lower than in any of the single-agent treatment groups (*p* < 0.001). A simple effects analysis confirmed that the inhibitory effect of the composite organic acid salt was superior at all tested concentrations. At 1/4 MIC, the total lipid content in the composite treatment group was 56.92, 41.56, and 62.80% lower than in the sodium diacetate, sodium dehydroacetate, and sodium benzoate groups, respectively. At 1/2 MIC, these reductions were 60.33, 39.30, and 53.46%. The advantage of the composite treatment was most pronounced at the MIC, with cuts of 80.68, 49.96, and 61.01%, respectively. Notably, compared to the untreated control, the composite organic acid salt at its MIC significantly reduced total lipid content (Mean Difference = −74.65, 95% CI [−78.83, −70.47]; *p* < 0.001).

**Table 3:** Effects of different treatments on cell integrity (*n* = 3).

Concentration	Type	Total lipids (mg/g)	Ergosterol (%)	Trehalose (ug/mg)
0	CK	80.49 ± 2.41^Aa^	0.251 ± 0.048	46.09 ± 0.19^Aa^
1/4 MIC	Sodium diacetate	45.57 ± 2.32^Cc^	0.245 ± 0.040	34.73 ± 0.79^Bb^
	Sodium dehydroacetate	33.59 ± 1.17^Dd^	0.240 ± 0.036	22.78 ± 0.31^Cc^
	Sodium benzoate	52.77 ± 1.59^Bb^	0.257 ± 0.038	29.26 ± 0.31^Dd^
	Composite organic acid salt	19.63 ± 0.24^Ee^	0.266 ± 0.060	16.41 ± 0.21^Ee^
0	CK	80.49 ± 2.41^Aa^	0.251 ± 0.048	46.09 ± 0.19^Aa^
1/2 MIC	Sodium diacetate	36.83 ± 2.18^Bb^	0.236 ± 0.050	20.95 ± 0.26^Cc^
	Sodium dehydroacetate	24.07 ± 1.01^Cd^	0.232 ± 0.041	20.78 ± 0.37^Cc^
	Sodium benzoate	31.39 ± 1.05^Bc^	0.233 ± 0.035	22.35 ± 0.19^Bb^
	Composite organic acid salt	14.61 ± 0.65^De^	0.238 ± 0.050	14.34 ± 0.14^Dd^
0	CK	80.49 ± 2.41^Aa^	0.251 ± 0.048	46.09 ± 0.19^Aa^
MIC	Sodium diacetate	30.22 ± 2.13^Bb^	0.231 ± 0.049	19.33 ± 0.26^Bb^
	Sodium dehydroacetate	11.67 ± 0.95^Cc^	0.240 ± 0.053	16.31 ± 0.30^Cc^
	Sodium benzoate	14.98 ± 0.90^Cc^	0.235 ± 0.043	16.80 ± 0.51^Cc^
	Composite organic acid salt	5.84 ± 0.24^Dd^	0.215 ± 0.055	14.05 ± 0.31^Dd^
Concentration	0	80.49 ± 2.41^Aa^	0.251 ± 0.048	46.09 ± 0.19^Aa^
	1/4 MIC	37.89 ± 13.20^Bb^	0.252 ± 0.040	25.79 ± 7.19^Bb^
	1/2 MIC	26.72 ± 8.78^Cc^	0.235 ± 0.038	19.61 ± 3.25^Cc^
	MIC	15.68 ± 9.47^Dd^	0.230 ± 0.044	16.62 ± 1.98^Dd^
Type	CK	80.49 ± 2.41^Aa^	0.251 ± 0.048	46.09 ± 0.19^Aa^
	Sodium diacetate	37.54 ± 6.94^Bb^	0.237 ± 0.041	25.00 ± 7.34^Bb^
	Sodium dehydroacetate	23.11 ± 9.56^Dd^	0.238 ± 0.038	19.96 ± 2.89^Dd^
	Sodium benzoate	33.05 ± 16.44^Cc^	0.242 ± 0.036	22.80 ± 5.41^Cc^
	Composite organic acid salt	13.36 ± 6.05^Ee^	0.240 ± 0.053	14.93 ± 1.13^Ee^
*p*-value	Concentration	< 0.001	0.491	<0.001
	Type	< 0.001	0.997	<0.001
	Concentration × Type	< 0.001	0.982	<0.001

Indicators of cell integrity were analyzed using two-way ANOVA, and results are presented as Mean ± Standard Deviation. with multiple comparisons performed using Tukey’s test. *p* < 0.05 is significant. Different lowercase letters within the same column indicate a significant difference (*p* < 0.05); different uppercase letters indicate a highly significant difference (*p* < 0.01).

A two-way ANOVA of the ergosterol mass fraction revealed no significant interaction effect between organic acid salt concentration and type (*p* = 0.982). Likewise, the main effects for both concentration (*p* = 0.491) and type (*p* = 0.997) were not significant. Although statistical significance was not reached, a descriptive analysis indicated a numerical decrease of 8.73% in ergosterol content as the concentration increased from 1/4 MIC to MIC. Similarly, the ergosterol mass fraction in the treatment groups was numerically slightly lower (ranging from 3.59 to 5.58%) than that of the control group, but these differences fell within the margin of statistical error.

The trend observed for trehalose content was similar to that of total lipids. The concentration, type of organic acid salt, and their interaction also had a highly significant effect on trehalose levels (*p* < 0.001). Trehalose content decreased significantly with increasing concentration (*p* < 0.001), and the inhibitory effect of the composite organic acid salt was, overall, significantly superior to that of the individual treatments (*p* < 0.001). A simple effects analysis demonstrated that the composite organic acid salt was the most effective agent for reducing trehalose content at all concentrations. Compared to the control, the composite organic acid salt at its MIC significantly decreased trehalose content (Mean Difference = −32.04, 95% CI [−32.93, −31.16]; *p* < 0.001).

In summary, treatment with organic acid salts, particularly the composite organic acid salt, significantly inhibited the biosynthesis of total lipids and trehalose but did not have a statistically significant impact on ergosterol mass fraction.

### 3.5 The effects of different treatments on antioxidant indicators

Table 4 shows that the concentration and type of organic acid salts, as well as their interaction, significantly impacted SOD activity (*p* < 0.001), with no significant interaction observed (*p* = 0.151). SOD activity increased in a dose-dependent manner (*p* < 0.001), with sodium benzoate (Mean = 8.84, 95% CI [5.51, 12.17]) and the composite organic acid salt (Mean = 7.37, 95% CI [4.03, 10.70]) showing significantly higher activity than sodium diacetate (*p* < 0.001). At 1/4 MIC and 1/2 MIC, sodium benzoate had significantly higher SOD activity than sodium diacetate (*p* = 0.001 and *p* = 0.010, respectively). At MIC, the composite treatment showed significantly higher SOD activity than both sodium diacetate (Mean Difference = 10.05, 95% CI [3.35, 16.75]; *p* = 0.004) and the control group (Mean Difference = 34.34, 95% CI [27.64, 41.04]; *p* < 0.001).

**Table 4:** Effects of different treatments on antioxidant indicators (*n* = 3).

Concentration	Type	SOD (U/g FW)	CAT (μmol/min/g FW)	GSH-Px (nmol/min/g FW)	MDA (nmol/g)
0	CK	87.77 ± 1.44 ^Cc^	1387.29 ± 18.86^Aa^	209.04 ± 27.33^Cc^	5.99 ± 0.82^Bb^
1/4 MIC	Sodium diacetate	101.97 ± 0.45^Bb^	1278.54 ± 60.26^ABab^	371.26 ± 41.40^Aa^	11.41 ± 0.74^Aa^
	Sodium dehydroacetate	105.72 ± 2.71^ABab^	882.81 ± 62.58^Cc^	273.01 ± 21.58^ABCbc^	9.83 ± 0.29^Aa^
	Sodium benzoate	110.62 ± 0.45^Aa^	1371.99 ± 19.29^Aa^	349.80 ± 33.61^ABab^	9.27 ± 1.39^ABa^
	Composite organic acid salt	106.91 ± 2.72^ABab^	1105.86 ± 120.66^BCb^	251.05 ± 24.62^BCc^	11.18 ± 1.49^Aa^
0	CK	87.77 ± 1.44 ^Cc^	1387.29 ± 18.86^Aa^	209.04 ± 27.33^Bb^	5.99 ± 0.82^Bb^
1/2 MIC	Sodium diacetate	106.05 ± 3.46^Bb^	825.91 ± 13.00^Dd^	403.84 ± 80.25^Aa^	11.72 ± 0.64^ABa^
	Sodium dehydroacetate	107.23 ± 0.59^ABab^	850.78 ± 53.14^CDd^	283.60 ± 28.36^ABbc^	11.11 ± 2.50^ABa^
	Sodium benzoate	116.46 ± 2.34^Aa^	1130.00 ± 21.14^Bb^	408.10 ± 22.81^Aa^	10.88 ± 2.22^ABa^
	Composite organic acid salt	113.16 ± 3.42^ABab^	943.50 ± 9.50^Cc^	355.00 ± 10.47^ABab^	12.14 ± 1.60^Aa^
0	CK	87.77 ± 1.44 ^Cc^	1387.29 ± 18.86^Aa^	209.04 ± 27.33^Cc^	5.99 ± 0.82^Bb^
MIC	Sodium diacetate	112.06 ± 2.42^Bb^	771.06 ± 23.03^Bb^	625.68 ± 58.06^ABb^	13.84 ± 0.29^Aa^
	Sodium dehydroacetate	116.18 ± 0.88^ABab^	734.32 ± 12.35^Bbc^	298.25 ± 31.96^Cc^	13.26 ± 1.55^Aa^
	Sodium benzoate	119.52 ± 1.7^ABa^	780.36 ± 35.11^Bb^	597.40 ± 16.59^Bb^	13.82 ± 1.05^Aa^
	Composite organic acid salt	122.11 ± 4.39^Aa^	692.25 ± 35.27^Bc^	732.73 ± 17.39^Aa^	14.73 ± 2.45^Aa^
Concentration	CK	87.77 ± 1.44^Cd^	1387.29 ± 18.86^Aa^	209.04 ± 27.33^Cc^	5.99 ± 0.82^Cc^
	1/4 MIC	106.31 ± 3.63Bc	1159.80 ± 204.77^Bb^	311.28 ± 59.05^Bb^	10.42 ± 1.32^Bb^
	1/2 MIC	110.73 ± 5.02^Bb^	937.55 ± 127.30^Cc^	357.63 ± 65.90^Bb^	11.46 ± 1.68^ABb^
	MIC	117.47 ± 4.55^Aa^	744.50 ± 43.48^Dd^	563.52 ± 171.09^Aa^	13.91 ± 1.43^Aa^
Type	CK	87.77 ± 1.44^Dc^	1387.29 ± 18.86^Aa^	209.04 ± 27.33^Cc^	5.99 ± 0.82^Bb^
	Sodium diacetate	106.69 ± 4.88^Cb^	958.51 ± 243.44^Cc^	466.92 ± 131.37^Aa^	12.32 ± 1.25^Aa^
	Sodium dehydroacetate	109.71 ± 5.11^BCb^	822.64 ± 79.39^Dd^	284.95 ± 26.33^Bb^	11.40 ± 2.10^Aa^
	Sodium benzoate	115.54 ± 4.19^Aa^	1094.12 ± 258.59^Bb^	451.77 ± 114.23^Aa^	11.32 ± 2.45^Aa^
	Composite organic acid salt	114.06 ± 7.30^ABa^	913.87 ± 191.16^CDc^	439.59 ± 233.41^Aa^	12.68 ± 2.29^Aa^
*p*-value	Concentration	<0.001	<0.001	<0.001	<0.001
	Type	<0.001	<0.001	<0.001	0.165
	Concentration × Type	0.151	<0.001	<0.001	0.945

Indicators of antioxidant indicators were analyzed using two-way ANOVA, and results are presented as Mean ± Standard Deviation with multiple comparisons performed using Tukey’s test. *p* < 0.05 is significant. Different lowercase letters within the same column indicate a significant difference (*p* < 0.05); different uppercase letters indicate a highly significant difference (*p* < 0.01).

Regarding CAT activity, both concentration and type of organic acid salts significantly influenced activity (*p* < 0.001), with a dose-dependent inhibitory effect (*p* < 0.001). At 1/4 MIC, sodium dehydroacetate exerted the strongest inhibition, significantly reducing CAT activity compared to sodium diacetate and sodium benzoate (*p* < 0.001). At this concentration, the composite treatment had significantly lower CAT activity than sodium benzoate (Mean Difference = −266.13, 95% CI [−447.72, −84.54]; *p* = 0.005). At 1/2 MIC, sodium diacetate exhibited the lowest CAT activity, significantly lower than sodium benzoate (*p* < 0.001) and the composite salt (*p* = 0.003). At MIC, the composite treatment showed the most prominent inhibitory effect, with CAT activity significantly lower than both sodium diacetate (*p* = 0.029) and sodium benzoate (*p* = 0.015), and profoundly suppressed compared to the control group (Mean Difference = −695.03, 95% CI [−766.28, −623.79]; *p* < 0.001).

For GSH-Px activity, both concentration and salt type significantly affected activity (*p* < 0.001), with the highest activity at MIC (*p* < 0.001). Sodium diacetate, sodium benzoate, and the composite treatment all showed significantly higher GSH-Px activity compared to sodium dehydroacetate and the control (*p* < 0.001). At 1/4 MIC, sodium diacetate led to substantially higher GSH-Px activity than the composite treatment (Mean Difference = 120.21, 95% CI [38.16, 202.26]; *p* = 0.005). At MIC, both sodium diacetate and composite treatments exhibited significantly higher GSH-Px activity than sodium dehydroacetate (*p* < 0.001), and the composite salt was also considerably more active than sodium benzoate (*p* = 0.004) and the control (*p* < 0.001).

MDA content was significantly affected by concentration alone (*p* < 0.001), with no significant impact from salt type (*p* = 0.165) or interaction (*p* = 0.945). MDA levels increased with concentration, with the MIC showing significantly higher levels than 1/4 MIC (Mean Difference = 3.49, 95% CI [1.81, 5.17]; *p* < 0.001). All treatments resulted in higher MDA levels compared to the control group (*p* < 0.001).

In summary, organic acid salts significantly affected intracellular antioxidant enzyme activities (SOD, CAT, GSH-Px) and promoted lipid peroxidation, as indicated by increased MDA levels. These effects were strongly concentration-dependent, with SOD and GSH-Px activity enhanced and CAT activity suppressed at higher concentrations.

### 3.6 The effect of composite organic salt on the expression levels of key genes involved in the synthesis of aflatoxins

**Figure 6:**
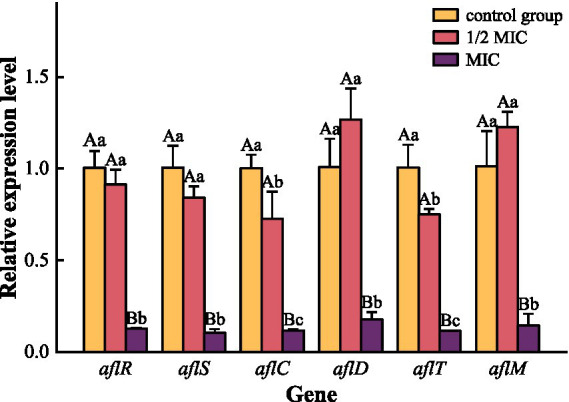
Effects of composite organic acid salt on the expression of key genes involved in aflatoxin biosynthesis. MIC, minimum inhibitory concentration. Gene expression levels were normalized using the *β-Tubulin* gene, with the untreated control group set as the calibrator (relative expression level = 1). The relative expression levels of key genes were analyzed using one-way ANOVA and are presented as Mean ± Standard Deviation, with multiple comparisons performed using Tukey’s test. *p* < 0.05 is significant. For the same gene, different lowercase letters between groups indicate a significant difference (*p* < 0.05), while different uppercase letters indicate a highly significant difference (*p* < 0.01).

The effect of the composite organic acid salt on the expression levels of six key genes within the aflatoxin biosynthesis pathway is presented in Figure 6. At the 1/2 MIC concentration, the expression of *aflC* (Mean Difference = −0.028, 95% CI [−0.51, −0.04]; *p* = 0.028) and *aflT* (Mean Difference = −0.025, 95% CI [−0.44, −0.07]; *p* = 0.013) was significantly downregulated compared to the untreated control group. The expression of the other tested genes did not differ significantly from the control at this concentration (*p* > 0.05). At the MIC, a more profound suppressive effect was observed. The expression of all six target genes—*aflR*, *aflS*, *aflC*, *aflD*, *aflT*, and *aflM*—was highly significantly downregulated (*p* < 0.001) in comparison to both the control group and the 1/2 MIC treatment group.

## 4 Discussion

Inhibiting mold growth and toxin production in feed is crucial for improving feed quality and ensuring animal health and growth. Using food additives or preservatives to suppress Aspergillus growth and aflatoxin production has proven effective in preventing mycotoxin contamination (48). Sodium diacetate works by releasing acetic acid, disrupting the pH balance and metabolic processes within microbial cells (49). Sodium dehydroacetate affects microbial cell membranes and energy metabolism, inhibiting growth (23). Sodium benzoate alters the internal environment of fungal cells, suppressing mold growth (50). In this study, all five organic acid salts showed concentration-dependent inhibitory effects on *A. flavus*. The inhibition was significantly enhanced when sodium diacetate was combined with other salts, particularly when paired with sodium dehydroacetate and potassium sorbate. This synergistic effect suggests that the combination of salts targets different stages or mechanisms of fungal growth, resulting in a multi-level inhibitory action.

The dry mycelial weight of *A. flavus* serves as a useful metric for assessing fungal growth under varying inhibitor conditions (51). Previous studies have demonstrated that treatment with different inhibitors significantly reduces the mycelial dry weight of *A. flavus* (43). This study confirms that the addition of organic acid salts substantially decreases mycelial dry weight, with the most pronounced effect observed under the MIC treatment of compound organic acid salts. This effect is likely due to the disruption of cell integrity by the organic acid salts (52), which in turn affects the fungal adaptability and metabolic activity.

Extracellular relative conductivity and pH changes are commonly used indicators to assess the impact of inhibitors on membrane permeability. Our results show that treatment with compound organic acid salts leads to a general increase in extracellular conductivity and a decrease in pH, a pattern consistent with the mode of action of antifungal agents such as dehydrocorydaline (43). This phenomenon may involve a dual effect. On one hand, it is related to the specific ionic impact of the compound organic acid salts, where undissociated molecules enter the cell and dissociate, generating acid anions and protons (53). The accumulation of acid anions increases intracellular osmotic pressure, forcing the cell to release charged ions, which raises relative conductivity (54). On the other hand, the dissociated protons cannot freely diffuse across the membrane, causing proton accumulation within the cell and thus lowering intracellular pH (55). Additionally, the introduction of organic acid salts leads to medium acidification, which may further stress the structure and stability of the cell membrane. Therefore, the observed increase in conductivity is likely a combined result of these two effects.

Lipids, ergosterol, and trehalose are critical components of the fungal cell membrane, regulating its fluidity and stability, facilitating substance transport, and helping the cell adapt to environmental stress (56). Previous research has shown that treating *A. flavus* with various inhibitors leads to a decrease in the content of these components (43). In the present study, treatment with organic acid salts significantly inhibited the synthesis of total lipids and trehalose, with the composite organic acid salt being the most effective. However, no significant impact on the ergosterol mass fraction was observed. The inhibition of lipid and trehalose synthesis may occur because the organic acid molecules disrupt the fungal cell structure and interfere with the normal tricarboxylic acid (TCA) cycle, thereby affecting osmotic balance (57). Ergosterol is a core component essential for maintaining the structural integrity of the cell membrane, and its homeostasis is critical for cell survival (58). It is plausible that under stress, the cell prioritizes the allocation of its limited resources to maintain ergosterol synthesis.

Antioxidant enzymes such as SOD, CAT, and GSH-Px play a crucial role in effectively scavenging reactive oxygen species (ROS) generated by metabolic activities, thus protecting biomolecules from oxidative damage caused by ROS radicals (59). MDA, a significant product of lipid peroxidation, serves as an indicator of the extent of oxidative damage to cells (60). Previous studies have shown that, upon treatment with inhibitors, *A. flavus* exhibits significantly increased SOD activity and MDA levels. In contrast, CAT activity decreases markedly, with no significant changes in GSH-Px activity (43). In this study, treatment with organic acid salts induced severe oxidative stress in *A. flavus*, as evidenced by a significant increase in SOD activity and MDA levels. Crucially, we observed a disruption in the balance of the antioxidant enzyme system: while GSH-Px activity was markedly elevated, CAT activity was significantly suppressed. This response pattern suggests a compensatory mechanism in *A. flavus*, wherein the fungus attempts to mitigate reactive oxygen species (ROS) toxicity by upregulating GSH-Px in response to impaired CAT-mediated hydrogen peroxide detoxification. As the first line of defense against oxidative stress, SOD plays a pivotal role; its elevated activity indicates a substantial accumulation of superoxide anions, which are subsequently dismutated into hydrogen peroxide (H_2_O_2_) and O_2_ (61). However, the observed suppression of CAT activity creates a critical bottleneck in H_2_O_2_ detoxification, as CAT is the primary enzyme responsible for rapidly converting high concentrations of H_2_O_2_ into harmless H_2_O and O_2_ (62). This enzymatic impairment forces the cell to rely more heavily on GSH-Px to reduce the accumulating H_2_O_2_ and other peroxides (63). However, this compensatory response is inefficient and metabolically costly. The eventual failure of the antioxidant defense system is evidenced by the sharp elevation in MDA levels, clearly indicating the occurrence of lipid peroxidation (60, 64). Therefore, composite organic acid salts disrupt the redox balance in *A. flavus* by impairing its antioxidant system, leading to the accumulation of hydrogen peroxide and ultimately causing severe cellular damage.

Aflatoxins are polyphenolic derivatives, and the pathway regulating their synthesis involves at least 27 enzyme-catalyzed steps (65), with up to 30 genes participating. Studies have shown that the *aflR* and *aflS* genes play significant roles in aflatoxin biosynthesis (34, 66). *aflS* may act as a transcriptional enhancer or co-activator of *aflR*, influencing the biosynthesis of aflatoxins (67). The polyketide synthase gene *aflC* catalyzes the formation of the polyketide backbone from acetyl groups, which is then converted into a stable precursor of aflatoxin, norsolorinic acid (NOR). Thakare et al. (68) successfully reduced aflatoxin content in transgenic maize kernels by inhibiting the expression of *aflC*. Studies have confirmed that *aflD* is involved in the conversion of NOR into averantin (AVN) during aflatoxin biosynthesis (35). *aflT* is not regulated by *aflR* and *aflS* but is associated with aflatoxin exocytosis (69), while *aflM* encodes a ketoreductase involved in the conversion of VERA to DMS (70). Numerous studies have shown that the addition of different inhibitors can downregulate the expression of aflatoxin biosynthesis-related genes (71, 72). In this experiment, treatment with compound organic acid salts at 1/2 MIC significantly downregulated the expression of the *aflC* and *aflT* genes. At MIC, the expression of all six selected related genes was markedly downregulated. These results are consistent with those of Moon et al. (17). Compared to the 1/2 MIC concentration, the MIC concentration had a more potent inhibitory effect on aflatoxin production.

To apply the synergistic *in vitro* efficacy of the composite organic acid salts in feed, careful consideration is needed. The study shows that the MIC of the formulation (8 mg/mL) is achievable under laboratory conditions with a 10^7^ spore/mL inoculum. In real feed environments, initial mold contamination is typically lower, suggesting that a dose below the *in vitro* MIC would still be effective, well within regulatory limits. For example, the maximum permitted levels for sodium dehydroacetate (500 mg/kg) and sodium benzoate (1,000 mg/kg) in food imply that a total dose of 1,000 mg/kg (250 mg/kg each of sodium dehydroacetate and sodium benzoate, and 500 mg/kg of sodium diacetate) could provide adequate protection. This hypothesis will be tested in upcoming *in vivo* animal trials.

While this study demonstrates the antifungal activity of a ternary mixture, several limitations must be addressed. First, the antifungal efficacy was assessed using limited methods; future studies should incorporate additional techniques, such as agar radial growth assays or time-kill studies. The synergy conclusion based on FICI would benefit from validation using isobolograms and response surface modeling. Mechanistically, we did not use a buffered or iso-pH system to distinguish between pH effects and the specific actions of the salts. Also, the washing step may not have fully neutralized absorbed acids, potentially affecting antioxidant status measurements. Future research should include a neutralising wash step and utilise methods such as DCFH-DA assays to quantify ROS levels more directly. Gene expression analysis relied on a single reference gene; future studies will employ at least two validated genes for more accurate normalization. Additionally, the study’s external validity is limited as it tested only one *A. flavus* strain. Broader validation with multiple strains from various sources is necessary. In conclusion, this study offers a proof-of-concept for the antifungal activity of the composite formulation under lab conditions, but translating these findings into real-world feed applications requires further trials to optimize performance in realistic feed matrices, where buffering capacity, component interactions, and water activity differ from laboratory conditions.

## 5 Conclusion

Under the conditions of this study, the composite organic acid salt was found to inhibit the growth of *A. flavus* through a multi-target, synergistic mechanism. This mode of action begins by disrupting the structural integrity of the cell membrane and acidifying the extracellular microenvironment. Subsequently, it impairs the metabolic pathways of key substances such as total lipids and trehalose, thereby limiting the fungus’s energy supply and stress response capacity. Concurrently, the treatment induces oxidative stress, leading to an imbalance in the antioxidant enzyme system and the accumulation of lipid peroxidation products. Ultimately, this cascade of effects culminates at the molecular level in the significant downregulation of key genes within the aflatoxin biosynthesis pathway. The most potent antifungal activity was observed with the MIC of the composite organic acid salt containing sodium diacetate, sodium dehydroacetate, and sodium benzoate in a 2:1:1 ratio.

## Data Availability

The datasets presented in this study can be found in online repositories. The names of the repository/repositories and accession number(s) can be found at: 10.6084/m9.figshare.30771230.
