# Supplementary material for: Synergistic inhibition of Aspergillus flavus by organic acid salts: growth, oxidative stress, and aflatoxin gene modulation
Source: Front Vet Sci. 2025 Dec 4;12:1608792. doi: 10.3389/fvets.2025.1608792 (PMC12713322; doi:10.3389/fvets.2025.1608792)
Supplement: Supplementary file 1 [file Data_Sheet_1.docx]

A suspected *Aspergillus flavus* strain, designated X, was isolated from maize. Its colony morphology on PDA medium is shown in Figure a (front and reverse sides). The colony exhibited a dense, velvety texture, generally round and flat in shape. The color transitioned from white to yellow, then yellow-green, and eventually darkened. Based on these morphological features, strain X was preliminarily identified as *A. flavus*.

Genomic DNA of strain X was extracted using a commercial fungal DNA extraction kit. PCR amplification was performed using ITS1 and ITS4 primers with DNA from strain X as the template. Gel electrophoresis results (Figure b) showed a clear, bright band between 500–750 bp, consistent with the expected size range for fungal ITS regions and free of impurities.

To further confirm the identity of the isolate, the PCR product was sequenced by Sangon Biotech (Shanghai) Co., Ltd., and the obtained ITS sequence was submitted to BLAST analysis against the GenBank database. Phylogenetic analysis was performed using the neighbor-joining (N-J) method in MEGA software. As shown in Figure c, strain X clustered closely with *A. flavus*. (GenBank accession no. MT447498.1).

Based on morphological characteristics and ITS sequence analysis, strain X was conclusively identified as *A. flavus*.


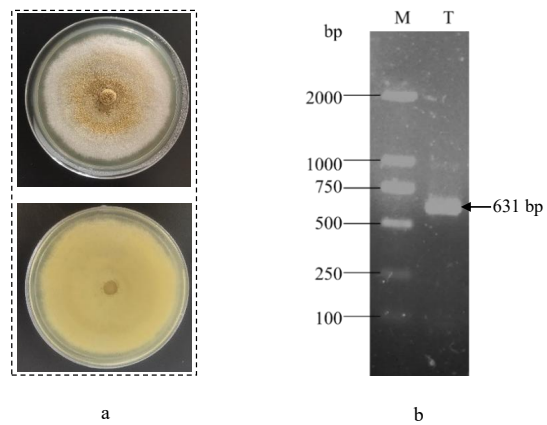

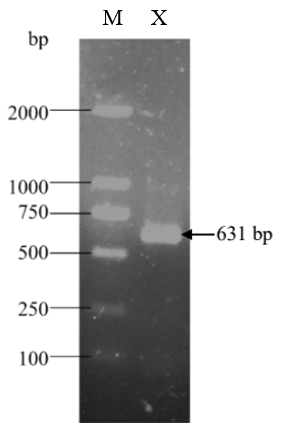


a b


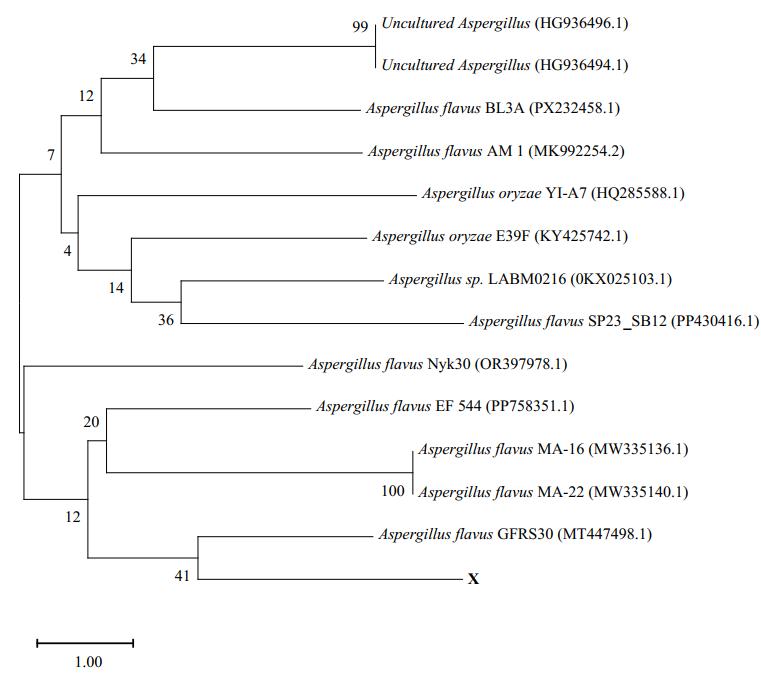


c

# Results of the 95% Confidence Interval

## Mycelium Dry Weight

| Concentration | (A) | (B) | Mean difference  (A-B) | Significance | 95% Confidence interval | |
| --- | --- | --- | --- | --- | --- | --- |
|  |  |  |  |  | Lower bound | Upper bound |
| 1/4 MIC | 0-24 h | 0-48 h | -.80167^*^ | 0.000 | -0.9285 | -0.6749 |
|  |  | Sodium diacetate | 0.04500 | 0.833 | -0.0818 | 0.1718 |
|  |  | Sodium dehydroacetate | -.16333^*^ | 0.010 | -0.2901 | -0.0365 |
|  |  | Sodium benzoate | -0.06500 | 0.544 | -0.1918 | 0.0618 |
|  |  | Composite organic acid salt | 0.00667 | 1.000 | -0.1201 | 0.1335 |
|  | 0-48 h | 0-24 h | .80167^*^ | 0.000 | 0.6749 | 0.9285 |
|  |  | Sodium diacetate | .84667^*^ | 0.000 | 0.7199 | 0.9735 |
|  |  | Sodium dehydroacetate | .63833^*^ | 0.000 | 0.5115 | 0.7651 |
|  |  | Sodium benzoate | .73667^*^ | 0.000 | 0.6099 | 0.8635 |
|  |  | Composite organic acid salt | .80833^*^ | 0.000 | 0.6815 | 0.9351 |
|  | Sodium diacetate | 0-24 h | -0.04500 | 0.833 | -0.1718 | 0.0818 |
|  |  | 0-48 h | -.84667^*^ | 0.000 | -0.9735 | -0.7199 |
|  |  | Sodium dehydroacetate | -.20833^*^ | 0.001 | -0.3351 | -0.0815 |
|  |  | Sodium benzoate | -0.11000 | 0.104 | -0.2368 | 0.0168 |
|  |  | Composite organic acid salt | -0.03833 | 0.904 | -0.1651 | 0.0885 |
|  | Sodium dehydroacetate | 0-24 h | .16333^*^ | 0.010 | 0.0365 | 0.2901 |
|  |  | 0-48 h | -.63833^*^ | 0.000 | -0.7651 | -0.5115 |
|  |  | Sodium diacetate | .20833^*^ | 0.001 | 0.0815 | 0.3351 |
|  |  | Sodium benzoate | 0.09833 | 0.169 | -0.0285 | 0.2251 |
|  |  | Composite organic acid salt | .17000^*^ | 0.007 | 0.0432 | 0.2968 |
|  | Sodium benzoate | 0-24 h | 0.06500 | 0.544 | -0.0618 | 0.1918 |
|  |  | 0-48 h | -.73667^*^ | 0.000 | -0.8635 | -0.6099 |
|  |  | Sodium diacetate | 0.11000 | 0.104 | -0.0168 | 0.2368 |
|  |  | Sodium dehydroacetate | -0.09833 | 0.169 | -0.2251 | 0.0285 |
|  |  | Composite organic acid salt | 0.07167 | 0.447 | -0.0551 | 0.1985 |
|  | Composite organic acid salt | 0-24 h | -0.00667 | 1.000 | -0.1335 | 0.1201 |
|  |  | 0-48 h | -.80833^*^ | 0.000 | -0.9351 | -0.6815 |
|  |  | Sodium diacetate | 0.03833 | 0.904 | -0.0885 | 0.1651 |
|  |  | Sodium dehydroacetate | -.17000^*^ | 0.007 | -0.2968 | -0.0432 |
|  |  | Sodium benzoate | -0.07167 | 0.447 | -0.1985 | 0.0551 |
| 1/2 MIC | 0-24 h | 0-48 h | -.80167^*^ | 0.000 | -0.9278 | -0.6755 |
|  |  | Sodium diacetate | 0.04833 | 0.786 | -0.0778 | 0.1745 |
|  |  | Sodium dehydroacetate | -0.01667 | 0.997 | -0.1428 | 0.1095 |
|  |  | Sodium benzoate | -0.02667 | 0.977 | -0.1528 | 0.0995 |
|  |  | Composite organic acid salt | 0.07167 | 0.442 | -0.0545 | 0.1978 |
|  | 0-48 h | 0-24 h | .80167^*^ | 0.000 | 0.6755 | 0.9278 |
|  |  | Sodium diacetate | .85000^*^ | 0.000 | 0.7239 | 0.9761 |
|  |  | Sodium dehydroacetate | .78500^*^ | 0.000 | 0.6589 | 0.9111 |
|  |  | Sodium benzoate | .77500^*^ | 0.000 | 0.6489 | 0.9011 |
|  |  | Composite organic acid salt | .87333^*^ | 0.000 | 0.7472 | 0.9995 |
|  | Sodium diacetate | 0-24 h | -0.04833 | 0.786 | -0.1745 | 0.0778 |
|  |  | 0-48 h | -.85000^*^ | 0.000 | -0.9761 | -0.7239 |
|  |  | Sodium dehydroacetate | -0.06500 | 0.539 | -0.1911 | 0.0611 |
|  |  | Sodium benzoate | -0.07500 | 0.397 | -0.2011 | 0.0511 |
|  |  | Composite organic acid salt | 0.02333 | 0.987 | -0.1028 | 0.1495 |
|  | Sodium dehydroacetate | 0-24 h | 0.01667 | 0.997 | -0.1095 | 0.1428 |
|  |  | 0-48 h | -.78500^*^ | 0.000 | -0.9111 | -0.6589 |
|  |  | Sodium diacetate | 0.06500 | 0.539 | -0.0611 | 0.1911 |
|  |  | Sodium benzoate | -0.01000 | 1.000 | -0.1361 | 0.1161 |
|  |  | Composite organic acid salt | 0.08833 | 0.246 | -0.0378 | 0.2145 |
|  | Sodium benzoate | 0-24 h | 0.02667 | 0.977 | -0.0995 | 0.1528 |
|  |  | 0-48 h | -.77500^*^ | 0.000 | -0.9011 | -0.6489 |
|  |  | Sodium diacetate | 0.07500 | 0.397 | -0.0511 | 0.2011 |
|  |  | Sodium dehydroacetate | 0.01000 | 1.000 | -0.1161 | 0.1361 |
|  |  | Composite organic acid salt | 0.09833 | 0.166 | -0.0278 | 0.2245 |
|  | Composite organic acid salt | 0-24 h | -0.07167 | 0.442 | -0.1978 | 0.0545 |
|  |  | 0-48 h | -.87333^*^ | 0.000 | -0.9995 | -0.7472 |
|  |  | Sodium diacetate | -0.02333 | 0.987 | -0.1495 | 0.1028 |
|  |  | Sodium dehydroacetate | -0.08833 | 0.246 | -0.2145 | 0.0378 |
|  |  | Sodium benzoate | -0.09833 | 0.166 | -0.2245 | 0.0278 |
| MIC | 0-24 h | 0-48 h | -.80167^*^ | 0.000 | -0.9290 | -0.6744 |
|  |  | Sodium diacetate | 0.09667 | 0.184 | -0.0306 | 0.2240 |
|  |  | Sodium dehydroacetate | 0.04500 | 0.835 | -0.0823 | 0.1723 |
|  |  | Sodium benzoate | 0.05500 | 0.698 | -0.0723 | 0.1823 |
|  |  | Composite organic acid salt | .13833^*^ | 0.031 | 0.0110 | 0.2656 |
|  | 0-48 h | 0-24 h | .80167^*^ | 0.000 | 0.6744 | 0.9290 |
|  |  | Sodium diacetate | .89833^*^ | 0.000 | 0.7710 | 1.0256 |
|  |  | Sodium dehydroacetate | .84667^*^ | 0.000 | 0.7194 | 0.9740 |
|  |  | Sodium benzoate | .85667^*^ | 0.000 | 0.7294 | 0.9840 |
|  |  | Composite organic acid salt | .94000^*^ | 0.000 | 0.8127 | 1.0673 |
|  | Sodium diacetate | 0-24 h | -0.09667 | 0.184 | -0.2240 | 0.0306 |
|  |  | 0-48 h | -.89833^*^ | 0.000 | -1.0256 | -0.7710 |
|  |  | Sodium dehydroacetate | -0.05167 | 0.747 | -0.1790 | 0.0756 |
|  |  | Sodium benzoate | -0.04167 | 0.872 | -0.1690 | 0.0856 |
|  |  | Composite organic acid salt | 0.04167 | 0.872 | -0.0856 | 0.1690 |
|  | Sodium dehydroacetate | 0-24 h | -0.04500 | 0.835 | -0.1723 | 0.0823 |
|  |  | 0-48 h | -.84667^*^ | 0.000 | -0.9740 | -0.7194 |
|  |  | Sodium diacetate | 0.05167 | 0.747 | -0.0756 | 0.1790 |
|  |  | Sodium benzoate | 0.01000 | 1.000 | -0.1173 | 0.1373 |
|  |  | Composite organic acid salt | 0.09333 | 0.210 | -0.0340 | 0.2206 |
|  | Sodium benzoate | 0-24 h | -0.05500 | 0.698 | -0.1823 | 0.0723 |
|  |  | 0-48 h | -.85667^*^ | 0.000 | -0.9840 | -0.7294 |
|  |  | Sodium diacetate | 0.04167 | 0.872 | -0.0856 | 0.1690 |
|  |  | Sodium dehydroacetate | -0.01000 | 1.000 | -0.1373 | 0.1173 |
|  |  | Composite organic acid salt | 0.08333 | 0.305 | -0.0440 | 0.2106 |
|  | Composite organic acid salt | 0-24 h | -.13833^*^ | 0.031 | -0.2656 | -0.0110 |
|  |  | 0-48 h | -.94000^*^ | 0.000 | -1.0673 | -0.8127 |
|  |  | Sodium diacetate | -0.04167 | 0.872 | -0.1690 | 0.0856 |
|  |  | Sodium dehydroacetate | -0.09333 | 0.210 | -0.2206 | 0.0340 |
|  |  | Sodium benzoate | -0.08333 | 0.305 | -0.2106 | 0.0440 |
| *. The significance level of the mean difference is 0.05. | | | | | | |

## Total Lipids

| CK--1/4 MIC | | | | | |
| --- | --- | --- | --- | --- | --- |
| (A) | (B) | Mean difference  (A-B) | Significance | 95% Confidence interval | |
|  |  |  |  | Lower bound | Upper bound |
| CK | Sodium diacetate | 34.92596^*^ | 0.000 | 30.2551 | 39.5968 |
|  | Sodium dehydroacetate | 46.90523^*^ | 0.000 | 42.2344 | 51.5761 |
|  | Sodium benzoate | 27.72444^*^ | 0.000 | 23.0536 | 32.3953 |
|  | Composite organic acid salt | 60.86183^*^ | 0.000 | 56.1910 | 65.5327 |
| Sodium diacetate | CK | -34.92596^*^ | 0.000 | -39.5968 | -30.2551 |
|  | Sodium dehydroacetate | 11.97927^*^ | 0.000 | 7.3084 | 16.6501 |
|  | Sodium benzoate | -7.20152^*^ | 0.003 | -11.8724 | -2.5307 |
|  | Composite organic acid salt | 25.93587^*^ | 0.000 | 21.2650 | 30.6067 |
| Sodium dehydroacetate | CK | -46.90523^*^ | 0.000 | -51.5761 | -42.2344 |
|  | Sodium diacetate | -11.97927^*^ | 0.000 | -16.6501 | -7.3084 |
|  | Sodium benzoate | -19.18080^*^ | 0.000 | -23.8517 | -14.5099 |
|  | Composite organic acid salt | 13.95659^*^ | 0.000 | 9.2857 | 18.6275 |
| Sodium benzoate | CK | -27.72444^*^ | 0.000 | -32.3953 | -23.0536 |
|  | Sodium diacetate | 7.20152^*^ | 0.003 | 2.5307 | 11.8724 |
|  | Sodium dehydroacetate | 19.18080^*^ | 0.000 | 14.5099 | 23.8517 |
|  | Composite organic acid salt | 33.13739^*^ | 0.000 | 28.4665 | 37.8083 |
| Composite organic acid salt | CK | -60.86183^*^ | 0.000 | -65.5327 | -56.1910 |
|  | Sodium diacetate | -25.93587^*^ | 0.000 | -30.6067 | -21.2650 |
|  | Sodium dehydroacetate | -13.95659^*^ | 0.000 | -18.6275 | -9.2857 |
|  | Sodium benzoate | -33.13739^*^ | 0.000 | -37.8083 | -28.4665 |
| *. The significance level of the mean difference is 0.05. | | | | | |

| CK--1/2 MIC | | | | | |
| --- | --- | --- | --- | --- | --- |
| (A) | (B) | Mean difference (A-B) | Significance | 95% Confidence interval | |
|  |  |  |  | Lower bound | Upper bound |
| CK | Sodium diacetate | 43.66261^*^ | 0.000 | 39.3102 | 48.0150 |
|  | Sodium dehydroacetate | 56.42628^*^ | 0.000 | 52.0739 | 60.7786 |
|  | Sodium benzoate | 49.09991^*^ | 0.000 | 44.7475 | 53.4523 |
|  | Composite organic acid salt | 65.87992^*^ | 0.000 | 61.5275 | 70.2323 |
| Sodium diacetate | CK | -43.66261^*^ | 0.000 | -48.0150 | -39.3102 |
|  | Sodium dehydroacetate | 12.76367^*^ | 0.000 | 8.4113 | 17.1160 |
|  | Sodium benzoate | 5.43730^*^ | 0.014 | 1.0849 | 9.7897 |
|  | Composite organic acid salt | 22.21731^*^ | 0.000 | 17.8649 | 26.5697 |
| Sodium dehydroacetate | CK | -56.42628^*^ | 0.000 | -60.7786 | -52.0739 |
|  | Sodium diacetate | -12.76367^*^ | 0.000 | -17.1160 | -8.4113 |
|  | Sodium benzoate | -7.32637^*^ | 0.002 | -11.6787 | -2.9740 |
|  | Composite organic acid salt | 9.45364^*^ | 0.000 | 5.1013 | 13.8060 |
| Sodium benzoate | CK | -49.09991^*^ | 0.000 | -53.4523 | -44.7475 |
|  | Sodium diacetate | -5.43730^*^ | 0.014 | -9.7897 | -1.0849 |
|  | Sodium dehydroacetate | 7.32637^*^ | 0.002 | 2.9740 | 11.6787 |
|  | Composite organic acid salt | 16.78001^*^ | 0.000 | 12.4276 | 21.1324 |
| Composite organic acid salt | CK | -65.87992^*^ | 0.000 | -70.2323 | -61.5275 |
|  | Sodium diacetate | -22.21731^*^ | 0.000 | -26.5697 | -17.8649 |
|  | Sodium dehydroacetate | -9.45364^*^ | 0.000 | -13.8060 | -5.1013 |
|  | Sodium benzoate | -16.78001^*^ | 0.000 | -21.1324 | -12.4276 |
| *. The significance level of the mean difference is 0.05. | | | | | |

| CK--MIC | | | | | |
| --- | --- | --- | --- | --- | --- |
| (A) | (B) | Mean difference  (A-B) | Significance | 95% Confidence interval | |
|  |  |  |  | Lower bound | Upper bound |
| CK | Sodium diacetate | 50.27052^*^ | 0.000 | 46.0908 | 54.4502 |
|  | Sodium dehydroacetate | 68.82166^*^ | 0.000 | 64.6420 | 73.0014 |
|  | Sodium benzoate | 65.50674^*^ | 0.000 | 61.3270 | 69.6865 |
|  | Composite organic acid salt | 74.64972^*^ | 0.000 | 70.4700 | 78.8294 |
| Sodium diacetate | CK | -50.27052^*^ | 0.000 | -54.4502 | -46.0908 |
|  | Sodium dehydroacetate | 18.55114^*^ | 0.000 | 14.3714 | 22.7308 |
|  | Sodium benzoate | 15.23622^*^ | 0.000 | 11.0565 | 19.4159 |
|  | Composite organic acid salt | 24.37919^*^ | 0.000 | 20.1995 | 28.5589 |
| Sodium dehydroacetate | CK | -68.82166^*^ | 0.000 | -73.0014 | -64.6420 |
|  | Sodium diacetate | -18.55114^*^ | 0.000 | -22.7308 | -14.3714 |
|  | Sodium benzoate | -3.31492 | 0.142 | -7.4946 | 0.8648 |
|  | Composite organic acid salt | 5.82805^*^ | 0.007 | 1.6483 | 10.0078 |
| Sodium benzoate | CK | -65.50674^*^ | 0.000 | -69.6865 | -61.3270 |
|  | Sodium diacetate | -15.23622^*^ | 0.000 | -19.4159 | -11.0565 |
|  | Sodium dehydroacetate | 3.31492 | 0.142 | -0.8648 | 7.4946 |
|  | Composite organic acid salt | 9.14297^*^ | 0.000 | 4.9633 | 13.3227 |
| Composite organic acid salt | CK | -74.64972^*^ | 0.000 | -78.8294 | -70.4700 |
|  | Sodium diacetate | -24.37919^*^ | 0.000 | -28.5589 | -20.1995 |
|  | Sodium dehydroacetate | -5.82805^*^ | 0.007 | -10.0078 | -1.6483 |
|  | Sodium benzoate | -9.14297^*^ | 0.000 | -13.3227 | -4.9633 |
| *. The significance level of the mean difference is 0.05. | | | | | |

| Concentration | | | | | |
| --- | --- | --- | --- | --- | --- |
| (A) | (B) | Mean difference  (A-B) | Significance | 95% Confidence interval | |
|  |  |  |  | Lower bound | Upper bound |
| CK | 1/4 MIC | 42.6044^*^ | 0.000 | 39.9715 | 45.2372 |
|  | 1/2 MIC | 53.7672^*^ | 0.000 | 51.1343 | 56.4000 |
|  | MIC | 64.8122^*^ | 0.000 | 62.1793 | 67.4450 |
| 1/4 MIC | CK | -42.6044^*^ | 0.000 | -45.2372 | -39.9715 |
|  | 1/2 MIC | 11.1628^*^ | 0.000 | 9.4977 | 12.8280 |
|  | MIC | 22.2078^*^ | 0.000 | 20.5426 | 23.8730 |
| 1/2 MIC | CK | -53.7672^*^ | 0.000 | -56.4000 | -51.1343 |
|  | 1/4 MIC | -11.1628^*^ | 0.000 | -12.8280 | -9.4977 |
|  | MIC | 11.0450^*^ | 0.000 | 9.3798 | 12.7101 |
| MIC | CK | -64.8122^*^ | 0.000 | -67.4450 | -62.1793 |
|  | 1/4 MIC | -22.2078^*^ | 0.000 | -23.8730 | -20.5426 |
|  | 1/2 MIC | -11.0450^*^ | 0.000 | -12.7101 | -9.3798 |
| *. The significance level of the mean difference is 0.05. | | | | | |

| Type | | | | | |
| --- | --- | --- | --- | --- | --- |
| (A) | (B) | Mean difference  (A-B) | Significance | 95% Confidence interval | |
|  |  |  |  | Lower bound | Upper bound |
| CK | Sodium diacetate | 42.9530^*^ | 0.000 | 40.0503 | 45.8557 |
|  | Sodium dehydroacetate | 57.3844^*^ | 0.000 | 54.4817 | 60.2871 |
|  | Sodium benzoate | 47.4437^*^ | 0.000 | 44.5410 | 50.3464 |
|  | Composite organic acid salt | 67.1305^*^ | 0.000 | 64.2278 | 70.0332 |
| Sodium diacetate | CK | -42.9530^*^ | 0.000 | -45.8557 | -40.0503 |
|  | Sodium dehydroacetate | 14.4314^*^ | 0.000 | 12.3788 | 16.4839 |
|  | Sodium benzoate | 4.4907^*^ | 0.000 | 2.4381 | 6.5432 |
|  | Composite organic acid salt | 24.1775^*^ | 0.000 | 22.1249 | 26.2300 |
| Sodium dehydroacetate | CK | -57.3844^*^ | 0.000 | -60.2871 | -54.4817 |
|  | Sodium diacetate | -14.4314^*^ | 0.000 | -16.4839 | -12.3788 |
|  | Sodium benzoate | -9.9407^*^ | 0.000 | -11.9932 | -7.8882 |
|  | Composite organic acid salt | 9.7461^*^ | 0.000 | 7.6936 | 11.7986 |
| Sodium benzoate | CK | -47.4437^*^ | 0.000 | -50.3464 | -44.5410 |
|  | Sodium diacetate | -4.4907^*^ | 0.000 | -6.5432 | -2.4381 |
|  | Sodium dehydroacetate | 9.9407^*^ | 0.000 | 7.8882 | 11.9932 |
|  | Composite organic acid salt | 19.6868^*^ | 0.000 | 17.6343 | 21.7393 |
| Composite organic acid salt | CK | -67.1305^*^ | 0.000 | -70.0332 | -64.2278 |
|  | Sodium diacetate | -24.1775^*^ | 0.000 | -26.2300 | -22.1249 |
|  | Sodium dehydroacetate | -9.7461^*^ | 0.000 | -11.7986 | -7.6936 |
|  | Sodium benzoate | -19.6868^*^ | 0.000 | -21.7393 | -17.6343 |
| *. The significance level of the mean difference is 0.05. | | | | | |

## Ergosterol

| CK--1/4 MIC | | | | | |
| --- | --- | --- | --- | --- | --- |
| (A) | (B) | Mean difference (A-B) | Significance | 95% Confidence interval | |
|  |  |  |  | Lower bound | Upper bound |
| CK | Sodium diacetate | 0.00552 | 1.000 | -0.1162 | 0.1273 |
|  | Sodium dehydroacetate | 0.01033 | 0.998 | -0.1114 | 0.1321 |
|  | Sodium benzoate | -0.00621 | 1.000 | -0.1280 | 0.1156 |
|  | Composite organic acid salt | -0.01542 | 0.993 | -0.1372 | 0.1063 |
| Sodium diacetate | CK | -0.00552 | 1.000 | -0.1273 | 0.1162 |
|  | Sodium dehydroacetate | 0.00481 | 1.000 | -0.1170 | 0.1266 |
|  | Sodium benzoate | -0.01173 | 0.997 | -0.1335 | 0.1100 |
|  | Composite organic acid salt | -0.02094 | 0.977 | -0.1427 | 0.1008 |
| Sodium dehydroacetate | CK | -0.01033 | 0.998 | -0.1321 | 0.1114 |
|  | Sodium diacetate | -0.00481 | 1.000 | -0.1266 | 0.1170 |
|  | Sodium benzoate | -0.01654 | 0.990 | -0.1383 | 0.1052 |
|  | Composite organic acid salt | -0.02575 | 0.953 | -0.1475 | 0.0960 |
| Sodium benzoate | CK | 0.00621 | 1.000 | -0.1156 | 0.1280 |
|  | Sodium diacetate | 0.01173 | 0.997 | -0.1100 | 0.1335 |
|  | Sodium dehydroacetate | 0.01654 | 0.990 | -0.1052 | 0.1383 |
|  | Composite organic acid salt | -0.00921 | 0.999 | -0.1310 | 0.1125 |
| Composite organic acid salt | CK | 0.01542 | 0.993 | -0.1063 | 0.1372 |
|  | Sodium diacetate | 0.02094 | 0.977 | -0.1008 | 0.1427 |
|  | Sodium dehydroacetate | 0.02575 | 0.953 | -0.0960 | 0.1475 |
|  | Sodium benzoate | 0.00921 | 0.999 | -0.1125 | 0.1310 |

| CK--1/2 MIC | | | | | |
| --- | --- | --- | --- | --- | --- |
| (A) | (B) | Mean difference (A-B) | Significance | 95% Confidence interval | |
|  |  |  |  | Lower bound | Upper bound |
| CK | Sodium diacetate | 0.01514 | 0.993 | -0.1059 | 0.1362 |
|  | Sodium dehydroacetate | 0.01840 | 0.985 | -0.1026 | 0.1394 |
|  | Sodium benzoate | 0.01755 | 0.988 | -0.1035 | 0.1386 |
|  | Composite organic acid salt | 0.01233 | 0.997 | -0.1087 | 0.1334 |
| Sodium diacetate | CK | -0.01514 | 0.993 | -0.1362 | 0.1059 |
|  | Sodium dehydroacetate | 0.00326 | 1.000 | -0.1178 | 0.1243 |
|  | Sodium benzoate | 0.00241 | 1.000 | -0.1186 | 0.1234 |
|  | Composite organic acid salt | -0.00281 | 1.000 | -0.1238 | 0.1182 |
| Sodium dehydroacetate | CK | -0.01840 | 0.985 | -0.1394 | 0.1026 |
|  | Sodium diacetate | -0.00326 | 1.000 | -0.1243 | 0.1178 |
|  | Sodium benzoate | -0.00085 | 1.000 | -0.1219 | 0.1202 |
|  | Composite organic acid salt | -0.00606 | 1.000 | -0.1271 | 0.1150 |
| Sodium benzoate | CK | -0.01755 | 0.988 | -0.1386 | 0.1035 |
|  | Sodium diacetate | -0.00241 | 1.000 | -0.1234 | 0.1186 |
|  | Sodium dehydroacetate | 0.00085 | 1.000 | -0.1202 | 0.1219 |
|  | Composite organic acid salt | -0.00521 | 1.000 | -0.1263 | 0.1158 |
| Composite organic acid salt | CK | -0.01233 | 0.997 | -0.1334 | 0.1087 |
|  | Sodium diacetate | 0.00281 | 1.000 | -0.1182 | 0.1238 |
|  | Sodium dehydroacetate | 0.00606 | 1.000 | -0.1150 | 0.1271 |
|  | Sodium benzoate | 0.00521 | 1.000 | -0.1158 | 0.1263 |

| CK--MIC | | | | | |
| --- | --- | --- | --- | --- | --- |
| (A) | (B) | Mean difference  (A-B) | Significance | 95% Confidence interval | |
|  |  |  |  | Lower bound | Upper bound |
| CK | Sodium diacetate | 0.01946 | 0.988 | -0.1144 | 0.1533 |
|  | Sodium dehydroacetate | 0.01036 | 0.999 | -0.1235 | 0.1442 |
|  | Sodium benzoate | 0.01579 | 0.994 | -0.1181 | 0.1497 |
|  | Composite organic acid salt | 0.03589 | 0.897 | -0.0980 | 0.1698 |
| Sodium diacetate | CK | -0.01946 | 0.988 | -0.1533 | 0.1144 |
|  | Sodium dehydroacetate | -0.00910 | 0.999 | -0.1430 | 0.1248 |
|  | Sodium benzoate | -0.00367 | 1.000 | -0.1375 | 0.1302 |
|  | Composite organic acid salt | 0.01643 | 0.993 | -0.1174 | 0.1503 |
| Sodium dehydroacetate | CK | -0.01036 | 0.999 | -0.1442 | 0.1235 |
|  | Sodium diacetate | 0.00910 | 0.999 | -0.1248 | 0.1430 |
|  | Sodium benzoate | 0.00543 | 1.000 | -0.1284 | 0.1393 |
|  | Composite organic acid salt | 0.02553 | 0.967 | -0.1083 | 0.1594 |
| Sodium benzoate | CK | -0.01579 | 0.994 | -0.1497 | 0.1181 |
|  | Sodium diacetate | 0.00367 | 1.000 | -0.1302 | 0.1375 |
|  | Sodium dehydroacetate | -0.00543 | 1.000 | -0.1393 | 0.1284 |
|  | Composite organic acid salt | 0.02010 | 0.986 | -0.1138 | 0.1540 |
| Composite organic acid salt | CK | -0.03589 | 0.897 | -0.1698 | 0.0980 |
|  | Sodium diacetate | -0.01643 | 0.993 | -0.1503 | 0.1174 |
|  | Sodium dehydroacetate | -0.02553 | 0.967 | -0.1594 | 0.1083 |
|  | Sodium benzoate | -0.02010 | 0.986 | -0.1540 | 0.1138 |

| Concentration | | | | | |
| --- | --- | --- | --- | --- | --- |
| (A) | (B) | Mean difference  (A-B) | Significance | 95% Confidence interval | |
|  |  |  |  | Lower bound | Upper bound |
| CK | 1/4 MIC | -0.0014 | 1.000 | -0.0840 | 0.0811 |
|  | 1/2 MIC | 0.0159 | 0.952 | -0.0667 | 0.0984 |
|  | MIC | 0.0204 | 0.905 | -0.0622 | 0.1029 |
| 1/4 MIC | CK | 0.0014 | 1.000 | -0.0811 | 0.0840 |
|  | 1/2 MIC | 0.0173 | 0.800 | -0.0349 | 0.0695 |
|  | MIC | 0.0218 | 0.665 | -0.0304 | 0.0740 |
| 1/2 MIC | CK | -0.0159 | 0.952 | -0.0984 | 0.0667 |
|  | 1/4 MIC | -0.0173 | 0.800 | -0.0695 | 0.0349 |
|  | MIC | 0.0045 | 0.995 | -0.0477 | 0.0567 |
| MIC | CK | -0.0204 | 0.905 | -0.1029 | 0.0622 |
|  | 1/4 MIC | -0.0218 | 0.665 | -0.0740 | 0.0304 |
|  | 1/2 MIC | -0.0045 | 0.995 | -0.0567 | 0.0477 |

| Type | | | | | |
| --- | --- | --- | --- | --- | --- |
| (A) | (B) | Mean difference  (A-B) | Significance | 95% Confidence interval | |
|  |  |  |  | Lower bound | Upper bound |
| CK | Sodium diacetate | 0.0134 | 0.992 | -0.0776 | 0.1044 |
|  | Sodium dehydroacetate | 0.0130 | 0.993 | -0.0780 | 0.1040 |
|  | Sodium benzoate | 0.0090 | 0.998 | -0.0820 | 0.1001 |
|  | Composite organic acid salt | 0.0109 | 0.996 | -0.0801 | 0.1020 |
| Sodium diacetate | CK | -0.0134 | 0.992 | -0.1044 | 0.0776 |
|  | Sodium dehydroacetate | -0.0003 | 1.000 | -0.0647 | 0.0640 |
|  | Sodium benzoate | -0.0043 | 1.000 | -0.0687 | 0.0600 |
|  | Composite organic acid salt | -0.0024 | 1.000 | -0.0668 | 0.0619 |
| Sodium dehydroacetate | CK | -0.0130 | 0.993 | -0.1040 | 0.0780 |
|  | Sodium diacetate | 0.0003 | 1.000 | -0.0640 | 0.0647 |
|  | Sodium benzoate | -0.0040 | 1.000 | -0.0683 | 0.0604 |
|  | Composite organic acid salt | -0.0021 | 1.000 | -0.0665 | 0.0623 |
| Sodium benzoate | CK | -0.0090 | 0.998 | -0.1001 | 0.0820 |
|  | Sodium diacetate | 0.0043 | 1.000 | -0.0600 | 0.0687 |
|  | Sodium dehydroacetate | 0.0040 | 1.000 | -0.0604 | 0.0683 |
|  | Composite organic acid salt | 0.0019 | 1.000 | -0.0625 | 0.0663 |
| Composite organic acid salt | CK | -0.0109 | 0.996 | -0.1020 | 0.0801 |
|  | Sodium diacetate | 0.0024 | 1.000 | -0.0619 | 0.0668 |
|  | Sodium dehydroacetate | 0.0021 | 1.000 | -0.0623 | 0.0665 |
|  | Sodium benzoate | -0.0019 | 1.000 | -0.0663 | 0.0625 |

## Trehalose

| CK--1/4 MIC | | | | | |
| --- | --- | --- | --- | --- | --- |
| (A) | (B) | Mean difference  (A-B) | Significance | 95% Confidence interval | |
|  |  |  |  | Lower bound | Upper bound |
| CK | Sodium diacetate | 11.35882^*^ | 0.000 | 10.2005 | 12.5171 |
|  | Sodium dehydroacetate | 23.30691^*^ | 0.000 | 22.1486 | 24.4652 |
|  | Sodium benzoate | 16.83065^*^ | 0.000 | 15.6724 | 17.9889 |
|  | Composite organic acid salt | 29.68346^*^ | 0.000 | 28.5252 | 30.8417 |
| Sodium diacetate | CK | -11.35882^*^ | 0.000 | -12.5171 | -10.2005 |
|  | Sodium dehydroacetate | 11.94809^*^ | 0.000 | 10.7898 | 13.1064 |
|  | Sodium benzoate | 5.47182^*^ | 0.000 | 4.3135 | 6.6301 |
|  | Composite organic acid salt | 18.32463^*^ | 0.000 | 17.1663 | 19.4829 |
| Sodium dehydroacetate | CK | -23.30691^*^ | 0.000 | -24.4652 | -22.1486 |
|  | Sodium diacetate | -11.94809^*^ | 0.000 | -13.1064 | -10.7898 |
|  | Sodium benzoate | -6.47626^*^ | 0.000 | -7.6345 | -5.3180 |
|  | Composite organic acid salt | 6.37655^*^ | 0.000 | 5.2183 | 7.5348 |
| Sodium benzoate | CK | -16.83065^*^ | 0.000 | -17.9889 | -15.6724 |
|  | Sodium diacetate | -5.47182^*^ | 0.000 | -6.6301 | -4.3135 |
|  | Sodium dehydroacetate | 6.47626^*^ | 0.000 | 5.3180 | 7.6345 |
|  | Composite organic acid salt | 12.85281^*^ | 0.000 | 11.6945 | 14.0111 |
| Composite organic acid salt | CK | -29.68346^*^ | 0.000 | -30.8417 | -28.5252 |
|  | Sodium diacetate | -18.32463^*^ | 0.000 | -19.4829 | -17.1663 |
|  | Sodium dehydroacetate | -6.37655^*^ | 0.000 | -7.5348 | -5.2183 |
|  | Sodium benzoate | -12.85281^*^ | 0.000 | -14.0111 | -11.6945 |
| *. The significance level of the mean difference is 0.05. | | | | | |

| CK--1/2 MIC | | | | | |
| --- | --- | --- | --- | --- | --- |
| (A) | (B) | Mean difference  (A-B) | Significance | 95% Confidence interval | |
|  |  |  |  | Lower bound | Upper bound |
| CK | Sodium diacetate | 25.14123^*^ | 0.000 | 24.4964 | 25.7860 |
|  | Sodium dehydroacetate | 25.31060^*^ | 0.000 | 24.6658 | 25.9554 |
|  | Sodium benzoate | 23.73858^*^ | 0.000 | 23.0938 | 24.3834 |
|  | Composite organic acid salt | 31.75281^*^ | 0.000 | 31.1080 | 32.3976 |
| Sodium diacetate | CK | -25.14123^*^ | 0.000 | -25.7860 | -24.4964 |
|  | Sodium dehydroacetate | 0.16937 | 0.903 | -0.4754 | 0.8142 |
|  | Sodium benzoate | -1.40264^*^ | 0.000 | -2.0475 | -0.7578 |
|  | Composite organic acid salt | 6.61159^*^ | 0.000 | 5.9668 | 7.2564 |
| Sodium dehydroacetate | CK | -25.31060^*^ | 0.000 | -25.9554 | -24.6658 |
|  | Sodium diacetate | -0.16937 | 0.903 | -0.8142 | 0.4754 |
|  | Sodium benzoate | -1.57201^*^ | 0.000 | -2.2168 | -0.9272 |
|  | Composite organic acid salt | 6.44221^*^ | 0.000 | 5.7974 | 7.0870 |
| Sodium benzoate | CK | -23.73858^*^ | 0.000 | -24.3834 | -23.0938 |
|  | Sodium diacetate | 1.40264^*^ | 0.000 | 0.7578 | 2.0475 |
|  | Sodium dehydroacetate | 1.57201^*^ | 0.000 | 0.9272 | 2.2168 |
|  | Composite organic acid salt | 8.01423^*^ | 0.000 | 7.3694 | 8.6590 |
| Composite organic acid salt | CK | -31.75281^*^ | 0.000 | -32.3976 | -31.1080 |
|  | Sodium diacetate | -6.61159^*^ | 0.000 | -7.2564 | -5.9668 |
|  | Sodium dehydroacetate | -6.44221^*^ | 0.000 | -7.0870 | -5.7974 |
|  | Sodium benzoate | -8.01423^*^ | 0.000 | -8.6590 | -7.3694 |
| *. The significance level of the mean difference is 0.05. | | | | | |

| CK--MIC | | | | | |
| --- | --- | --- | --- | --- | --- |
| (A) | (B) | Mean difference  (A-B) | Significance | 95% Confidence interval | |
|  |  |  |  | Lower bound | Upper bound |
| CK | Sodium diacetate | 26.76162^*^ | 0.000 | 25.8777 | 27.6456 |
|  | Sodium dehydroacetate | 29.78219^*^ | 0.000 | 28.8982 | 30.6661 |
|  | Sodium benzoate | 29.28890^*^ | 0.000 | 28.4050 | 30.1728 |
|  | Composite organic acid salt | 32.04385^*^ | 0.000 | 31.1599 | 32.9278 |
| Sodium diacetate | CK | -26.76162^*^ | 0.000 | -27.6456 | -25.8777 |
|  | Sodium dehydroacetate | 3.02057^*^ | 0.000 | 2.1366 | 3.9045 |
|  | Sodium benzoate | 2.52728^*^ | 0.000 | 1.6433 | 3.4112 |
|  | Composite organic acid salt | 5.28223^*^ | 0.000 | 4.3983 | 6.1662 |
| Sodium dehydroacetate | CK | -29.78219^*^ | 0.000 | -30.6661 | -28.8982 |
|  | Sodium diacetate | -3.02057^*^ | 0.000 | -3.9045 | -2.1366 |
|  | Sodium benzoate | -0.49330 | 0.406 | -1.3772 | 0.3906 |
|  | Composite organic acid salt | 2.26166^*^ | 0.000 | 1.3777 | 3.1456 |
| Sodium benzoate | CK | -29.28890^*^ | 0.000 | -30.1728 | -28.4050 |
|  | Sodium diacetate | -2.52728^*^ | 0.000 | -3.4112 | -1.6433 |
|  | Sodium dehydroacetate | 0.49330 | 0.406 | -0.3906 | 1.3772 |
|  | Composite organic acid salt | 2.75495^*^ | 0.000 | 1.8710 | 3.6389 |
| Composite organic acid salt | CK | -32.04385^*^ | 0.000 | -32.9278 | -31.1599 |
|  | Sodium diacetate | -5.28223^*^ | 0.000 | -6.1662 | -4.3983 |
|  | Sodium dehydroacetate | -2.26166^*^ | 0.000 | -3.1456 | -1.3777 |
|  | Sodium benzoate | -2.75495^*^ | 0.000 | -3.6389 | -1.8710 |
| *. The significance level of the mean difference is 0.05. | | | | | |

| Concentration | | | | | |
| --- | --- | --- | --- | --- | --- |
| (A) | (B) | Mean difference  (A-B) | Significance | 95% Confidence interval | |
|  |  |  |  | Lower bound | Upper bound |
| CK | 1/4 MIC | 20.2950^*^ | 0.000 | 19.6566 | 20.9333 |
|  | 1/2 MIC | 26.4858^*^ | 0.000 | 25.8474 | 27.1242 |
|  | MIC | 29.4691^*^ | 0.000 | 28.8308 | 30.1075 |
| 1/4 MIC | CK | -20.2950^*^ | 0.000 | -20.9333 | -19.6566 |
|  | 1/2 MIC | 6.1908^*^ | 0.000 | 5.7871 | 6.5946 |
|  | MIC | 9.1742^*^ | 0.000 | 8.7704 | 9.5779 |
| 1/2 MIC | CK | -26.4858^*^ | 0.000 | -27.1242 | -25.8474 |
|  | 1/4 MIC | -6.1908^*^ | 0.000 | -6.5946 | -5.7871 |
|  | MIC | 2.9833^*^ | 0.000 | 2.5796 | 3.3871 |
| MIC | CK | -29.4691^*^ | 0.000 | -30.1075 | -28.8308 |
|  | 1/4 MIC | -9.1742^*^ | 0.000 | -9.5779 | -8.7704 |
|  | 1/2 MIC | -2.9833^*^ | 0.000 | -3.3871 | -2.5796 |
| *. The significance level of the mean difference is 0.05. | | | | | |

| Type | | | | | |
| --- | --- | --- | --- | --- | --- |
| (A) | (B) | Mean difference (A-B) | Significance | 95% Confidence interval | |
|  |  |  |  | Lower bound | Upper bound |
| CK | Sodium diacetate | 21.0872^*^ | 0.000 | 20.3834 | 21.7910 |
|  | Sodium dehydroacetate | 26.1332^*^ | 0.000 | 25.4294 | 26.8370 |
|  | Sodium benzoate | 23.2860^*^ | 0.000 | 22.5823 | 23.9898 |
|  | Composite organic acid salt | 31.1600^*^ | 0.000 | 30.4563 | 31.8638 |
| Sodium diacetate | CK | -21.0872^*^ | 0.000 | -21.7910 | -20.3834 |
|  | Sodium dehydroacetate | 5.0460^*^ | 0.000 | 4.5484 | 5.5437 |
|  | Sodium benzoate | 2.1988^*^ | 0.000 | 1.7012 | 2.6965 |
|  | Composite organic acid salt | 10.0728^*^ | 0.000 | 9.5752 | 10.5705 |
| Sodium dehydroacetate | CK | -26.1332^*^ | 0.000 | -26.8370 | -25.4294 |
|  | Sodium diacetate | -5.0460^*^ | 0.000 | -5.5437 | -4.5484 |
|  | Sodium benzoate | -2.8472^*^ | 0.000 | -3.3448 | -2.3495 |
|  | Composite organic acid salt | 5.0268^*^ | 0.000 | 4.5292 | 5.5245 |
| Sodium benzoate | CK | -23.2860^*^ | 0.000 | -23.9898 | -22.5823 |
|  | Sodium diacetate | -2.1988^*^ | 0.000 | -2.6965 | -1.7012 |
|  | Sodium dehydroacetate | 2.8472^*^ | 0.000 | 2.3495 | 3.3448 |
|  | Composite organic acid salt | 7.8740^*^ | 0.000 | 7.3763 | 8.3716 |
| Composite organic acid salt | CK | -31.1600^*^ | 0.000 | -31.8638 | -30.4563 |
|  | Sodium diacetate | -10.0728^*^ | 0.000 | -10.5705 | -9.5752 |
|  | Sodium dehydroacetate | -5.0268^*^ | 0.000 | -5.5245 | -4.5292 |
|  | Sodium benzoate | -7.8740^*^ | 0.000 | -8.3716 | -7.3763 |
| *. The significance level of the mean difference is 0.05. | | | | | |

## SOD

| CK--1/4 MIC | | | | | |
| --- | --- | --- | --- | --- | --- |
| (A) | (B) | Mean difference (A-B) | Significance | 95% Confidence interval | |
|  |  |  |  | Lower bound | Upper bound |
| CK | Sodium diacetate | -14.20149^*^ | 0.000 | -19.1923 | -9.2107 |
|  | Sodium dehydroacetate | -17.95346^*^ | 0.000 | -22.9442 | -12.9627 |
|  | Sodium benzoate | -22.85629^*^ | 0.000 | -27.8471 | -17.8655 |
|  | Composite organic acid salt | -19.14261^*^ | 0.000 | -24.1334 | -14.1518 |
| Sodium diacetate | CK | 14.20149^*^ | 0.000 | 9.2107 | 19.1923 |
|  | Sodium dehydroacetate | -3.75198 | 0.173 | -8.7428 | 1.2388 |
|  | Sodium benzoate | -8.65480^*^ | 0.001 | -13.6456 | -3.6640 |
|  | Composite organic acid salt | -4.94113 | 0.053 | -9.9319 | 0.0497 |
| Sodium dehydroacetate | CK | 17.95346^*^ | 0.000 | 12.9627 | 22.9442 |
|  | Sodium diacetate | 3.75198 | 0.173 | -1.2388 | 8.7428 |
|  | Sodium benzoate | -4.90283 | 0.055 | -9.8936 | 0.0879 |
|  | Composite organic acid salt | -1.18915 | 0.930 | -6.1799 | 3.8016 |
| Sodium benzoate | CK | 22.85629^*^ | 0.000 | 17.8655 | 27.8471 |
|  | Sodium diacetate | 8.65480^*^ | 0.001 | 3.6640 | 13.6456 |
|  | Sodium dehydroacetate | 4.90283 | 0.055 | -0.0879 | 9.8936 |
|  | Composite organic acid salt | 3.71368 | 0.179 | -1.2771 | 8.7045 |
| Composite organic acid salt | CK | 19.14261^*^ | 0.000 | 14.1518 | 24.1334 |
|  | Sodium diacetate | 4.94113 | 0.053 | -0.0497 | 9.9319 |
|  | Sodium dehydroacetate | 1.18915 | 0.930 | -3.8016 | 6.1799 |
|  | Sodium benzoate | -3.71368 | 0.179 | -8.7045 | 1.2771 |
| *. The significance level of the mean difference is 0.05. | | | | | |

| CK--1/2 MIC | | | | | |
| --- | --- | --- | --- | --- | --- |
| (A) | (B) | Mean difference  (A-B) | Significance | 95% Confidence interval | |
|  |  |  |  | Lower bound | Upper bound |
| CK | Sodium diacetate | -18.92555^*^ | 0.000 | -29.1811 | -8.6700 |
|  | Sodium dehydroacetate | -21.94404^*^ | 0.000 | -32.1995 | -11.6885 |
|  | Sodium benzoate | -27.76988^*^ | 0.000 | -38.0254 | -17.5144 |
|  | Composite organic acid salt | -26.29110^*^ | 0.000 | -36.5466 | -16.0356 |
| Sodium diacetate | CK | 18.92555^*^ | 0.000 | 8.6700 | 29.1811 |
|  | Sodium dehydroacetate | -3.01849 | 0.752 | -10.2702 | 4.2333 |
|  | Sodium benzoate | -8.84433^*^ | 0.010 | -16.0961 | -1.5926 |
|  | Composite organic acid salt | -7.36554^*^ | 0.045 | -14.6173 | -0.1138 |
| Sodium dehydroacetate | CK | 21.94404^*^ | 0.000 | 11.6885 | 32.1995 |
|  | Sodium diacetate | 3.01849 | 0.752 | -4.2333 | 10.2702 |
|  | Sodium benzoate | -5.82584 | 0.165 | -13.0776 | 1.4259 |
|  | Composite organic acid salt | -4.34706 | 0.432 | -11.5988 | 2.9047 |
| Sodium benzoate | CK | 27.76988^*^ | 0.000 | 17.5144 | 38.0254 |
|  | Sodium diacetate | 8.84433^*^ | 0.010 | 1.5926 | 16.0961 |
|  | Sodium dehydroacetate | 5.82584 | 0.165 | -1.4259 | 13.0776 |
|  | Composite organic acid salt | 1.47878 | 0.976 | -5.7730 | 8.7305 |
| Composite organic acid salt | CK | 26.29110^*^ | 0.000 | 16.0356 | 36.5466 |
|  | Sodium diacetate | 7.36554^*^ | 0.045 | 0.1138 | 14.6173 |
|  | Sodium dehydroacetate | 4.34706 | 0.432 | -2.9047 | 11.5988 |
|  | Sodium benzoate | -1.47878 | 0.976 | -8.7305 | 5.7730 |
| *. The significance level of the mean difference is 0.05. | | | | | |

| CK--MIC | | | | | |
| --- | --- | --- | --- | --- | --- |
| (A) | (B) | Mean difference  (A-B) | Significance | 95% Confidence interval | |
|  |  |  |  | Lower bound | Upper bound |
| CK | Sodium diacetate | -24.28892^*^ | 0.000 | -30.9881 | -17.5897 |
|  | Sodium dehydroacetate | -28.41238^*^ | 0.000 | -35.1116 | -21.7132 |
|  | Sodium benzoate | -31.75619^*^ | 0.000 | -38.4554 | -25.0570 |
|  | Composite organic acid salt | -34.33932^*^ | 0.000 | -41.0385 | -27.6401 |
| Sodium diacetate | CK | 24.28892^*^ | 0.000 | 17.5897 | 30.9881 |
|  | Sodium dehydroacetate | -4.12346 | 0.321 | -10.8227 | 2.5758 |
|  | Sodium benzoate | -7.46727^*^ | 0.028 | -14.1665 | -0.7681 |
|  | Composite organic acid salt | -10.05040^*^ | 0.004 | -16.7496 | -3.3512 |
| Sodium dehydroacetate | CK | 28.41238^*^ | 0.000 | 21.7132 | 35.1116 |
|  | Sodium diacetate | 4.12346 | 0.321 | -2.5758 | 10.8227 |
|  | Sodium benzoate | -3.34381 | 0.506 | -10.0430 | 3.3554 |
|  | Composite organic acid salt | -5.92694 | 0.090 | -12.6262 | 0.7723 |
| Sodium benzoate | CK | 31.75619^*^ | 0.000 | 25.0570 | 38.4554 |
|  | Sodium diacetate | 7.46727^*^ | 0.028 | 0.7681 | 14.1665 |
|  | Sodium dehydroacetate | 3.34381 | 0.506 | -3.3554 | 10.0430 |
|  | Composite organic acid salt | -2.58313 | 0.714 | -9.2823 | 4.1161 |
| Composite organic acid salt | CK | 34.33932^*^ | 0.000 | 27.6401 | 41.0385 |
|  | Sodium diacetate | 10.05040^*^ | 0.004 | 3.3512 | 16.7496 |
|  | Sodium dehydroacetate | 5.92694 | 0.090 | -0.7723 | 12.6262 |
|  | Sodium benzoate | 2.58313 | 0.714 | -4.1161 | 9.2823 |
| *. The significance level of the mean difference is 0.05. | | | | | |

| Concentration | | | | | |
| --- | --- | --- | --- | --- | --- |
| (A) | (B) | Mean difference  (A-B) | Significance | 95% Confidence interval | |
|  |  |  |  | Lower bound | Upper bound |
| CK | 1/4 MIC | -18.5385^*^ | 0.000 | -22.8120 | -14.2649 |
|  | 1/2 MIC | -22.9603^*^ | 0.000 | -27.2338 | -18.6867 |
|  | MIC | -29.6992^*^ | 0.000 | -33.9728 | -25.4257 |
| 1/4 MIC | CK | 18.5385^*^ | 0.000 | 14.2649 | 22.8120 |
|  | 1/2 MIC | -4.4218^*^ | 0.001 | -7.1246 | -1.7190 |
|  | MIC | -11.1607^*^ | 0.000 | -13.8636 | -8.4579 |
| 1/2 MIC | CK | 22.9603^*^ | 0.000 | 18.6867 | 27.2338 |
|  | 1/4 MIC | 4.4218^*^ | 0.001 | 1.7190 | 7.1246 |
|  | MIC | -6.7390^*^ | 0.000 | -9.4418 | -4.0361 |
| MIC | CK | 29.6992^*^ | 0.000 | 25.4257 | 33.9728 |
|  | 1/4 MIC | 11.1607^*^ | 0.000 | 8.4579 | 13.8636 |
|  | 1/2 MIC | 6.7390^*^ | 0.000 | 4.0361 | 9.4418 |
| *. The significance level of the mean difference is 0.05. | | | | | |

| Type | | | | | |
| --- | --- | --- | --- | --- | --- |
| (A) | (B) | Mean difference  (A-B) | Significance | 95% Confidence interval | |
|  |  |  |  | Lower bound | Upper bound |
| CK | Sodium diacetate | -18.9256^*^ | 0.000 | -23.6371 | -14.2140 |
|  | Sodium dehydroacetate | -21.9440^*^ | 0.000 | -26.6556 | -17.2325 |
|  | Sodium benzoate | -27.7699^*^ | 0.000 | -32.4814 | -23.0583 |
|  | Composite organic acid salt | -26.2911^*^ | 0.000 | -31.0027 | -21.5795 |
| Sodium diacetate | CK | 18.9256^*^ | 0.000 | 14.2140 | 23.6371 |
|  | Sodium dehydroacetate | -3.0185 | 0.090 | -6.3501 | 0.3131 |
|  | Sodium benzoate | -8.8443^*^ | 0.000 | -12.1759 | -5.5128 |
|  | Composite organic acid salt | -7.3655^*^ | 0.000 | -10.6971 | -4.0340 |
| Sodium dehydroacetate | CK | 21.9440^*^ | 0.000 | 17.2325 | 26.6556 |
|  | Sodium diacetate | 3.0185 | 0.090 | -0.3131 | 6.3501 |
|  | Sodium benzoate | -5.8258^*^ | 0.000 | -9.1574 | -2.4943 |
|  | Composite organic acid salt | -4.3471^*^ | 0.006 | -7.6786 | -1.0155 |
| Sodium benzoate | CK | 27.7699^*^ | 0.000 | 23.0583 | 32.4814 |
|  | Sodium diacetate | 8.8443^*^ | 0.000 | 5.5128 | 12.1759 |
|  | Sodium dehydroacetate | 5.8258^*^ | 0.000 | 2.4943 | 9.1574 |
|  | Composite organic acid salt | 1.4788 | 0.694 | -1.8528 | 4.8104 |
| Composite organic acid salt | CK | 26.2911^*^ | 0.000 | 21.5795 | 31.0027 |
|  | Sodium diacetate | 7.3655^*^ | 0.000 | 4.0340 | 10.6971 |
|  | Sodium dehydroacetate | 4.3471^*^ | 0.006 | 1.0155 | 7.6786 |
|  | Sodium benzoate | -1.4788 | 0.694 | -4.8104 | 1.8528 |
| *. The significance level of the mean difference is 0.05. | | | | | |

## CAT

| CK--1/4 MIC | | | | | |
| --- | --- | --- | --- | --- | --- |
| (A) | (B) | Mean difference (A-B) | Significance | 95% Confidence interval | |
|  |  |  |  | Lower bound | Upper bound |
| CK | Sodium diacetate | 108.74137 | 0.344 | -72.8497 | 290.3325 |
|  | Sodium dehydroacetate | 504.47347^*^ | 0.000 | 322.8824 | 686.0646 |
|  | Sodium benzoate | 15.29342 | 0.998 | -166.2977 | 196.8845 |
|  | Composite organic acid salt | 281.42190^*^ | 0.003 | 99.8308 | 463.0130 |
| Sodium diacetate | CK | -108.74137 | 0.344 | -290.3325 | 72.8497 |
|  | Sodium dehydroacetate | 395.73210^*^ | 0.000 | 214.1410 | 577.3232 |
|  | Sodium benzoate | -93.44795 | 0.478 | -275.0391 | 88.1432 |
|  | Composite organic acid salt | 172.68052 | 0.064 | -8.9106 | 354.2716 |
| Sodium dehydroacetate | CK | -504.47347^*^ | 0.000 | -686.0646 | -322.8824 |
|  | Sodium diacetate | -395.73210^*^ | 0.000 | -577.3232 | -214.1410 |
|  | Sodium benzoate | -489.18005^*^ | 0.000 | -670.7712 | -307.5889 |
|  | Composite organic acid salt | -223.05158^*^ | 0.016 | -404.6427 | -41.4605 |
| Sodium benzoate | CK | -15.29342 | 0.998 | -196.8845 | 166.2977 |
|  | Sodium diacetate | 93.44795 | 0.478 | -88.1432 | 275.0391 |
|  | Sodium dehydroacetate | 489.18005^*^ | 0.000 | 307.5889 | 670.7712 |
|  | Composite organic acid salt | 266.12847^*^ | 0.005 | 84.5374 | 447.7196 |
| Composite organic acid salt | CK | -281.42190^*^ | 0.003 | -463.0130 | -99.8308 |
|  | Sodium diacetate | -172.68052 | 0.064 | -354.2716 | 8.9106 |
|  | Sodium dehydroacetate | 223.05158^*^ | 0.016 | 41.4605 | 404.6427 |
|  | Sodium benzoate | -266.12847^*^ | 0.005 | -447.7196 | -84.5374 |
| *. The significance level of the mean difference is 0.05. | | | | | |

| CK—1/2 MIC | | | | | |
| --- | --- | --- | --- | --- | --- |
| (A) | (B) | Mean difference (A-B) | Significance | 95% Confidence interval | |
|  |  |  |  | Lower bound | Upper bound |
| CK | Sodium diacetate | 561.37235^*^ | 0.000 | 486.4644 | 636.2803 |
|  | Sodium dehydroacetate | 536.50654^*^ | 0.000 | 461.5986 | 611.4145 |
|  | Sodium benzoate | 257.28597^*^ | 0.000 | 182.3781 | 332.1939 |
|  | Composite organic acid salt | 443.79083^*^ | 0.000 | 368.8829 | 518.6987 |
| Sodium diacetate | CK | -561.37235^*^ | 0.000 | -636.2803 | -486.4644 |
|  | Sodium dehydroacetate | -24.86581 | 0.807 | -99.7737 | 50.0421 |
|  | Sodium benzoate | -304.08638^*^ | 0.000 | -378.9943 | -229.1785 |
|  | Composite organic acid salt | -117.58152^*^ | 0.003 | -192.4894 | -42.6736 |
| Sodium dehydroacetate | CK | -536.50654^*^ | 0.000 | -611.4145 | -461.5986 |
|  | Sodium diacetate | 24.86581 | 0.807 | -50.0421 | 99.7737 |
|  | Sodium benzoate | -279.22056^*^ | 0.000 | -354.1285 | -204.3126 |
|  | Composite organic acid salt | -92.71571^*^ | 0.015 | -167.6236 | -17.8078 |
| Sodium benzoate | CK | -257.28597^*^ | 0.000 | -332.1939 | -182.3781 |
|  | Sodium diacetate | 304.08638^*^ | 0.000 | 229.1785 | 378.9943 |
|  | Sodium dehydroacetate | 279.22056^*^ | 0.000 | 204.3126 | 354.1285 |
|  | Composite organic acid salt | 186.50485^*^ | 0.000 | 111.5969 | 261.4128 |
| Composite organic acid salt | CK | -443.79083^*^ | 0.000 | -518.6987 | -368.8829 |
|  | Sodium diacetate | 117.58152^*^ | 0.003 | 42.6736 | 192.4894 |
|  | Sodium dehydroacetate | 92.71571^*^ | 0.015 | 17.8078 | 167.6236 |
|  | Sodium benzoate | -186.50485^*^ | 0.000 | -261.4128 | -111.5969 |
| *. The significance level of the mean difference is 0.05. | | | | | |

| CK--MIC | | | | | |
| --- | --- | --- | --- | --- | --- |
| (A) | (B) | Mean difference (A-B) | Significance | 95% Confidence interval | |
|  |  |  |  | Lower bound | Upper bound |
| CK | Sodium diacetate | 616.22715^*^ | 0.000 | 544.9826 | 687.4717 |
|  | Sodium dehydroacetate | 652.96421^*^ | 0.000 | 581.7197 | 724.2088 |
|  | Sodium benzoate | 606.92723^*^ | 0.000 | 535.6827 | 678.1718 |
|  | Composite organic acid salt | 695.03372^*^ | 0.000 | 623.7892 | 766.2783 |
| Sodium diacetate | CK | -616.22715^*^ | 0.000 | -687.4717 | -544.9826 |
|  | Sodium dehydroacetate | 36.73706 | 0.477 | -34.5075 | 107.9816 |
|  | Sodium benzoate | -9.29992 | 0.992 | -80.5445 | 61.9446 |
|  | Composite organic acid salt | 78.80657^*^ | 0.029 | 7.5620 | 150.0511 |
| Sodium dehydroacetate | CK | -652.96421^*^ | 0.000 | -724.2088 | -581.7197 |
|  | Sodium diacetate | -36.73706 | 0.477 | -107.9816 | 34.5075 |
|  | Sodium benzoate | -46.03698 | 0.281 | -117.2815 | 25.2076 |
|  | Composite organic acid salt | 42.06951 | 0.356 | -29.1750 | 113.3141 |
| Sodium benzoate | CK | -606.92723^*^ | 0.000 | -678.1718 | -535.6827 |
|  | Sodium diacetate | 9.29992 | 0.992 | -61.9446 | 80.5445 |
|  | Sodium dehydroacetate | 46.03698 | 0.281 | -25.2076 | 117.2815 |
|  | Composite organic acid salt | 88.10649^*^ | 0.015 | 16.8619 | 159.3510 |
| Composite organic acid salt | CK | -695.03372^*^ | 0.000 | -766.2783 | -623.7892 |
|  | Sodium diacetate | -78.80657^*^ | 0.029 | -150.0511 | -7.5620 |
|  | Sodium dehydroacetate | -42.06951 | 0.356 | -113.3141 | 29.1750 |
|  | Sodium benzoate | -88.10649^*^ | 0.015 | -159.3510 | -16.8619 |
| *. The significance level of the mean difference is 0.05. | | | | | |

| Concentration | | | | | | |
| --- | --- | --- | --- | --- | --- | --- |
| (A) | (B) | | Mean difference (A-B) | Significance | 95% Confidence interval | |
|  |  |  |  |  | Lower bound | Upper bound |
| CK | | 1/4 MIC | 227.4825^*^ | 0.000 | 143.0964 | 311.8687 |
|  |  | 1/2 MIC | 449.7389^*^ | 0.000 | 365.3527 | 534.1251 |
|  |  | MIC | 642.7881^*^ | 0.000 | 558.4019 | 727.1743 |
| 1/4 MIC | | CK | -227.4825^*^ | 0.000 | -311.8687 | -143.0964 |
|  |  | 1/2 MIC | 222.2564^*^ | 0.000 | 168.8859 | 275.6269 |
|  |  | MIC | 415.3055^*^ | 0.000 | 361.9350 | 468.6760 |
| 1/2 MIC | | CK | -449.7389^*^ | 0.000 | -534.1251 | -365.3527 |
|  |  | 1/4 MIC | -222.2564^*^ | 0.000 | -275.6269 | -168.8859 |
|  |  | MIC | 193.0492^*^ | 0.000 | 139.6786 | 246.4197 |
| MIC | | CK | -642.7881^*^ | 0.000 | -727.1743 | -558.4019 |
|  |  | 1/4 MIC | -415.3055^*^ | 0.000 | -468.6760 | -361.9350 |
|  |  | 1/2 MIC | -193.0492^*^ | 0.000 | -246.4197 | -139.6786 |
| *. The significance level of the mean difference is 0.05. | | | | | | |

| Type | | | | | |
| --- | --- | --- | --- | --- | --- |
| (A) | (B) | Mean difference  (A-B) | Significance | 95% Confidence interval | |
|  |  |  |  | Lower bound | Upper bound |
| CK | Sodium diacetate | 428.7803^*^ | 0.000 | 335.7451 | 521.8155 |
|  | Sodium dehydroacetate | 564.6481^*^ | 0.000 | 471.6129 | 657.6833 |
|  | Sodium benzoate | 293.1689^*^ | 0.000 | 200.1337 | 386.2041 |
|  | Composite organic acid salt | 473.4155^*^ | 0.000 | 380.3803 | 566.4507 |
| Sodium diacetate | CK | -428.7803^*^ | 0.000 | -521.8155 | -335.7451 |
|  | Sodium dehydroacetate | 135.8678^*^ | 0.000 | 70.0820 | 201.6536 |
|  | Sodium benzoate | -135.6114^*^ | 0.000 | -201.3972 | -69.8256 |
|  | Composite organic acid salt | 44.6352 | 0.300 | -21.1506 | 110.4210 |
| Sodium dehydroacetate | CK | -564.6481^*^ | 0.000 | -657.6833 | -471.6129 |
|  | Sodium diacetate | -135.8678^*^ | 0.000 | -201.6536 | -70.0820 |
|  | Sodium benzoate | -271.4792^*^ | 0.000 | -337.2650 | -205.6934 |
|  | Composite organic acid salt | -91.2326^*^ | 0.003 | -157.0184 | -25.4468 |
| Sodium benzoate | CK | -293.1689^*^ | 0.000 | -386.2041 | -200.1337 |
|  | Sodium diacetate | 135.6114^*^ | 0.000 | 69.8256 | 201.3972 |
|  | Sodium dehydroacetate | 271.4792^*^ | 0.000 | 205.6934 | 337.2650 |
|  | Composite organic acid salt | 180.2466^*^ | 0.000 | 114.4608 | 246.0324 |
| Composite organic acid salt | CK | -473.4155^*^ | 0.000 | -566.4507 | -380.3803 |
|  | Sodium diacetate | -44.6352 | 0.300 | -110.4210 | 21.1506 |
|  | Sodium dehydroacetate | 91.2326^*^ | 0.003 | 25.4468 | 157.0184 |
|  | Sodium benzoate | -180.2466^*^ | 0.000 | -246.0324 | -114.4608 |
| *. The significance level of the mean difference is 0.05. | | | | | |

## GSH-Px

| CK--1/4 MIC | | | | | |
| --- | --- | --- | --- | --- | --- |
| (A) | (B) | Mean difference (A-B) | Significance | 95% Confidence interval | |
|  |  |  |  | Lower bound | Upper bound |
| CK | Sodium diacetate | -162.21936^*^ | 0.001 | -244.2715 | -80.1672 |
|  | Sodium dehydroacetate | -63.97490 | 0.151 | -146.0271 | 18.0772 |
|  | Sodium benzoate | -140.76314^*^ | 0.002 | -222.8153 | -58.7110 |
|  | Composite organic acid salt | -42.01017 | 0.483 | -124.0623 | 40.0420 |
| Sodium diacetate | CK | 162.21936^*^ | 0.001 | 80.1672 | 244.2715 |
|  | Sodium dehydroacetate | 98.24445^*^ | 0.018 | 16.1923 | 180.2966 |
|  | Sodium benzoate | 21.45621 | 0.905 | -60.5959 | 103.5084 |
|  | Composite organic acid salt | 120.20918^*^ | 0.005 | 38.1570 | 202.2613 |
| Sodium dehydroacetate | CK | 63.97490 | 0.151 | -18.0772 | 146.0271 |
|  | Sodium diacetate | -98.24445^*^ | 0.018 | -180.2966 | -16.1923 |
|  | Sodium benzoate | -76.78824 | 0.069 | -158.8404 | 5.2639 |
|  | Composite organic acid salt | 21.96473 | 0.898 | -60.0874 | 104.0169 |
| Sodium benzoate | CK | 140.76314^*^ | 0.002 | 58.7110 | 222.8153 |
|  | Sodium diacetate | -21.45621 | 0.905 | -103.5084 | 60.5959 |
|  | Sodium dehydroacetate | 76.78824 | 0.069 | -5.2639 | 158.8404 |
|  | Composite organic acid salt | 98.75297^*^ | 0.018 | 16.7008 | 180.8051 |
| Composite organic acid salt | CK | 42.01017 | 0.483 | -40.0420 | 124.0623 |
|  | Sodium diacetate | -120.20918^*^ | 0.005 | -202.2613 | -38.1570 |
|  | Sodium dehydroacetate | -21.96473 | 0.898 | -104.0169 | 60.0874 |
|  | Sodium benzoate | -98.75297^*^ | 0.018 | -180.8051 | -16.7008 |
| *. The significance level of the mean difference is 0.05. | | | | | |

| CK—1/2 MIC | | | | | |
| --- | --- | --- | --- | --- | --- |
| (A) | (B) | Mean difference (A-B) | Significance | 95% Confidence interval | |
|  |  |  |  | Lower bound | Upper bound |
| CK | Sodium diacetate | -194.79956^*^ | 0.001 | -306.3742 | -83.2249 |
|  | Sodium dehydroacetate | -74.56183 | 0.255 | -186.1365 | 37.0128 |
|  | Sodium benzoate | -199.06051^*^ | 0.001 | -310.6351 | -87.4859 |
|  | Composite organic acid salt | -125.96394^*^ | 0.026 | -237.5386 | -14.3893 |
| Sodium diacetate | CK | 194.79956^*^ | 0.001 | 83.2249 | 306.3742 |
|  | Sodium dehydroacetate | 120.23773^*^ | 0.034 | 8.6631 | 231.8124 |
|  | Sodium benzoate | -4.26095 | 1.000 | -115.8356 | 107.3137 |
|  | Composite organic acid salt | 68.83562 | 0.319 | -42.7390 | 180.4103 |
| Sodium dehydroacetate | CK | 74.56183 | 0.255 | -37.0128 | 186.1365 |
|  | Sodium diacetate | -120.23773^*^ | 0.034 | -231.8124 | -8.6631 |
|  | Sodium benzoate | -124.49868^*^ | 0.028 | -236.0733 | -12.9240 |
|  | Composite organic acid salt | -51.40212 | 0.575 | -162.9768 | 60.1725 |
| Sodium benzoate | CK | 199.06051^*^ | 0.001 | 87.4859 | 310.6351 |
|  | Sodium diacetate | 4.26095 | 1.000 | -107.3137 | 115.8356 |
|  | Sodium dehydroacetate | 124.49868^*^ | 0.028 | 12.9240 | 236.0733 |
|  | Composite organic acid salt | 73.09656 | 0.270 | -38.4781 | 184.6712 |
| Composite organic acid salt | CK | 125.96394^*^ | 0.026 | 14.3893 | 237.5386 |
|  | Sodium diacetate | -68.83562 | 0.319 | -180.4103 | 42.7390 |
|  | Sodium dehydroacetate | 51.40212 | 0.575 | -60.1725 | 162.9768 |
|  | Sodium benzoate | -73.09656 | 0.270 | -184.6712 | 38.4781 |
| *. The significance level of the mean difference is 0.05. | | | | | |

| CK--MIC | | | | | |
| --- | --- | --- | --- | --- | --- |
| (A) | (B) | Mean difference (A-B) | Significance | 95% Confidence interval | |
|  |  |  |  | Lower bound | Upper bound |
| CK | Sodium diacetate | -416.64488^*^ | 0.000 | -507.5057 | -325.7840 |
|  | Sodium dehydroacetate | -89.21066 | 0.055 | -180.0715 | 1.6502 |
|  | Sodium benzoate | -388.36332^*^ | 0.000 | -479.2242 | -297.5025 |
|  | Composite organic acid salt | -523.69803^*^ | 0.000 | -614.5589 | -432.8372 |
| Sodium diacetate | CK | 416.64488^*^ | 0.000 | 325.7840 | 507.5057 |
|  | Sodium dehydroacetate | 327.43421^*^ | 0.000 | 236.5734 | 418.2951 |
|  | Sodium benzoate | 28.28155 | 0.839 | -62.5793 | 119.1424 |
|  | Composite organic acid salt | -107.05315^*^ | 0.020 | -197.9140 | -16.1923 |
| Sodium dehydroacetate | CK | 89.21066 | 0.055 | -1.6502 | 180.0715 |
|  | Sodium diacetate | -327.43421^*^ | 0.000 | -418.2951 | -236.5734 |
|  | Sodium benzoate | -299.15266^*^ | 0.000 | -390.0135 | -208.2918 |
|  | Composite organic acid salt | -434.48736^*^ | 0.000 | -525.3482 | -343.6265 |
| Sodium benzoate | CK | 388.36332^*^ | 0.000 | 297.5025 | 479.2242 |
|  | Sodium diacetate | -28.28155 | 0.839 | -119.1424 | 62.5793 |
|  | Sodium dehydroacetate | 299.15266^*^ | 0.000 | 208.2918 | 390.0135 |
|  | Composite organic acid salt | -135.33470^*^ | 0.004 | -226.1955 | -44.4739 |
| Composite organic acid salt | CK | 523.69803^*^ | 0.000 | 432.8372 | 614.5589 |
|  | Sodium diacetate | 107.05315^*^ | 0.020 | 16.1923 | 197.9140 |
|  | Sodium dehydroacetate | 434.48736^*^ | 0.000 | 343.6265 | 525.3482 |
|  | Sodium benzoate | 135.33470^*^ | 0.004 | 44.4739 | 226.1955 |
| *. The significance level of the mean difference is 0.05. | | | | | |

| Concentration | | | | | | |
| --- | --- | --- | --- | --- | --- | --- |
| (A) | (B) | | Mean difference  (A-B) | Significance | 95% Confidence interval | |
|  |  |  |  |  | Lower bound | Upper bound |
| CK | | 1/4 MIC | -102.2419^*^ | 0.001 | -167.2225 | -37.2613 |
|  |  | 1/2 MIC | -148.5965^*^ | 0.000 | -213.5771 | -83.6158 |
|  |  | MIC | -354.4792^*^ | 0.000 | -419.4599 | -289.4986 |
| 1/4 MIC | | CK | 102.2419^*^ | 0.001 | 37.2613 | 167.2225 |
|  |  | 1/2 MIC | -46.3546^*^ | 0.023 | -87.4519 | -5.2572 |
|  |  | MIC | -252.2373^*^ | 0.000 | -293.3347 | -211.1400 |
| 1/2 MIC | | CK | 148.5965^*^ | 0.000 | 83.6158 | 213.5771 |
|  |  | 1/4 MIC | 46.3546^*^ | 0.023 | 5.2572 | 87.4519 |
|  |  | MIC | -205.8828^*^ | 0.000 | -246.9801 | -164.7854 |
| MIC | | CK | 354.4792^*^ | 0.000 | 289.4986 | 419.4599 |
|  |  | 1/4 MIC | 252.2373^*^ | 0.000 | 211.1400 | 293.3347 |
|  |  | 1/2 MIC | 205.8828^*^ | 0.000 | 164.7854 | 246.9801 |
| *. The significance level of the mean difference is 0.05. | | | | | | |

| Type | | | | | |
| --- | --- | --- | --- | --- | --- |
| (A) | (B) | Mean difference  (A-B) | Significance | 95% Confidence interval | |
|  |  |  |  | Lower bound | Upper bound |
| CK | Sodium diacetate | -257.8879^*^ | 0.000 | -329.5286 | -186.2472 |
|  | Sodium dehydroacetate | -75.9158^*^ | 0.034 | -147.5565 | -4.2751 |
|  | Sodium benzoate | -242.7290^*^ | 0.000 | -314.3697 | -171.0883 |
|  | Composite organic acid salt | -230.5574^*^ | 0.000 | -302.1981 | -158.9167 |
| Sodium diacetate | CK | 257.8879^*^ | 0.000 | 186.2472 | 329.5286 |
|  | Sodium dehydroacetate | 181.9721^*^ | 0.000 | 131.3145 | 232.6298 |
|  | Sodium benzoate | 15.1589 | 0.903 | -35.4987 | 65.8166 |
|  | Composite organic acid salt | 27.3306 | 0.523 | -23.3271 | 77.9882 |
| Sodium dehydroacetate | CK | 75.9158^*^ | 0.034 | 4.2751 | 147.5565 |
|  | Sodium diacetate | -181.9721^*^ | 0.000 | -232.6298 | -131.3145 |
|  | Sodium benzoate | -166.8132^*^ | 0.000 | -217.4708 | -116.1556 |
|  | Composite organic acid salt | -154.6416^*^ | 0.000 | -205.2992 | -103.9840 |
| Sodium benzoate | CK | 242.7290^*^ | 0.000 | 171.0883 | 314.3697 |
|  | Sodium diacetate | -15.1589 | 0.903 | -65.8166 | 35.4987 |
|  | Sodium dehydroacetate | 166.8132^*^ | 0.000 | 116.1556 | 217.4708 |
|  | Composite organic acid salt | 12.1716 | 0.954 | -38.4860 | 62.8292 |
| Composite organic acid salt | CK | 230.5574^*^ | 0.000 | 158.9167 | 302.1981 |
|  | Sodium diacetate | -27.3306 | 0.523 | -77.9882 | 23.3271 |
|  | Sodium dehydroacetate | 154.6416^*^ | 0.000 | 103.9840 | 205.2992 |
|  | Sodium benzoate | -12.1716 | 0.954 | -62.8292 | 38.4860 |
| *. The significance level of the mean difference is 0.05. | | | | | |

## MDA

| CK--1/4 MIC | | | | | |
| --- | --- | --- | --- | --- | --- |
| (A) | (B) | Mean difference  (A-B) | Significance | 95% Confidence interval | |
|  |  |  |  | Lower bound | Upper bound |
| CK | Sodium diacetate | -5.41773^*^ | 0.001 | -8.2237 | -2.6118 |
|  | Sodium dehydroacetate | -3.84076^*^ | 0.008 | -6.6467 | -1.0348 |
|  | Sodium benzoate | -3.27281^*^ | 0.021 | -6.0788 | -0.4669 |
|  | Composite organic acid salt | -5.19255^*^ | 0.001 | -7.9985 | -2.3866 |
| Sodium diacetate | CK | 5.41773^*^ | 0.001 | 2.6118 | 8.2237 |
|  | Sodium dehydroacetate | 1.57696 | 0.400 | -1.2290 | 4.3829 |
|  | Sodium benzoate | 2.14491 | 0.163 | -0.6610 | 4.9509 |
|  | Composite organic acid salt | 0.22517 | 0.999 | -2.5808 | 3.0311 |
| Sodium dehydroacetate | CK | 3.84076^*^ | 0.008 | 1.0348 | 6.6467 |
|  | Sodium diacetate | -1.57696 | 0.400 | -4.3829 | 1.2290 |
|  | Sodium benzoate | 0.56795 | 0.959 | -2.2380 | 3.3739 |
|  | Composite organic acid salt | -1.35179 | 0.537 | -4.1577 | 1.4542 |
| Sodium benzoate | CK | 3.27281^*^ | 0.021 | 0.4669 | 6.0788 |
|  | Sodium diacetate | -2.14491 | 0.163 | -4.9509 | 0.6610 |
|  | Sodium dehydroacetate | -0.56795 | 0.959 | -3.3739 | 2.2380 |
|  | Composite organic acid salt | -1.91974 | 0.237 | -4.7257 | 0.8862 |
| Composite organic acid salt | CK | 5.19255^*^ | 0.001 | 2.3866 | 7.9985 |
|  | Sodium diacetate | -0.22517 | 0.999 | -3.0311 | 2.5808 |
|  | Sodium dehydroacetate | 1.35179 | 0.537 | -1.4542 | 4.1577 |
|  | Sodium benzoate | 1.91974 | 0.237 | -0.8862 | 4.7257 |
| *. The significance level of the mean difference is 0.05. | | | | | |

| CK—1/2 MIC | | | | | |
| --- | --- | --- | --- | --- | --- |
| (A) | (B) | Mean difference  (A-B) | Significance | 95% Confidence interval | |
|  |  |  |  | Lower bound | Upper bound |
| CK | Sodium diacetate | -5.72962^*^ | 0.015 | -10.3479 | -1.1113 |
|  | Sodium dehydroacetate | -5.11883^*^ | 0.029 | -9.7371 | -0.5005 |
|  | Sodium benzoate | -4.88655^*^ | 0.037 | -9.5049 | -0.2682 |
|  | Composite organic acid salt | -6.14377^*^ | 0.009 | -10.7621 | -1.5255 |
| Sodium diacetate | CK | 5.72962^*^ | 0.015 | 1.1113 | 10.3479 |
|  | Sodium dehydroacetate | 0.61079 | 0.991 | -4.0075 | 5.2291 |
|  | Sodium benzoate | 0.84306 | 0.972 | -3.7752 | 5.4614 |
|  | Composite organic acid salt | -0.41416 | 0.998 | -5.0325 | 4.2042 |
| Sodium dehydroacetate | CK | 5.11883^*^ | 0.029 | 0.5005 | 9.7371 |
|  | Sodium diacetate | -0.61079 | 0.991 | -5.2291 | 4.0075 |
|  | Sodium benzoate | 0.23228 | 1.000 | -4.3860 | 4.8506 |
|  | Composite organic acid salt | -1.02494 | 0.944 | -5.6433 | 3.5934 |
| Sodium benzoate | CK | 4.88655^*^ | 0.037 | 0.2682 | 9.5049 |
|  | Sodium diacetate | -0.84306 | 0.972 | -5.4614 | 3.7752 |
|  | Sodium dehydroacetate | -0.23228 | 1.000 | -4.8506 | 4.3860 |
|  | Composite organic acid salt | -1.25722 | 0.892 | -5.8755 | 3.3611 |
| Composite organic acid salt | CK | 6.14377^*^ | 0.009 | 1.5255 | 10.7621 |
|  | Sodium diacetate | 0.41416 | 0.998 | -4.2042 | 5.0325 |
|  | Sodium dehydroacetate | 1.02494 | 0.944 | -3.5934 | 5.6433 |
|  | Sodium benzoate | 1.25722 | 0.892 | -3.3611 | 5.8755 |
| *. The significance level of the mean difference is 0.05. | | | | | |

| CK--MIC | | | | | |
| --- | --- | --- | --- | --- | --- |
| (A) | (B) | Mean difference  (A-B) | Significance | 95% Confidence interval | |
|  |  |  |  | Lower bound | Upper bound |
| CK | Sodium diacetate | -7.84996^*^ | 0.000 | -11.6966 | -4.0033 |
|  | Sodium dehydroacetate | -7.26323^*^ | 0.001 | -11.1099 | -3.4165 |
|  | Sodium benzoate | -7.83147^*^ | 0.000 | -11.6782 | -3.9848 |
|  | Composite organic acid salt | -8.73911^*^ | 0.000 | -12.5858 | -4.8924 |
| Sodium diacetate | CK | 7.84996^*^ | 0.000 | 4.0033 | 11.6966 |
|  | Sodium dehydroacetate | 0.58673 | 0.985 | -3.2600 | 4.4334 |
|  | Sodium benzoate | 0.01849 | 1.000 | -3.8282 | 3.8652 |
|  | Composite organic acid salt | -0.88915 | 0.936 | -4.7358 | 2.9575 |
| Sodium dehydroacetate | CK | 7.26323^*^ | 0.001 | 3.4165 | 11.1099 |
|  | Sodium diacetate | -0.58673 | 0.985 | -4.4334 | 3.2600 |
|  | Sodium benzoate | -0.56824 | 0.987 | -4.4149 | 3.2784 |
|  | Composite organic acid salt | -1.47588 | 0.718 | -5.3226 | 2.3708 |
| Sodium benzoate | CK | 7.83147^*^ | 0.000 | 3.9848 | 11.6782 |
|  | Sodium diacetate | -0.01849 | 1.000 | -3.8652 | 3.8282 |
|  | Sodium dehydroacetate | 0.56824 | 0.987 | -3.2784 | 4.4149 |
|  | Composite organic acid salt | -0.90764 | 0.932 | -4.7543 | 2.9391 |
| Composite organic acid salt | CK | 8.73911^*^ | 0.000 | 4.8924 | 12.5858 |
|  | Sodium diacetate | 0.88915 | 0.936 | -2.9575 | 4.7358 |
|  | Sodium dehydroacetate | 1.47588 | 0.718 | -2.3708 | 5.3226 |
|  | Sodium benzoate | 0.90764 | 0.932 | -2.9391 | 4.7543 |
| *. The significance level of the mean difference is 0.05. | | | | | |

| Concentration | | | | | |
| --- | --- | --- | --- | --- | --- |
| (A) | (B) | Mean difference  (A-B) | Significance | 95% Confidence interval | |
|  |  |  |  | Lower bound | Upper bound |
| CK | 1/4 MIC | -4.4310^*^ | 0.001 | -7.0815 | -1.7804 |
|  | 1/2 MIC | -5.4697^*^ | 0.000 | -8.1203 | -2.8191 |
|  | MIC | -7.9209^*^ | 0.000 | -10.5715 | -5.2704 |
| 1/4 MIC | CK | 4.4310^*^ | 0.001 | 1.7804 | 7.0815 |
|  | 1/2 MIC | -1.0387 | 0.344 | -2.7151 | 0.6376 |
|  | MIC | -3.4900^*^ | 0.000 | -5.1664 | -1.8136 |
| 1/2 MIC | CK | 5.4697^*^ | 0.000 | 2.8191 | 8.1203 |
|  | 1/4 MIC | 1.0387 | 0.344 | -0.6376 | 2.7151 |
|  | MIC | -2.4512^*^ | 0.002 | -4.1276 | -0.7749 |
| MIC | CK | 7.9209^*^ | 0.000 | 5.2704 | 10.5715 |
|  | 1/4 MIC | 3.4900^*^ | 0.000 | 1.8136 | 5.1664 |
|  | 1/2 MIC | 2.4512^*^ | 0.002 | 0.7749 | 4.1276 |
| *. The significance level of the mean difference is 0.05. | | | | | |

| Type | | | | | |
| --- | --- | --- | --- | --- | --- |
| (A) | (B) | Mean difference  (A-B) | Significance | 95% Confidence interval | |
|  |  |  |  | Lower bound | Upper bound |
| CK | Sodium diacetate | -6.3324^*^ | 0.000 | -9.2547 | -3.4102 |
|  | Sodium dehydroacetate | -5.4076^*^ | 0.000 | -8.3299 | -2.4854 |
|  | Sodium benzoate | -5.3303^*^ | 0.000 | -8.2525 | -2.4080 |
|  | Composite organic acid salt | -6.6918^*^ | 0.000 | -9.6141 | -3.7696 |
| Sodium diacetate | CK | 6.3324^*^ | 0.000 | 3.4102 | 9.2547 |
|  | Sodium dehydroacetate | 0.9248 | 0.687 | -1.1415 | 2.9912 |
|  | Sodium benzoate | 1.0022 | 0.621 | -1.0642 | 3.0685 |
|  | Composite organic acid salt | -0.3594 | 0.986 | -2.4257 | 1.7070 |
| Sodium dehydroacetate | CK | 5.4076^*^ | 0.000 | 2.4854 | 8.3299 |
|  | Sodium diacetate | -0.9248 | 0.687 | -2.9912 | 1.1415 |
|  | Sodium benzoate | 0.0773 | 1.000 | -1.9890 | 2.1437 |
|  | Composite organic acid salt | -1.2842 | 0.384 | -3.3505 | 0.7821 |
| Sodium benzoate | CK | 5.3303^*^ | 0.000 | 2.4080 | 8.2525 |
|  | Sodium diacetate | -1.0022 | 0.621 | -3.0685 | 1.0642 |
|  | Sodium dehydroacetate | -0.0773 | 1.000 | -2.1437 | 1.9890 |
|  | Composite organic acid salt | -1.3615 | 0.328 | -3.4279 | 0.7048 |
| Composite organic acid salt | CK | 6.6918^*^ | 0.000 | 3.7696 | 9.6141 |
|  | Sodium diacetate | 0.3594 | 0.986 | -1.7070 | 2.4257 |
|  | Sodium dehydroacetate | 1.2842 | 0.384 | -0.7821 | 3.3505 |
|  | Sodium benzoate | 1.3615 | 0.328 | -0.7048 | 3.4279 |
| *. The significance level of the mean difference is 0.05. | | | | | |

## Key genes

| gene | (A) | (B) | Mean difference  (A-B) | Significance | 95% Confidence interval | |
| --- | --- | --- | --- | --- | --- | --- |
|  |  |  |  |  | Lower bound | Upper bound |
| AFLR | CK | 1/2 MIC | 0.08960 | 0.341 | -0.0893 | 0.2685 |
|  |  | MIC | .87442^*^ | 0.000 | 0.6955 | 1.0533 |
|  | 1/2 MIC | CK | -0.08960 | 0.341 | -0.2685 | 0.0893 |
|  |  | MIC | .78481^*^ | 0.000 | 0.6059 | 0.9637 |
|  | MIC | CK | -.87442^*^ | 0.000 | -1.0533 | -0.6955 |
|  |  | 1/2 MIC | -.78481^*^ | 0.000 | -0.9637 | -0.6059 |
| AFLS | CK | 1/2 MIC | 0.16474 | 0.096 | -0.0335 | 0.3630 |
|  |  | MIC | .90017^*^ | 0.000 | 0.7020 | 1.0984 |
|  | 1/2 MIC | CK | -0.16474 | 0.096 | -0.3630 | 0.0335 |
|  |  | MIC | .73543^*^ | 0.000 | 0.5372 | 0.9336 |
|  | MIC | CK | -.90017^*^ | 0.000 | -1.0984 | -0.7020 |
|  |  | 1/2 MIC | -.73543^*^ | 0.000 | -0.9336 | -0.5372 |
| AFLD | CK | 1/2 MIC | -0.25957 | 0.122 | -0.5971 | 0.0780 |
|  |  | MIC | .83038^*^ | 0.001 | 0.4928 | 1.1679 |
|  | 1/2 MIC | CK | 0.25957 | 0.122 | -0.0780 | 0.5971 |
|  |  | MIC | 1.08994^*^ | 0.000 | 0.7524 | 1.4275 |
|  | MIC | CK | -.83038^*^ | 0.001 | -1.1679 | -0.4928 |
|  |  | 1/2 MIC | -1.08994^*^ | 0.000 | -1.4275 | -0.7524 |
| AFLM | CK | 1/2 MIC | -0.21236 | 0.182 | -0.5310 | 0.1063 |
|  |  | MIC | .86816^*^ | 0.000 | 0.5495 | 1.1868 |
|  | 1/2 MIC | CK | 0.21236 | 0.182 | -0.1063 | 0.5310 |
|  |  | MIC | 1.08052^*^ | 0.000 | 0.7619 | 1.3991 |
|  | MIC | CK | -.86816^*^ | 0.000 | -1.1868 | -0.5495 |
|  |  | 1/2 MIC | -1.08052^*^ | 0.000 | -1.3991 | -0.7619 |
| AFLT | CK | 1/2 MIC | .25488^*^ | 0.013 | 0.0686 | 0.4411 |
|  |  | MIC | .88971^*^ | 0.000 | 0.7034 | 1.0760 |
|  | 1/2 MIC | CK | -.25488^*^ | 0.013 | -0.4411 | -0.0686 |
|  |  | MIC | .63483^*^ | 0.000 | 0.4486 | 0.8211 |
|  | MIC | CK | -.88971^*^ | 0.000 | -1.0760 | -0.7034 |
|  |  | 1/2 MIC | -.63483^*^ | 0.000 | -0.8211 | -0.4486 |
| AFLC | CK | 1/2 MIC | .27519^*^ | 0.028 | 0.0371 | 0.5133 |
|  |  | MIC | .88477^*^ | 0.000 | 0.6467 | 1.1229 |
|  | 1/2 MIC | CK | -.27519^*^ | 0.028 | -0.5133 | -0.0371 |
|  |  | MIC | .60958^*^ | 0.001 | 0.3715 | 0.8477 |
|  | MIC | CK | -.88477^*^ | 0.000 | -1.1229 | -0.6467 |
|  |  | 1/2 MIC | -.60958^*^ | 0.001 | -0.8477 | -0.3715 |
| *. The significance level of the mean difference is 0.05. | | | | | | |

# Outlier Detection Utilizing the Boxplot Method

## Fig. 1 Outlier analysis of mycelial dry weight

Note: The horizontal axis in Fig. 1 represents the following conditions: 1 and 2 are the control groups at 24h and 48h without any treatment. 3, 4, and 5 correspond to treatments with 1/4 MIC, 1/2 MIC, and MIC of Sodium Diacetate, respectively. 6, 7, and 8 represent treatments with 1/4 MIC, 1/2 MIC, and MIC of Sodium Dehydroacetate, respectively. 9, 10, and 11 denote treatments with 1/4 MIC, 1/2 MIC, and MIC of Sodium Benzoate, respectively. 12, 13, and 14 indicate treatments with 1/4 MIC, 1/2 MIC, and MIC of the Composite Organic Acid Salt, respectively.

A) CK B) 1/4 MIC

C) 1/2 MIC D) MIC

## Fig. 2 Outlier analysis of relative conductivity

A) CK B) 1/4 MIC

C) 1/2 MIC D) MIC

## Fig. 3 Outlier analysis of pH

## Fig. 4 Outlier analysis of total lipids

Note: The horizontal axis in Fig. 4 represents the following conditions: 1 is the control group; 2, 3, and 4 correspond to treatments with 1/4 MIC, 1/2 MIC, and MIC of Sodium Diacetate, respectively; 5, 6, and 7 represent treatments with 1/4 MIC, 1/2 MIC, and MIC of Sodium Dehydroacetate, respectively; 8, 9, and 10 denote treatments with 1/4 MIC, 1/2 MIC, and MIC of Sodium Benzoate, respectively; 11, 12, and 13 indicate treatments with 1/4 MIC, 1/2 MIC, and MIC of the Composite Organic Acid Salt, respectively.

## Fig. 5 Outlier analysis of ergosterol

Note: The horizontal axis in Fig. 5 represents the following conditions: 1 is the control group; 2, 3, and 4 are treatments with 1/4 MIC, 1/2 MIC, and MIC of Sodium Diacetate, respectively; 5, 6, and 7 are treatments with 1/4 MIC, 1/2 MIC, and MIC of Sodium Dehydroacetate, respectively; 8, 9, and 10 are treatments with 1/4 MIC, 1/2 MIC, and MIC of Sodium Benzoate, respectively; 11, 12, and 13 are treatments with 1/4 MIC, 1/2 MIC, and MIC of the Composite Organic Acid Salt, respectively.

## Fig. 6 Outlier analysis of trehalose

Note: The horizontal axis in Fig. 6 represents the following conditions: 1 is the control group; 2, 3, and 4 are treatments with 1/4 MIC, 1/2 MIC, and MIC of Sodium Diacetate, respectively; 5, 6, and 7 are treatments with 1/4 MIC, 1/2 MIC, and MIC of Sodium Dehydroacetate, respectively; 8, 9, and 10 are treatments with 1/4 MIC, 1/2 MIC, and MIC of Sodium Benzoate, respectively; 11, 12, and 13 are treatments with 1/4 MIC, 1/2 MIC, and MIC of the Composite Organic Acid Salt, respectively.

## Fig. 7 Outlier analysis of SOD

Note: The horizontal axis in Fig. 7 represents the following conditions: 1 is the control group; 2, 3, and 4 are treatments with 1/4 MIC, 1/2 MIC, and MIC of Sodium Diacetate, respectively; 5, 6, and 7 are treatments with 1/4 MIC, 1/2 MIC, and MIC of Sodium Dehydroacetate, respectively; 8, 9, and 10 are treatments with 1/4 MIC, 1/2 MIC, and MIC of Sodium Benzoate, respectively; 11, 12, and 13 are treatments with 1/4 MIC, 1/2 MIC, and MIC of the Composite Organic Acid Salt, respectively.

## Fig. 8 Outlier analysis of CAT

Note: The horizontal axis in Fig. 8 represents the following conditions: 1 is the control group; 2, 3, and 4 are treatments with 1/4 MIC, 1/2 MIC, and MIC of Sodium Diacetate, respectively; 5, 6, and 7 are treatments with 1/4 MIC, 1/2 MIC, and MIC of Sodium Dehydroacetate, respectively; 8, 9, and 10 are treatments with 1/4 MIC, 1/2 MIC, and MIC of Sodium Benzoate, respectively; 11, 12, and 13 are treatments with 1/4 MIC, 1/2 MIC, and MIC of the Composite Organic Acid Salt, respectively.

## Fig. 9 Outlier analysis of GSH-Px

Note: The horizontal axis in Fig. 9 represents the following conditions: 1 is the control group; 2, 3, and 4 are treatments with 1/4 MIC, 1/2 MIC, and MIC of Sodium Diacetate, respectively; 5, 6, and 7 are treatments with 1/4 MIC, 1/2 MIC, and MIC of Sodium Dehydroacetate, respectively; 8, 9, and 10 are treatments with 1/4 MIC, 1/2 MIC, and MIC of Sodium Benzoate, respectively; 11, 12, and 13 are treatments with 1/4 MIC, 1/2 MIC, and MIC of the Composite Organic Acid Salt, respectively.

## Fig. 10 Outlier analysis of MDA

Note: The horizontal axis in Fig. 10 represents the following conditions: 1 is the control group; 2, 3, and 4 are treatments with 1/4 MIC, 1/2 MIC, and MIC of Sodium Diacetate, respectively; 5, 6, and 7 are treatments with 1/4 MIC, 1/2 MIC, and MIC of Sodium Dehydroacetate, respectively; 8, 9, and 10 are treatments with 1/4 MIC, 1/2 MIC, and MIC of Sodium Benzoate, respectively; 11, 12, and 13 are treatments with 1/4 MIC, 1/2 MIC, and MIC of the Composite Organic Acid Salt, respectively.

## Fig. 11 Outlier analysis of key genes

Note: The horizontal axis in Fig. 11 represents the following conditions: 1, 2, and 3 correspond to the relative expression levels of aflR after CK, MIC, and 1/2 MIC treatments, respectively; 4, 5, and 6 correspond to the relative expression levels of aflS after CK, MIC, and 1/2 MIC treatments, respectively; 7, 8, and 9 correspond to the relative expression levels of aflD after CK, MIC, and 1/2 MIC treatments, respectively; 10, 11, and 12 correspond to the relative expression levels of aflM after CK, MIC, and 1/2 MIC treatments, respectively; 13, 14, and 15 correspond to the relative expression levels of aflC after CK, MIC, and 1/2 MIC treatments, respectively; 16, 17, and 18 correspond to the relative expression levels of aflT after CK, MIC, and 1/2 MIC treatments, respectively.

# Outlier Detection Based on Absolute Distance

The core principle of this method is that if a data point is an outlier, its average distance to all other data points should be significantly larger than that of normal points. The entire process consists of two main stages:

1. Calculation of Outlier Score for Each Point: The mean absolute distance from each point to all other points in the dataset is used as an indicator to measure the degree of its abnormality.
2. Identification of Outliers Based on Scores: The statistical Interquartile Range method is utilized to identify which of these 'outlier scores' are excessively high, thereby determining the corresponding original data points as outliers.

The detailed implementation steps are as follows:

- Calculation of Mean Distance.

For each data point *x_i_* in the dataset, the arithmetic mean *d_i_* of the absolute distances between itself and all other points *x_j_* ​is calculated, as shown in Eq. (1):

$d_{i}=\frac{1}{N-1}\sum_{j=1,j\neq i}^{N} \left|x_{i}-x_{j}\right|$ (1)

where *N* represents the total number of data points.

- Determination of Outlier Thresholds.

Based on the previous step, a set of distance values $D=\left\{d_{1},d_{2},...,d_{N}\right\}$ is obtained. Subsequently, the *IQR* rule is applied to automatically determine the outlier thresholds. First, the upper (*Q*3) and lower (*Q*1) quartiles of the distance array Dare computed. Then, the interquartile range is calculated using Eq. (2). Finally, the upper and lower thresholds for outlier detection are established using Eq. (3) and (4).

$IQR=Q3-Q1$ (2)

$T_{\text{lower}}=Q1-1.5\times IQR$ (3)

$T_{\text{upper}}=Q3+1.5\times IQR$ (4)

Where $T_{\text{upper}}$ and $T_{\text{lower}}$ represent the upper and lower thresholds for outlier identification, respectively.

- Identification of Outliers.

The array *D* is traversed, and any data point whose distance value d satisfies the condition in Eq. (5) is flagged as an outlier.

$\left\{\begin{matrix} d_{i}>T_{\text{upper}} \\ d_{i}<T_{\text{lower}} \end{matrix}\right.$ (5)

## Mycelium Dry Weight

| Group | | Dry weight of mycelium | Absolute distance | *Q*1 | *Q*3 | *IQR* | Lower threshold | Upper threshold | Number of outliers |
| --- | --- | --- | --- | --- | --- | --- | --- | --- | --- |
| CK | 24h | 0.27 | 0.03 | 0.025 | 0.03 | 0.005 | 0.0175 | 0.0375 | 0 |
|  |  | 0.31 | 0.03 |  |  |  |  |  |  |
|  |  | 0.29 | 0.02 |  |  |  |  |  |  |
|  | 48h | 1.07 | 0.1075 | 0.12625 | 0.16125 | 0.035 | 0.07375 | 0.21375 | 0 |
|  |  | 1.21 | 0.1775 |  |  |  |  |  |  |
|  |  | 0.995 | 0.145 |  |  |  |  |  |  |
| Sodium diacetate | 1/4  MIC | 0.235 | 0.015 | 0.0125 | 0.015 | 0.0025 | 0.00875 | 0.01875 | 0 |
|  |  | 0.255 | 0.015 |  |  |  |  |  |  |
|  |  | 0.245 | 0.01 |  |  |  |  |  |  |
|  | 1/2  MIC | 0.24 | 0.0025 | 0.0025 | 0.00375 | 0.00125 | 0.000625 | 0.005625 | 0 |
|  |  | 0.24 | 0.0025 |  |  |  |  |  |  |
|  |  | 0.245 | 0.005 |  |  |  |  |  |  |
|  | MIC | 0.18 | 0.02 | 0.01875 | 0.02625 | 0.0075 | 0.0075 | 0.0375 | 0 |
|  |  | 0.215 | 0.0325 |  |  |  |  |  |  |
|  |  | 0.185 | 0.0175 |  |  |  |  |  |  |
| Sodium dehydroa-cetate | 1/4  MIC | 0.465 | 0.0175 | 0.01625 | 0.0225 | 0.00625 | 0.006875 | 0.031875 | 0 |
|  |  | 0.435 | 0.0275 |  |  |  |  |  |  |
|  |  | 0.46 | 0.015 |  |  |  |  |  |  |
|  | 1/2  MIC | 0.29 | 0.025 | 0.02125 | 0.02625 | 0.005 | 0.01375 | 0.03375 | 0 |
|  |  | 0.325 | 0.0275 |  |  |  |  |  |  |
|  |  | 0.305 | 0.0175 |  |  |  |  |  |  |
|  | MIC | 0.24 | 0.0075 | 0.00625 | 0.0075 | 0.00125 | 0.004375 | 0.009375 | 0 |
|  |  | 0.245 | 0.005 |  |  |  |  |  |  |
|  |  | 0.25 | 0.0075 |  |  |  |  |  |  |
| Sodium benzoate | 1/4  MIC | 0.355 | 0.01 | 0.0125 | 0.015 | 0.0025 | 0.00875 | 0.01875 | 0 |
|  |  | 0.345 | 0.015 |  |  |  |  |  |  |
|  |  | 0.365 | 0.015 |  |  |  |  |  |  |
|  | 1/2  MIC | 0.315 | 0.0025 | 0.0025 | 0.00375 | 0.00125 | 0.000625 | 0.005625 | 0 |
|  |  | 0.32 | 0.005 |  |  |  |  |  |  |
|  |  | 0.315 | 0.0025 |  |  |  |  |  |  |
|  | MIC | 0.22 | 0.0225 | 0.01375 | 0.01875 | 0.005 | 0.00625 | 0.02625 | 0 |
|  |  | 0.245 | 0.015 |  |  |  |  |  |  |
|  |  | 0.24 | 0.0125 |  |  |  |  |  |  |
| Composite organic acid salt | 1/4  MIC | 0.275 | 0.0125 | 0.00875 | 0.01125 | 0.0025 | 0.005 | 0.015 | 0 |
|  |  | 0.285 | 0.0075 |  |  |  |  |  |  |
|  |  | 0.29 | 0.01 |  |  |  |  |  |  |
|  | 1/2 MIC | 0.21 | 0.0125 | 0.00875 | 0.01125 | 0.0025 | 0.005 | 0.015 | 0 |
|  |  | 0.225 | 0.01 |  |  |  |  |  |  |
|  |  | 0.22 | 0.0075 |  |  |  |  |  |  |
|  | MIC | 0.145 | 0.01 | 0.00875 | 0.01125 | 0.0025 | 0.005 | 0.015 | 0 |
|  |  | 0.15 | 0.0075 |  |  |  |  |  |  |
|  |  | 0.16 | 0.0125 |  |  |  |  |  |  |

## Relative Conductivity

| Group | | Relative Conductivity | Absolute distance | *Q*1 | *Q*3 | *IQR* | Lower threshold | Upper threshold | Number of outliers |
| --- | --- | --- | --- | --- | --- | --- | --- | --- | --- |
| CK | 0min | 12.07551 | 1.860311 | 2.093226 | 2.790467 | 0.697241 | 1.047364 | 3.836329 | 0 |
|  |  | 11.14385 | 2.32614 |  |  |  |  |  |  |
|  |  | 14.86447 | 3.254794 |  |  |  |  |  |  |
|  | 5min | 12.86076 | 2.438685 | 2.858692 | 3.658027 | 0.799335 | 1.65969 | 4.857029 | 0 |
|  |  | 11.18073 | 3.2787 |  |  |  |  |  |  |
|  |  | 16.0581 | 4.037354 |  |  |  |  |  |  |
|  | 10min | 13.67162 | 1.679487 | 1.841226 | 2.51923 | 0.678004 | 0.82422 | 3.536236 | 0 |
|  |  | 13.02466 | 2.002966 |  |  |  |  |  |  |
|  |  | 16.38363 | 3.035495 |  |  |  |  |  |  |
|  | 20min | 16.65898 | 1.726743 | 1.74607 | 2.590114 | 0.844044 | 0.480004 | 3.85618 | 0 |
|  |  | 13.28281 | 3.41483 |  |  |  |  |  |  |
|  |  | 16.73629 | 1.765398 |  |  |  |  |  |  |
|  | 40min | 18.79282 | 2.919616 | 2.717455 | 3.772941 | 1.055486 | 1.134226 | 5.35617 | 0 |
|  |  | 13.76223 | 4.626266 |  |  |  |  |  |  |
|  |  | 17.98418 | 2.515294 |  |  |  |  |  |  |
|  | 60min | 19.98777 | 2.921319 | 2.556061 | 3.286204 | 0.730143 | 1.460847 | 4.381418 | 0 |
|  |  | 15.60616 | 3.651088 |  |  |  |  |  |  |
|  |  | 18.52673 | 2.190802 |  |  |  |  |  |  |
|  | 80min | 23.4019 | 4.684033 | 4.143437 | 5.404261 | 1.260824 | 2.2522 | 7.295498 | 0 |
|  |  | 16.19622 | 6.12449 |  |  |  |  |  |  |
|  |  | 21.23952 | 3.602841 |  |  |  |  |  |  |
|  | 120min | 24.76755 | 5.350709 | 4.689113 | 6.041276 | 1.352163 | 2.660869 | 8.069521 | 0 |
|  |  | 16.71252 | 6.731844 |  |  |  |  |  |  |
|  |  | 22.12117 | 4.027518 |  |  |  |  |  |  |
| 1/4 MIC | 0min | 15.83558 | 4.525788 | 3.85983 | 4.790807 | 0.930978 | 2.463363 | 6.187274 | 0 |
|  |  | 13.17174 | 3.193871 |  |  |  |  |  |  |
|  |  | 9.447834 | 5.055827 |  |  |  |  |  |  |
|  | 5min | 17.77994 | 5.232509 | 4.450983 | 5.504186 | 1.053203 | 2.871178 | 7.083991 | 0 |
|  |  | 14.65384 | 3.669457 |  |  |  |  |  |  |
|  |  | 10.44102 | 5.775863 |  |  |  |  |  |  |
|  | 10min | 17.40602 | 2.382956 | 2.116893 | 2.776246 | 0.659353 | 1.127864 | 3.765276 | 0 |
|  |  | 16.34177 | 1.850831 |  |  |  |  |  |  |
|  |  | 13.70436 | 3.169537 |  |  |  |  |  |  |
|  | 20min | 16.28428 | 3.192424 | 1.898914 | 2.621345 | 0.72243 | 0.815268 | 3.70499 | 0 |
|  |  | 19.7794 | 2.050265 |  |  |  |  |  |  |
|  |  | 19.174 | 1.747563 |  |  |  |  |  |  |
|  | 40min | 19.67943 | 2.508113 | 1.933606 | 2.390747 | 0.457141 | 1.247895 | 3.076458 | 0 |
|  |  | 22.86709 | 2.273381 |  |  |  |  |  |  |
|  |  | 21.50799 | 1.593831 |  |  |  |  |  |  |
|  | 60min | 22.28188 | 1.05838 | 0.683299 | 0.909367 | 0.226068 | 0.344198 | 1.248469 | 0 |
|  |  | 23.18615 | 0.606245 |  |  |  |  |  |  |
|  |  | 23.49437 | 0.760354 |  |  |  |  |  |  |
|  | 80min | 19.67943 | 4.872743 | 2.500982 | 3.703015 | 1.202033 | 0.697933 | 5.506065 | 0 |
|  |  | 24.61678 | 2.533288 |  |  |  |  |  |  |
|  |  | 24.48756 | 2.468677 |  |  |  |  |  |  |
|  | 120min | 24.63007 | 1.95499 | 1.504852 | 1.86176 | 0.356908 | 0.96949 | 2.397122 | 0 |
|  |  | 26.05771 | 1.241173 |  |  |  |  |  |  |
|  |  | 27.11242 | 1.76853 |  |  |  |  |  |  |
| 1/2 MIC | 0min | 11.04435 | 2.291084 | 2.189743 | 3.132603 | 0.94286 | 0.775453 | 4.546893 | 0 |
|  |  | 11.44972 | 2.088402 |  |  |  |  |  |  |
|  |  | 15.22116 | 3.974122 |  |  |  |  |  |  |
|  | 5min | 12.49906 | 3.348062 | 3.586952 | 5.022093 | 1.435141 | 1.434241 | 7.174804 | 0 |
|  |  | 11.5435 | 3.825842 |  |  |  |  |  |  |
|  |  | 18.23962 | 6.218343 |  |  |  |  |  |  |
|  | 10min | 14.91685 | 2.52089 | 2.684307 | 3.781335 | 1.097027 | 1.038766 | 5.426875 | 0 |
|  |  | 14.26318 | 2.847725 |  |  |  |  |  |  |
|  |  | 19.30496 | 4.714944 |  |  |  |  |  |  |
|  | 20min | 17.49584 | 1.947758 | 2.099446 | 2.921638 | 0.822192 | 0.866158 | 4.154926 | 0 |
|  |  | 16.88909 | 2.251133 |  |  |  |  |  |  |
|  |  | 20.7846 | 3.592142 |  |  |  |  |  |  |
|  | 40min | 22.81498 | 1.157812 | 1.00533 | 1.279273 | 0.273943 | 0.594416 | 1.690187 | 0 |
|  |  | 21.10929 | 1.400734 |  |  |  |  |  |  |
|  |  | 22.20506 | 0.852849 |  |  |  |  |  |  |
|  | 60min | 26.24019 | 3.170141 | 2.39088 | 2.981963 | 0.591083 | 1.504256 | 3.868587 | 0 |
|  |  | 23.87586 | 1.987975 |  |  |  |  |  |  |
|  |  | 22.26424 | 2.793785 |  |  |  |  |  |  |
|  | 80min | 26.32079 | 1.317907 | 1.140419 | 1.444396 | 0.303977 | 0.684453 | 1.900362 | 0 |
|  |  | 25.61083 | 0.962931 |  |  |  |  |  |  |
|  |  | 24.39493 | 1.570885 |  |  |  |  |  |  |
|  | 120min | 27.3685 | 1.263717 | 1.492499 | 1.895575 | 0.403076 | 0.887885 | 2.50019 | 0 |
|  |  | 28.28363 | 1.721282 |  |  |  |  |  |  |
|  |  | 25.75619 | 2.069869 |  |  |  |  |  |  |
| MIC | 0min | 13.69404 | 5.079552 | 5.818161 | 7.619328 | 1.801167 | 3.116411 | 10.32108 | 0 |
|  |  | 20.89871 | 8.681886 |  |  |  |  |  |  |
|  |  | 10.73961 | 6.55677 |  |  |  |  |  |  |
|  | 5min | 15.03166 | 5.267664 | 5.548869 | 7.901496 | 2.352627 | 2.019929 | 11.43044 | 0 |
|  |  | 24.44217 | 9.972918 |  |  |  |  |  |  |
|  |  | 13.90684 | 5.830074 |  |  |  |  |  |  |
|  | 10min | 15.39281 | 9.288355 | 8.041042 | 10.1906 | 2.149553 | 4.816713 | 13.41492 | 0 |
|  |  | 28.98027 | 11.09284 |  |  |  |  |  |  |
|  |  | 20.38206 | 6.79373 |  |  |  |  |  |  |
|  | 20min | 19.21839 | 10.44166 | 7.214187 | 9.326263 | 2.112076 | 4.046074 | 12.49438 | 0 |
|  |  | 31.6534 | 8.210866 |  |  |  |  |  |  |
|  |  | 27.66669 | 6.217508 |  |  |  |  |  |  |
|  | 40min | 21.58596 | 9.858845 | 5.821843 | 8.063449 | 2.241606 | 2.459434 | 11.42586 | 0 |
|  |  | 32.33723 | 6.268053 |  |  |  |  |  |  |
|  |  | 30.55239 | 5.375633 |  |  |  |  |  |  |
|  | 60min | 26.1071 | 10.53953 | 8.149344 | 10.06433 | 1.914986 | 5.276865 | 12.93681 | 0 |
|  |  | 33.76704 | 6.709554 |  |  |  |  |  |  |
|  |  | 39.52621 | 9.589135 |  |  |  |  |  |  |
|  | 80min | 27.846 | 10.17006 | 7.158241 | 9.182455 | 2.024213 | 4.121922 | 12.21877 | 0 |
|  |  | 35.94285 | 6.121636 |  |  |  |  |  |  |
|  |  | 40.08927 | 8.194846 |  |  |  |  |  |  |
|  | 120min | 33.46397 | 5.786024 | 3.837858 | 5.048152 | 1.210294 | 2.022416 | 6.863594 | 0 |
|  |  | 38.30515 | 3.365435 |  |  |  |  |  |  |
|  |  | 40.19484 | 4.310281 |  |  |  |  |  |  |

## pH

| Group | | pH | Absolute distance | *Q*1 | *Q*3 | *IQR* | Lower threshold | Upper threshold | Number of outliers |
| --- | --- | --- | --- | --- | --- | --- | --- | --- | --- |
| CK | 0min | 5.79 | 0.025 | 0.0225 | 0.03 | 0.0075 | 0.01125 | 0.04125 | 0 |
|  |  | 5.78 | 0.02 |  |  |  |  |  |  |
|  |  | 5.75 | 0.035 |  |  |  |  |  |  |
|  | 5min | 5.75 | 0.04 | 0.03 | 0.0375 | 0.0075 | 0.01875 | 0.04875 | 0 |
|  |  | 5.72 | 0.025 |  |  |  |  |  |  |
|  |  | 5.7 | 0.035 |  |  |  |  |  |  |
|  | 10min | 5.69 | 0.045 | 0.0275 | 0.0375 | 0.01 | 0.0125 | 0.0525 | 0 |
|  |  | 5.65 | 0.025 |  |  |  |  |  |  |
|  |  | 5.64 | 0.03 |  |  |  |  |  |  |
|  | 20min | 5.72 | 0.02 | 0.0175 | 0.0225 | 0.005 | 0.01 | 0.03 | 0 |
|  |  | 5.69 | 0.025 |  |  |  |  |  |  |
|  |  | 5.71 | 0.015 |  |  |  |  |  |  |
|  | 40min | 5.72 | 0.02 | 0.01 | 0.015 | 0.005 | 0.0025 | 0.0225 | 0 |
|  |  | 5.74 | 0.01 |  |  |  |  |  |  |
|  |  | 5.74 | 0.01 |  |  |  |  |  |  |
|  | 60min | 5.76 | 0.045 | 0.0275 | 0.0375 | 0.01 | 0.0125 | 0.0525 | 0 |
|  |  | 5.8 | 0.025 |  |  |  |  |  |  |
|  |  | 5.81 | 0.03 |  |  |  |  |  |  |
|  | 80min | 5.79 | 0.045 | 0.045 | 0.0675 | 0.0225 | 0.01125 | 0.10125 | 0 |
|  |  | 5.79 | 0.045 |  |  |  |  |  |  |
|  |  | 5.88 | 0.09 |  |  |  |  |  |  |
|  | 120min | 5.65 | 0.115 | 0.0925 | 0.1125 | 0.02 | 0.0625 | 0.1425 | 0 |
|  |  | 5.73 | 0.075 |  |  |  |  |  |  |
|  |  | 5.8 | 0.11 |  |  |  |  |  |  |
| 1/4 MIC | 0min | 5.7 | 0.025 | 0.0225 | 0.03 | 0.0075 | 0.01125 | 0.04125 | 0 |
|  |  | 5.71 | 0.02 |  |  |  |  |  |  |
|  |  | 5.74 | 0.035 |  |  |  |  |  |  |
|  | 5min | 5.64 | 0.045 | 0.0375 | 0.045 | 0.0075 | 0.02625 | 0.05625 | 0 |
|  |  | 5.58 | 0.045 |  |  |  |  |  |  |
|  |  | 5.61 | 0.03 |  |  |  |  |  |  |
|  | 10min | 5.43 | 0.055 | 0.0425 | 0.0525 | 0.01 | 0.0275 | 0.0675 | 0 |
|  |  | 5.47 | 0.035 |  |  |  |  |  |  |
|  |  | 5.5 | 0.05 |  |  |  |  |  |  |
|  | 20min | 5.61 | 0.02 | 0.0175 | 0.0225 | 0.005 | 0.01 | 0.03 | 0 |
|  |  | 5.64 | 0.025 |  |  |  |  |  |  |
|  |  | 5.62 | 0.015 |  |  |  |  |  |  |
|  | 40min | 5.67 | 0.065 | 0.065 | 0.0975 | 0.0325 | 0.01625 | 0.14625 | 0 |
|  |  | 5.8 | 0.13 |  |  |  |  |  |  |
|  |  | 5.67 | 0.065 |  |  |  |  |  |  |
|  | 60min | 5.62 | 0.16 | 0.13 | 0.1575 | 0.0275 | 0.08875 | 0.19875 | 0 |
|  |  | 5.83 | 0.155 |  |  |  |  |  |  |
|  |  | 5.73 | 0.105 |  |  |  |  |  |  |
|  | 80min | 5.73 | 0.03 | 0.035 | 0.045 | 0.01 | 0.02 | 0.06 | 0 |
|  |  | 5.69 | 0.05 |  |  |  |  |  |  |
|  |  | 5.75 | 0.04 |  |  |  |  |  |  |
|  | 120min | 5.69 | 0.015 | 0.0175 | 0.0225 | 0.005 | 0.01 | 0.03 | 0 |
|  |  | 5.71 | 0.025 |  |  |  |  |  |  |
|  |  | 5.68 | 0.02 |  |  |  |  |  |  |
| 1/2 MIC | 0min | 5.64 | 0.025 | 0.0275 | 0.0375 | 0.01 | 0.0125 | 0.0525 | 0 |
|  |  | 5.65 | 0.03 |  |  |  |  |  |  |
|  |  | 5.6 | 0.045 |  |  |  |  |  |  |
|  | 5min | 5.52 | 0.025 | 0.0175 | 0.0225 | 0.005 | 0.01 | 0.03 | 0 |
|  |  | 5.5 | 0.015 |  |  |  |  |  |  |
|  |  | 5.49 | 0.02 |  |  |  |  |  |  |
|  | 10min | 5.41 | 0.055 | 0.0325 | 0.045 | 0.0125 | 0.01375 | 0.06375 | 0 |
|  |  | 5.47 | 0.035 |  |  |  |  |  |  |
|  |  | 5.46 | 0.03 |  |  |  |  |  |  |
|  | 20min | 5.52 | 0.005 | 0.005 | 0.0075 | 0.0025 | 0.00125 | 0.01125 | 0 |
|  |  | 5.51 | 0.01 |  |  |  |  |  |  |
|  |  | 5.52 | 0.005 |  |  |  |  |  |  |
|  | 40min | 5.55 | 0.035 | 0.03 | 0.0375 | 0.0075 | 0.01875 | 0.04875 | 0 |
|  |  | 5.57 | 0.025 |  |  |  |  |  |  |
|  |  | 5.6 | 0.04 |  |  |  |  |  |  |
|  | 60min | 5.61 | 0.03 | 0.0275 | 0.0375 | 0.01 | 0.0125 | 0.0525 | 0 |
|  |  | 5.62 | 0.025 |  |  |  |  |  |  |
|  |  | 5.66 | 0.045 |  |  |  |  |  |  |
|  | 80min | 5.55 | 0.2 | 0.14 | 0.18 | 0.04 | 0.08 | 0.24 | 0 |
|  |  | 5.79 | 0.16 |  |  |  |  |  |  |
|  |  | 5.71 | 0.12 |  |  |  |  |  |  |
|  | 120min | 5.52 | 0.17 | 0.095 | 0.135 | 0.04 | 0.035 | 0.195 | 0 |
|  |  | 5.7 | 0.1 |  |  |  |  |  |  |
|  |  | 5.68 | 0.09 |  |  |  |  |  |  |
| MIC | 0min | 5.46 | 0.05 | 0.035 | 0.045 | 0.01 | 0.02 | 0.06 | 0 |
|  |  | 5.5 | 0.03 |  |  |  |  |  |  |
|  |  | 5.52 | 0.04 |  |  |  |  |  |  |
|  | 5min | 5.24 | 0.115 | 0.0975 | 0.12 | 0.0225 | 0.06375 | 0.15375 | 0 |
|  |  | 5.4 | 0.125 |  |  |  |  |  |  |
|  |  | 5.31 | 0.08 |  |  |  |  |  |  |
|  | 10min | 5.12 | 0.02 | 0.0225 | 0.03 | 0.0075 | 0.01125 | 0.04125 | 0 |
|  |  | 5.13 | 0.025 |  |  |  |  |  |  |
|  |  | 5.09 | 0.035 |  |  |  |  |  |  |
|  | 20min | 5.22 | 0.115 | 0.0625 | 0.09 | 0.0275 | 0.02125 | 0.13125 | 0 |
|  |  | 5.33 | 0.06 |  |  |  |  |  |  |
|  |  | 5.34 | 0.065 |  |  |  |  |  |  |
|  | 40min | 5.44 | 0.04 | 0.03 | 0.0375 | 0.0075 | 0.01875 | 0.04875 | 0 |
|  |  | 5.47 | 0.025 |  |  |  |  |  |  |
|  |  | 5.49 | 0.035 |  |  |  |  |  |  |
|  | 60min | 5.46 | 0.085 | 0.0675 | 0.0825 | 0.015 | 0.045 | 0.105 | 0 |
|  |  | 5.52 | 0.055 |  |  |  |  |  |  |
|  |  | 5.57 | 0.08 |  |  |  |  |  |  |
|  | 80min | 5.42 | 0.07 | 0.045 | 0.06 | 0.015 | 0.0225 | 0.0825 | 0 |
|  |  | 5.48 | 0.04 |  |  |  |  |  |  |
|  |  | 5.5 | 0.05 |  |  |  |  |  |  |
|  | 120min | 5.34 | 0.125 | 0.0675 | 0.0975 | 0.03 | 0.0225 | 0.1425 | 0 |
|  |  | 5.47 | 0.07 |  |  |  |  |  |  |
|  |  | 5.46 | 0.065 |  |  |  |  |  |  |

## Total Lipids

| Group | | Total lipids | Absolute distance | *Q*1 | *Q*3 | *IQR* | Lower threshold | Upper threshold | Number of outliers |
| --- | --- | --- | --- | --- | --- | --- | --- | --- | --- |
| CK | 0 | 78.28139 | 2.628332 | 2.850936 | 3.580432 | 0.729496 | 1.756692 | 4.674676 | 0 |
|  |  | 80.13732 | 1.791558 |  |  |  |  |  |  |
|  |  | 83.0553 | 3.736881 |  |  |  |  |  |  |
| Sodium diacetate | 1/4  MIC | 44.54144 | 1.808471 | 2.29558 | 3.215468 | 0.919889 | 0.915747 | 4.595301 | 0 |
|  |  | 48.22099 | 3.502759 |  |  |  |  |  |  |
|  |  | 43.9337 | 2.213629 |  |  |  |  |  |  |
|  | 1/2  MIC | 34.34254 | 4.195213 | 2.234808 | 3.074587 | 0.839779 | 0.975139 | 4.334256 | 0 |
|  |  | 37.70166 | 1.955802 |  |  |  |  |  |  |
|  |  | 38.44199 | 1.955802 |  |  |  |  |  |  |
|  | MIC | 30.30469 | 1.885383 | 2.628413 | 3.19184 | 0.563427 | 1.783272 | 4.036981 | 0 |
|  |  | 32.30677 | 3.220102 |  |  |  |  |  |  |
|  |  | 28.05098 | 2.45428 |  |  |  |  |  |  |
|  |  | 28.90433 | 1.885383 |  |  |  |  |  |  |
| Sodium dehydroa-cetate | 1/4  MIC | 34.49247 | 1.281912 | 1.234969 | 1.665584 | 0.430615 | 0.589047 | 2.311506 | 0 |
|  |  | 32.27169 | 1.679126 |  |  |  |  |  |  |
|  |  | 33.99415 | 0.949699 |  |  |  |  |  |  |
|  |  | 33.36583 | 0.949699 |  |  |  |  |  |  |
|  | 1/2  MIC | 22.90903 | 2.446985 | 1.014714 | 1.411301 | 0.396586 | 0.419835 | 2.00618 | 0 |
|  |  | 24.79077 | 1.389421 |  |  |  |  |  |  |
|  |  | 24.49538 | 1.389421 |  |  |  |  |  |  |
|  |  | 26.7819 | 2.716847 |  |  |  |  |  |  |
|  | MIC | 11.49828 | 0.751235 | 1.104972 | 1.403095 | 0.298123 | 0.657787 | 1.850281 | 0 |
|  |  | 10.81998 | 1.203435 |  |  |  |  |  |  |
|  |  | 12.69077 | 1.290957 |  |  |  |  |  |  |
|  |  | 11.88119 | 0.751235 |  |  |  |  |  |  |
| Sodium benzoate | 1/4  MIC | 51.65478 | 2.238548 | 1.566269 | 2.196533 | 0.630264 | 0.620873 | 3.141929 | 0 |
|  |  | 52.06243 | 1.966779 |  |  |  |  |  |  |
|  |  | 54.58349 | 1.966779 |  |  |  |  |  |  |
|  |  | 55.03406 | 2.267158 |  |  |  |  |  |  |
|  | 1/2  MIC | 30.70948 | 1.411301 | 0.984629 | 1.419507 | 0.434878 | 0.332312 | 2.071825 | 0 |
|  |  | 30.86264 | 1.309192 |  |  |  |  |  |  |
|  |  | 32.60216 | 1.309192 |  |  |  |  |  |  |
|  |  | 32.89754 | 1.506116 |  |  |  |  |  |  |
|  | MIC | 13.97079 | 1.794213 | 0.979159 | 1.304633 | 0.325474 | 0.490947 | 1.792845 | 0 |
|  |  | 15.7103 | 0.926281 |  |  |  |  |  |  |
|  |  | 15.27269 | 0.926281 |  |  |  |  |  |  |
|  |  | 16.31202 | 1.327426 |  |  |  |  |  |  |
| Composite organic acid salt | 1/4  MIC | 19.38035 | 0.693316 | 0.300617 | 0.365616 | 0.064999 | 0.203119 | 0.463114 | 0 |
|  |  | 19.64034 | 0.519986 |  |  |  |  |  |  |
|  |  | 19.86784 | 0.519986 |  |  |  |  |  |  |
|  |  | 20.71281 | 1.083304 |  |  |  |  |  |  |
|  | 1/2  MIC | 13.92265 | 0.747698 | 0.776243 | 0.969614 | 0.19337 | 0.486188 | 1.259669 | 0 |
|  |  | 14.69613 | 0.747698 |  |  |  |  |  |  |
|  |  | 15.21547 | 1.093923 |  |  |  |  |  |  |
|  |  | 13.74586 | 0.865562 |  |  |  |  |  |  |
|  | MIC | 5.580111 | 0.320442 | 0.267956 | 0.348066 | 0.08011 | 0.14779 | 0.468231 | 0 |
|  |  | 6.044199 | 0.416206 |  |  |  |  |  |  |
|  |  | 5.900552 | 0.320442 |  |  |  |  |  |  |
|  |  | 5.403315 | 0.438305 |  |  |  |  |  |  |

## Ergosterol

| Group | | Ergosterol | Absolute distance | *Q*1 | *Q*3 | *IQR* | Lower threshold | Upper threshold | Number of outliers |
| --- | --- | --- | --- | --- | --- | --- | --- | --- | --- |
| CK | 0 | 0.21761 | 0.04959 | 0.04675 | 0.065864 | 0.019114 | 0.018079 | 0.094535 | 0 |
|  |  | 0.228972 | 0.043909 |  |  |  |  |  |  |
|  |  | 0.305428 | 0.082137 |  |  |  |  |  |  |
| Sodium diacetate | 1/4  MIC | 0.199024 | 0.069183 | 0.039324 | 0.055437 | 0.016113 | 0.015155 | 0.079605 | 0 |
|  |  | 0.263474 | 0.036958 |  |  |  |  |  |  |
|  |  | 0.27294 | 0.04169 |  |  |  |  |  |  |
|  | 1/2  MIC | 0.178102 | 0.086145 | 0.045166 | 0.066179 | 0.021013 | 0.013647 | 0.097698 | 0 |
|  |  | 0.262153 | 0.044119 |  |  |  |  |  |  |
|  |  | 0.26634 | 0.046213 |  |  |  |  |  |  |
|  | MIC | 0.174395 | 0.085221 | 0.043343 | 0.064465 | 0.021122 | 0.01166 | 0.096149 | 0 |
|  |  | 0.258883 | 0.042977 |  |  |  |  |  |  |
|  |  | 0.260348 | 0.04371 |  |  |  |  |  |  |
| Sodium dehydroa-cetate | 1/4  MIC | 0.19848 | 0.062788 | 0.032729 | 0.048092 | 0.015363 | 0.009683 | 0.071137 | 0 |
|  |  | 0.259934 | 0.032061 |  |  |  |  |  |  |
|  |  | 0.262603 | 0.033396 |  |  |  |  |  |  |
|  | 1/2 MIC | 0.185115 | 0.070737 | 0.037213 | 0.054437 | 0.017223 | 0.011379 | 0.080271 | 0 |
|  |  | 0.254007 | 0.036291 |  |  |  |  |  |  |
|  |  | 0.257697 | 0.038136 |  |  |  |  |  |  |
|  | MIC | 0.179101 | 0.091814 | 0.047087 | 0.069745 | 0.022658 | 0.013099 | 0.103733 | 0 |
|  |  | 0.272094 | 0.047677 |  |  |  |  |  |  |
|  |  | 0.269734 | 0.046497 |  |  |  |  |  |  |
| Sodium benzoate | 1/4  MIC | 0.212922 | 0.065929 | 0.033514 | 0.049859 | 0.016345 | 0.008997 | 0.074376 | 0 |
|  |  | 0.278301 | 0.033239 |  |  |  |  |  |  |
|  |  | 0.279401 | 0.033789 |  |  |  |  |  |  |
|  | 1/2  MIC | 0.192438 | 0.061027 | 0.034322 | 0.048627 | 0.014305 | 0.012865 | 0.070084 | 0 |
|  |  | 0.249657 | 0.032418 |  |  |  |  |  |  |
|  |  | 0.257274 | 0.036226 |  |  |  |  |  |  |
|  | MIC | 0.185368 | 0.074263 | 0.038153 | 0.056464 | 0.01831 | 0.010688 | 0.083929 | 0 |
|  |  | 0.258609 | 0.037643 |  |  |  |  |  |  |
|  |  | 0.260653 | 0.038664 |  |  |  |  |  |  |
| Composite organic acid salt | 1/4  MIC | 0.196871 | 0.103822 | 0.054846 | 0.080067 | 0.025222 | 0.017013 | 0.1179 | 0 |
|  |  | 0.297758 | 0.053378 |  |  |  |  |  |  |
|  |  | 0.303627 | 0.056313 |  |  |  |  |  |  |
|  | 1/2  MIC | 0.181097 | 0.08586 | 0.04384 | 0.065077 | 0.021237 | 0.011984 | 0.096934 | 0 |
|  |  | 0.266047 | 0.043385 |  |  |  |  |  |  |
|  |  | 0.267867 | 0.044295 |  |  |  |  |  |  |
|  | MIC | 0.150998 | 0.095666 | 0.050148 | 0.073486 | 0.023338 | 0.015142 | 0.108492 | 0 |
|  |  | 0.248979 | 0.051306 |  |  |  |  |  |  |
|  |  | 0.244348 | 0.04899 |  |  |  |  |  |  |

## Trehalose

| Group | | Trehalose | Absolute distance | *Q*1 | *Q*3 | *IQR* | Lower threshold | Upper threshold | Number of outliers |
| --- | --- | --- | --- | --- | --- | --- | --- | --- | --- |
| CK | 0 | 46.28934 | 0.299986 | 0.215403 | 0.274047 | 0.058644 | 0.127437 | 0.362013 | 0 |
|  |  | 46.05477 | 0.182698 |  |  |  |  |  |  |
|  |  | 45.92395 | 0.248108 |  |  |  |  |  |  |
| Sodium diacetate | 1/4 MIC | 35.31785 | 0.880977 | 0.813153 | 1.117994 | 0.304841 | 0.355892 | 1.575255 | 0 |
|  |  | 33.82719 | 1.355011 |  |  |  |  |  |  |
|  |  | 35.04655 | 0.745329 |  |  |  |  |  |  |
|  | 1/2 MIC | 21.20711 | 0.388468 | 0.316267 | 0.382876 | 0.066609 | 0.216353 | 0.482789 | 0 |
|  |  | 20.94067 | 0.25525 |  |  |  |  |  |  |
|  |  | 20.6966 | 0.377283 |  |  |  |  |  |  |
|  | MIC | 19.60971 | 0.422964 | 0.287398 | 0.37416 | 0.086762 | 0.157255 | 0.504303 | 0 |
|  |  | 19.26266 | 0.24944 |  |  |  |  |  |  |
|  |  | 19.11083 | 0.325356 |  |  |  |  |  |  |
| Sodium dehydroa-cetate | 1/4 MIC | 23.16642 | 0.575969 | 0.398207 | 0.514644 | 0.116437 | 0.223551 | 0.689299 | 0 |
|  |  | 22.70068 | 0.343096 |  |  |  |  |  |  |
|  |  | 22.48023 | 0.453318 |  |  |  |  |  |  |
|  | 1/2 MIC | 20.41127 | 0.551223 | 0.457413 | 0.549769 | 0.092355 | 0.31888 | 0.688302 | 0 |
|  |  | 21.1443 | 0.548314 |  |  |  |  |  |  |
|  |  | 20.7807 | 0.366513 |  |  |  |  |  |  |
|  | MIC | 16.60711 | 0.449932 | 0.367252 | 0.444164 | 0.076912 | 0.251885 | 0.559531 | 0 |
|  |  | 16.29947 | 0.296109 |  |  |  |  |  |  |
|  |  | 16.01489 | 0.438395 |  |  |  |  |  |  |
| Sodium benzoate | 1/4 MIC | 28.93414 | 0.486844 | 0.381631 | 0.46879 | 0.087159 | 0.250893 | 0.599528 | 0 |
|  |  | 29.5592 | 0.450736 |  |  |  |  |  |  |
|  |  | 29.28278 | 0.312527 |  |  |  |  |  |  |
|  | 1/2 MIC | 22.47943 | 0.192994 | 0.181788 | 0.255873 | 0.074085 | 0.070661 | 0.367001 | 0 |
|  |  | 22.43461 | 0.170582 |  |  |  |  |  |  |
|  |  | 22.13827 | 0.318752 |  |  |  |  |  |  |
|  | MIC | 16.44711 | 0.530019 | 0.498488 | 0.700435 | 0.201947 | 0.195567 | 1.003357 | 0 |
|  |  | 16.57323 | 0.466957 |  |  |  |  |  |  |
|  |  | 17.38102 | 0.870852 |  |  |  |  |  |  |
| Composite organic acid salt | 1/4 MIC | 16.59312 | 0.280838 | 0.243393 | 0.308921 | 0.065529 | 0.1451 | 0.407214 | 0 |
|  |  | 16.44334 | 0.205948 |  |  |  |  |  |  |
|  |  | 16.18123 | 0.337005 |  |  |  |  |  |  |
|  | 1/2 MIC | 14.30018 | 0.136888 | 0.157472 | 0.205332 | 0.047859 | 0.085684 | 0.277121 | 0 |
|  |  | 14.49161 | 0.232606 |  |  |  |  |  |  |
|  |  | 14.21784 | 0.178057 |  |  |  |  |  |  |
|  | MIC | 14.33452 | 0.43352 | 0.371395 | 0.463906 | 0.092511 | 0.232629 | 0.602673 | 0 |
|  |  | 14.08602 | 0.309271 |  |  |  |  |  |  |
|  |  | 13.71597 | 0.494293 |  |  |  |  |  |  |

## Superoxide Dismutase

| Group | | Superoxide dismutase | Absolute distance | *Q*1 | *Q*3 | *IQR* | Lower threshold | Upper threshold | Number of outliers |
| --- | --- | --- | --- | --- | --- | --- | --- | --- | --- |
| CK | 0 | 87.8742 | 1.44061 | 1.760747 | 2.160915 | 0.400168 | 1.160495 | 2.761167 | 0 |
|  |  | 89.15474 | 2.080884 |  |  |  |  |  |  |
|  |  | 86.27352 | 2.240946 |  |  |  |  |  |  |
| Sodium diacetate | 1/4  MIC | 102.4502 | 0.721847 | 0.536855 | 0.673334 | 0.136479 | 0.332137 | 0.878052 | 0 |
|  |  | 101.9043 | 0.448889 |  |  |  |  |  |  |
|  |  | 101.5524 | 0.624821 |  |  |  |  |  |  |
|  | 1/2  MIC | 102.2457 | 5.712092 | 3.900662 | 5.067531 | 1.166869 | 2.150359 | 6.817834 | 0 |
|  |  | 109.0024 | 4.422971 |  |  |  |  |  |  |
|  |  | 106.9131 | 3.378354 |  |  |  |  |  |  |
|  | MIC | 114.7367 | 4.020457 | 2.698163 | 3.531293 | 0.833131 | 1.448466 | 4.78099 | 0 |
|  |  | 110.0283 | 3.042129 |  |  |  |  |  |  |
|  |  | 111.4042 | 2.354196 |  |  |  |  |  |  |
| Sodium dehydroa-cetate | 1/4  MIC | 102.7971 | 4.385764 | 3.160975 | 4.015393 | 0.854418 | 1.879349 | 5.297019 | 0 |
|  |  | 108.151 | 3.645022 |  |  |  |  |  |  |
|  |  | 106.2148 | 2.676928 |  |  |  |  |  |  |
|  | 1/2  MIC | 106.8036 | 0.645216 | 0.596932 | 0.822973 | 0.226041 | 0.257871 | 1.162034 | 0 |
|  |  | 107.9009 | 1.00073 |  |  |  |  |  |  |
|  |  | 106.9967 | 0.548649 |  |  |  |  |  |  |
|  | MIC | 115.1789 | 1.501397 | 0.875582 | 1.219711 | 0.344128 | 0.35939 | 1.735903 | 0 |
|  |  | 116.8052 | 0.938024 |  |  |  |  |  |  |
|  |  | 116.5555 | 0.81314 |  |  |  |  |  |  |
| Sodium benzoate | 1/4  MIC | 110.5693 | 0.449083 | 0.540918 | 0.673625 | 0.132707 | 0.341858 | 0.872684 | 0 |
|  |  | 111.1001 | 0.714496 |  |  |  |  |  |  |
|  |  | 110.2019 | 0.632753 |  |  |  |  |  |  |
|  | 1/2  MIC | 118.393 | 2.892608 | 2.578386 | 3.396246 | 0.81786 | 1.351597 | 4.623035 | 0 |
|  |  | 117.1362 | 2.264164 |  |  |  |  |  |  |
|  |  | 113.8647 | 3.899883 |  |  |  |  |  |  |
|  | MIC | 119.8723 | 1.725739 | 2.026422 | 2.588609 | 0.562186 | 1.183143 | 3.431888 | 0 |
|  |  | 121.0751 | 2.327105 |  |  |  |  |  |  |
|  |  | 117.6236 | 2.850112 |  |  |  |  |  |  |
| Composite organic acid salt | 1/4  MIC | 109.4776 | 3.851301 | 3.280729 | 4.065237 | 0.784508 | 2.103968 | 5.241998 | 0 |
|  |  | 107.1953 | 2.710158 |  |  |  |  |  |  |
|  |  | 104.0573 | 4.279173 |  |  |  |  |  |  |
|  | 1/2  MIC | 113.0804 | 3.41979 | 4.245322 | 5.129684 | 0.884362 | 2.91878 | 6.456227 | 0 |
|  |  | 116.6178 | 5.188513 |  |  |  |  |  |  |
|  |  | 109.7783 | 5.070855 |  |  |  |  |  |  |
|  | MIC | 117.6509 | 6.683938 | 5.440364 | 6.58675 | 1.146386 | 3.720785 | 8.306329 | 0 |
|  |  | 126.4332 | 6.489561 |  |  |  |  |  |  |
|  |  | 122.2364 | 4.391167 |  |  |  |  |  |  |

## Catalase

| Group | | Catalase | Absolute distance | *Q*1 | *Q*3 | *IQR* | Lower threshold | Upper threshold | Number of outliers |
| --- | --- | --- | --- | --- | --- | --- | --- | --- | --- |
| CK | 0 | 1407.869 | 30.87432 | 21.61202 | 27.78689 | 6.174863 | 12.34973 | 37.04918 | 0 |
|  |  | 1370.82 | 24.69945 |  |  |  |  |  |  |
|  |  | 1383.169 | 18.52459 |  |  |  |  |  |  |
| Sodium diacetate | 1/4 MIC | 1209.577 | 103.4507 | 59.6831 | 83.55634 | 23.87324 | 23.87324 | 119.3662 | 0 |
|  |  | 1320.986 | 63.66197 |  |  |  |  |  |  |
|  |  | 1305.07 | 55.70423 |  |  |  |  |  |  |
|  | 1/2 MIC | 833.4219 | 11.26246 | 11.26246 | 16.89369 | 5.631229 | 2.815615 | 25.34053 | 0 |
|  |  | 833.4219 | 11.26246 |  |  |  |  |  |  |
|  |  | 810.897 | 22.52492 |  |  |  |  |  |  |
|  | MIC | 757.7647 | 19.94118 | 19.94118 | 29.91176 | 9.970588 | 4.985294 | 44.86765 | 0 |
|  |  | 757.7647 | 19.94118 |  |  |  |  |  |  |
|  |  | 797.6471 | 39.88235 |  |  |  |  |  |  |
| Sodium  dehydroa-cetate | 1/4 MIC | 834.2578 | 72.83203 | 66.21094 | 89.38477 | 23.17383 | 31.4502 | 124.1455 | 0 |
|  |  | 953.4375 | 105.9375 |  |  |  |  |  |  |
|  |  | 860.7422 | 59.58984 |  |  |  |  |  |  |
|  | 1/2 MIC | 809.5522 | 61.8408 | 56.21891 | 75.89552 | 19.67662 | 26.70398 | 105.4104 | 0 |
|  |  | 910.7463 | 89.95025 |  |  |  |  |  |  |
|  |  | 832.0398 | 50.59701 |  |  |  |  |  |  |
|  | MIC | 727.1924 | 10.69401 | 10.69401 | 16.04101 | 5.347003 | 2.673502 | 24.06151 | 0 |
|  |  | 727.1924 | 10.69401 |  |  |  |  |  |  |
|  |  | 748.5804 | 21.38801 |  |  |  |  |  |  |
| Sodium benzoate | 1/4 MIC | 1376.201 | 18.93855 | 22.09497 | 28.40782 | 6.312849 | 12.6257 | 37.87709 | 0 |
|  |  | 1388.827 | 25.2514 |  |  |  |  |  |  |
|  |  | 1350.95 | 31.56425 |  |  |  |  |  |  |
|  | 1/2 MIC | 1134.612 | 20.7551 | 24.21429 | 31.13265 | 6.918367 | 13.83673 | 41.5102 | 0 |
|  |  | 1148.449 | 27.67347 |  |  |  |  |  |  |
|  |  | 1106.939 | 34.59184 |  |  |  |  |  |  |
|  | MIC | 760.0897 | 30.40359 | 30.40359 | 45.60538 | 15.20179 | 7.600897 | 68.40807 | 0 |
|  |  | 760.0897 | 30.40359 |  |  |  |  |  |  |
|  |  | 820.8969 | 60.80717 |  |  |  |  |  |  |
| Composite  organic acid salt | 1/4 MIC | 1079.534 | 118.4854 | 138.233 | 177.7282 | 39.49515 | 78.99029 | 236.9709 | 0 |
|  |  | 1237.515 | 197.4757 |  |  |  |  |  |  |
|  |  | 1000.544 | 157.9806 |  |  |  |  |  |  |
|  | 1/2 MIC | 954.466 | 16.45631 | 8.228155 | 12.34223 | 4.114078 | 2.057039 | 18.51335 | 0 |
|  |  | 938.0097 | 8.228155 |  |  |  |  |  |  |
|  |  | 938.0097 | 8.228155 |  |  |  |  |  |  |
|  | MIC | 671.8919 | 30.54054 | 30.54054 | 45.81081 | 15.27027 | 7.635135 | 68.71622 | 0 |
|  |  | 732.973 | 61.08108 |  |  |  |  |  |  |
|  |  | 671.8919 | 30.54054 |  |  |  |  |  |  |

## Glutathione Peroxidase

| Group | | Glutathione peroxidase | Absolute distance | *Q*1 | *Q*3 | *IQR* | Lower threshold | Upper threshold | Number of outliers |
| --- | --- | --- | --- | --- | --- | --- | --- | --- | --- |
| CK | 0 | 224.8124 | 23.66446 | 23.66446 | 35.49669 | 11.83223 | 5.916115 | 53.24503 | 0 |
|  |  | 177.4834 | 47.32892 |  |  |  |  |  |  |
|  |  | 224.8124 | 23.66446 |  |  |  |  |  |  |
| Sodium diacetate | 1/4  MIC | 417.6623 | 69.61039 | 44.74954 | 59.66605 | 14.91651 | 22.37477 | 82.04082 | 0 |
|  |  | 338.1076 | 49.72171 |  |  |  |  |  |  |
|  |  | 357.9963 | 39.77737 |  |  |  |  |  |  |
|  | 1/2  MIC | 330.411 | 110.137 | 94.84018 | 119.3151 | 24.47489 | 58.12785 | 156.0274 | 0 |
|  |  | 489.4977 | 128.4932 |  |  |  |  |  |  |
|  |  | 391.5982 | 79.54338 |  |  |  |  |  |  |
|  | MIC | 573.5409 | 78.21012 | 67.7821 | 86.03113 | 18.24903 | 40.40856 | 113.4047 | 0 |
|  |  | 688.249 | 93.85214 |  |  |  |  |  |  |
|  |  | 615.2529 | 57.35409 |  |  |  |  |  |  |
| Sodium Dehydroa-  cetate | 1/4  MIC | 279.9226 | 20.73501 | 23.32689 | 31.10251 | 7.775629 | 11.66344 | 42.76596 | 0 |
|  |  | 248.8201 | 36.28627 |  |  |  |  |  |  |
|  |  | 290.2901 | 25.91876 |  |  |  |  |  |  |
|  | 1/2  MIC | 283.5979 | 28.35979 | 35.44974 | 42.53968 | 7.089947 | 24.81481 | 53.1746 | 0 |
|  |  | 255.2381 | 42.53968 |  |  |  |  |  |  |
|  |  | 311.9577 | 42.53968 |  |  |  |  |  |  |
|  | MIC | 312.315 | 29.54331 | 31.65354 | 44.31496 | 12.66142 | 12.66142 | 63.30709 | 0 |
|  |  | 320.7559 | 33.76378 |  |  |  |  |  |  |
|  |  | 261.6693 | 54.86614 |  |  |  |  |  |  |
| Sodium benzoate | 1/4  MIC | 387.4699 | 56.50602 | 36.3253 | 48.43373 | 12.10843 | 18.16265 | 66.59639 | 0 |
|  |  | 322.8916 | 40.36145 |  |  |  |  |  |  |
|  |  | 339.0361 | 32.28916 |  |  |  |  |  |  |
|  | 1/2  MIC | 388.1894 | 29.86072 | 26.12813 | 33.59331 | 7.465181 | 14.93036 | 44.79109 | 0 |
|  |  | 432.9805 | 37.32591 |  |  |  |  |  |  |
|  |  | 403.1198 | 22.39554 |  |  |  |  |  |  |
|  | MIC | 580.805 | 24.89164 | 20.74303 | 24.89164 | 4.148607 | 14.52012 | 31.11455 | 0 |
|  |  | 597.3994 | 16.59443 |  |  |  |  |  |  |
|  |  | 613.9938 | 24.89164 |  |  |  |  |  |  |
| Composite  organic acid salt | 1/4  MIC | 273.8686 | 34.23358 | 29.34307 | 36.67883 | 7.335766 | 18.33942 | 47.68248 | 0 |
|  |  | 224.9635 | 39.12409 |  |  |  |  |  |  |
|  |  | 254.3066 | 24.45255 |  |  |  |  |  |  |
|  | 1/2  MIC | 335 | 10.46875 | 13.08594 | 15.70312 | 2.617188 | 9.160156 | 19.62891 | 0 |
|  |  | 345.4688 | 15.70312 |  |  |  |  |  |  |
|  |  | 324.5313 | 15.70312 |  |  |  |  |  |  |
|  | MIC | 722.6966 | 15.05618 | 15.05618 | 22.58427 | 7.52809 | 3.764045 | 33.8764 | 0 |
|  |  | 752.809 | 30.11236 |  |  |  |  |  |  |
|  |  | 722.6966 | 15.05618 |  |  |  |  |  |  |

## Malondialdehyde

| Group | | Malondialdehyde | Absolute distance | *Q*1 | *Q*3 | *IQR* | Lower threshold | Upper threshold | Number of outliers |
| --- | --- | --- | --- | --- | --- | --- | --- | --- | --- |
| CK | 0 | 5.771144 | 0.796051 | 0.912137 | 1.194077 | 0.281939 | 0.489228 | 1.616986 | 0 |
|  |  | 6.898901 | 1.35993 |  |  |  |  |  |  |
|  |  | 5.306799 | 1.028223 |  |  |  |  |  |  |
| Sodium diacetate | 1/4  MIC | 12.24172 | 1.247564 | 0.779727 | 1.052632 | 0.272905 | 0.37037 | 1.461989 | 0 |
|  |  | 10.83821 | 0.8577 |  |  |  |  |  |  |
|  |  | 11.1501 | 0.701755 |  |  |  |  |  |  |
|  | 1/2  MIC | 10.99415 | 1.091615 | 0.62378 | 0.877191 | 0.25341 | 0.243665 | 1.257306 | 0 |
|  |  | 12.0078 | 0.584794 |  |  |  |  |  |  |
|  |  | 12.16374 | 0.662767 |  |  |  |  |  |  |
|  | MIC | 13.96947 | 0.267173 | 0.286257 | 0.40076 | 0.114503 | 0.114502 | 0.572515 | 0 |
|  |  | 14.0458 | 0.305341 |  |  |  |  |  |  |
|  |  | 13.51145 | 0.49618 |  |  |  |  |  |  |
| Sodium Dehydroa  -cetate | 1/4  MIC | 9.602764 | 0.345423 | 0.310881 | 0.414508 | 0.103627 | 0.15544 | 0.569949 | 0 |
|  |  | 10.15544 | 0.483593 |  |  |  |  |  |  |
|  |  | 9.740933 | 0.276339 |  |  |  |  |  |  |
|  | 1/2  MIC | 8.701754 | 3.614036 | 3.052633 | 3.736844 | 0.684211 | 2.026316 | 4.76316 | 0 |
|  |  | 13.68421 | 3.859651 |  |  |  |  |  |  |
|  |  | 10.94737 | 2.491229 |  |  |  |  |  |  |
|  | MIC | 15.01946 | 2.645915 | 1.556421 | 2.159534 | 0.603113 | 0.651751 | 3.064203 | 0 |
|  |  | 12.607 | 1.439689 |  |  |  |  |  |  |
|  |  | 12.14008 | 1.673153 |  |  |  |  |  |  |
| Sodium benzoate | 1/4  MIC | 8.582679 | 1.259842 | 1.318897 | 1.889763 | 0.570866 | 0.462599 | 2.746061 | 0 |
|  |  | 10.86614 | 2.401573 |  |  |  |  |  |  |
|  |  | 8.346457 | 1.377953 |  |  |  |  |  |  |
|  | 1/2  MIC | 10.85803 | 2.215288 | 2.76131 | 3.322932 | 0.561622 | 1.918876 | 4.165365 | 0 |
|  |  | 13.10452 | 3.338532 |  |  |  |  |  |  |
|  |  | 8.673947 | 3.307331 |  |  |  |  |  |  |
|  | MIC | 14.9885 | 1.747125 | 1.149426 | 1.517242 | 0.367815 | 0.597703 | 2.068965 | 0 |
|  |  | 13.51724 | 1.011494 |  |  |  |  |  |  |
|  |  | 12.96552 | 1.287358 |  |  |  |  |  |  |
| Composite  organic acid salt | 1/4  MIC | 9.573462 | 2.417059 | 1.729857 | 2.20379 | 0.473933 | 1.018957 | 2.914689 | 0 |
|  |  | 12.51185 | 1.99052 |  |  |  |  |  |  |
|  |  | 11.46919 | 1.469193 |  |  |  |  |  |  |
|  | 1/2  MIC | 13.63265 | 2.244897 | 1.918368 | 2.387757 | 0.469389 | 1.214284 | 3.091841 | 0 |
|  |  | 12.32653 | 1.591838 |  |  |  |  |  |  |
|  |  | 10.44898 | 2.530616 |  |  |  |  |  |  |
|  | MIC | 15.61165 | 2.330096 | 2.582523 | 3.495144 | 0.912621 | 1.213592 | 4.864075 | 0 |
|  |  | 16.62136 | 2.83495 |  |  |  |  |  |  |
|  |  | 11.96117 | 4.155338 |  |  |  |  |  |  |

## Gene

| Column name | | Gene | Absolute distance | *Q*1 | *Q*3 | *IQR* | Lower threshold | Upper threshold | Number of outliers |
| --- | --- | --- | --- | --- | --- | --- | --- | --- | --- |
| *aflr* | CK | 1.009878 | 0.092845 | 0.113441 | 0.139267 | 0.025826 | 0.074702 | 0.178006 | 0 |
|  |  | 0.906575 | 0.144496 |  |  |  |  |  |  |
|  |  | 1.092264 | 0.134037 |  |  |  |  |  |  |
|  | MIC | 0.129371 | 0.00157 | 0.001631 | 0.002355 | 0.000723 | 0.000546 | 0.00344 | 0 |
|  |  | 0.126477 | 0.003017 |  |  |  |  |  |  |
|  |  | 0.129617 | 0.001693 |  |  |  |  |  |  |
|  | 1/2 MIC | 0.820551 | 0.139126 | 0.083312 | 0.114657 | 0.031344 | 0.036296 | 0.161673 | 0 |
|  |  | 0.945928 | 0.076438 |  |  |  |  |  |  |
|  |  | 0.973427 | 0.090187 |  |  |  |  |  |  |
| *afls* | CK | 1.048461 | 0.112937 | 0.124837 | 0.169405 | 0.044568 | 0.057984 | 0.236258 | 0 |
|  |  | 0.870188 | 0.202073 |  |  |  |  |  |  |
|  |  | 1.096061 | 0.136737 |  |  |  |  |  |  |
|  | MIC | 0.103174 | 0.021247 | 0.025972 | 0.03187 | 0.005897 | 0.017126 | 0.040716 | 0 |
|  |  | 0.084271 | 0.030698 |  |  |  |  |  |  |
|  |  | 0.126764 | 0.033041 |  |  |  |  |  |  |
|  | 1/2 MIC | 0.767279 | 0.109325 | 0.067494 | 0.091617 | 0.024123 | 0.031309 | 0.127802 | 0 |
|  |  | 0.863773 | 0.061078 |  |  |  |  |  |  |
|  |  | 0.889436 | 0.07391 |  |  |  |  |  |  |
| *afld* | CK | 0.957936 | 0.148406 | 0.166881 | 0.222609 | 0.055728 | 0.08329 | 0.306201 | 0 |
|  |  | 0.884035 | 0.185356 |  |  |  |  |  |  |
|  |  | 1.180847 | 0.259861 |  |  |  |  |  |  |
|  | MIC | 0.177152 | 0.040224 | 0.050251 | 0.060336 | 0.010086 | 0.035122 | 0.075465 | 0 |
|  |  | 0.137046 | 0.060277 |  |  |  |  |  |  |
|  |  | 0.217495 | 0.060396 |  |  |  |  |  |  |
|  | 1/2 MIC | 1.113226 | 0.230922 | 0.199645 | 0.252553 | 0.052908 | 0.120284 | 0.331914 | 0 |
|  |  | 1.449963 | 0.274184 |  |  |  |  |  |  |
|  |  | 1.238332 | 0.168368 |  |  |  |  |  |  |
| *aflm* | CK | 1.203855 | 0.287191 | 0.239897 | 0.288905 | 0.049008 | 0.166385 | 0.362417 | 0 |
|  |  | 0.818648 | 0.29062 |  |  |  |  |  |  |
|  |  | 1.014679 | 0.192604 |  |  |  |  |  |  |
|  | MIC | 0.160663 | 0.062306 | 0.07172 | 0.093459 | 0.021738 | 0.039113 | 0.126066 | 0 |
|  |  | 0.07371 | 0.105783 |  |  |  |  |  |  |
|  |  | 0.198322 | 0.081135 |  |  |  |  |  |  |
|  | 1/2 MIC | 1.145639 | 0.11867 | 0.101865 | 0.127589 | 0.025725 | 0.063278 | 0.166176 | 0 |
|  |  | 1.21286 | 0.085059 |  |  |  |  |  |  |
|  |  | 1.315758 | 0.136508 |  |  |  |  |  |  |
| *aflc* | CK | 0.933382 | 0.102592 | 0.087756 | 0.109379 | 0.021623 | 0.055321 | 0.141814 | 0 |
|  |  | 0.992728 | 0.072919 |  |  |  |  |  |  |
|  |  | 1.079221 | 0.116166 |  |  |  |  |  |  |
|  | MIC | 0.122105 | 0.007646 | 0.006546 | 0.00817 | 0.001624 | 0.004111 | 0.010605 | 0 |
|  |  | 0.117706 | 0.005447 |  |  |  |  |  |  |
|  |  | 0.111212 | 0.008694 |  |  |  |  |  |  |
|  | 1/2 MIC | 0.563985 | 0.2439 | 0.165076 | 0.21527 | 0.050193 | 0.089786 | 0.29056 | 0 |
|  |  | 0.851011 | 0.186639 |  |  |  |  |  |  |
|  |  | 0.764759 | 0.143513 |  |  |  |  |  |  |
| *aflt* | CK | 1.149332 | 0.216542 | 0.113475 | 0.166309 | 0.052835 | 0.034223 | 0.245561 | 0 |
|  |  | 0.927587 | 0.116076 |  |  |  |  |  |  |
|  |  | 0.937994 | 0.110873 |  |  |  |  |  |  |
|  | MIC | 0.118617 | 0.005033 | 0.003649 | 0.004624 | 0.000975 | 0.002186 | 0.006087 | 0 |
|  |  | 0.114716 | 0.003083 |  |  |  |  |  |  |
|  |  | 0.112451 | 0.004215 |  |  |  |  |  |  |
|  | 1/2 MIC | 0.769147 | 0.028577 | 0.027779 | 0.040472 | 0.012693 | 0.00874 | 0.059511 | 0 |
|  |  | 0.765956 | 0.026981 |  |  |  |  |  |  |
|  |  | 0.715184 | 0.052367 |  |  |  |  |  |  |

# Z-score normalization

## Mycelium Dry Weight

| Group | | Mycelium dry weight (mg/mL) | | | Z-score normalization | | |
| --- | --- | --- | --- | --- | --- | --- | --- |
| CK | 0-24h | 0.27 | 0.31 | 0.29 | -1 | 1 | -2.8E-15 |
|  | 0-48h | 1.07 | 1.21 | 0.995 | -0.19855 | 1.08438 | -0.88583 |
| Sodium diacetate | 1/4 MIC | 0.235 | 0.255 | 0.245 | -1 | 1 | 2.78E-15 |
|  | 1/2 MIC | 0.24 | 0.24 | 0.245 | -0.57735 | -0.57735 | 1.154701 |
|  | MIC | 0.18 | 0.215 | 0.185 | -0.70436 | 1.144586 | -0.44023 |
| Sodium dehydroacetate | 1/4 MIC | 0.465 | 0.435 | 0.46 | 0.725866 | -1.14065 | 0.414781 |
|  | 1/2 MIC | 0.29 | 0.325 | 0.305 | -0.94916 | 1.044074 | -0.09492 |
|  | MIC | 0.24 | 0.245 | 0.25 | -1 | 5.55E-15 | 1 |
| Sodium benzoate | 1/4 MIC | 0.355 | 0.345 | 0.365 | 0 | -1 | 1 |
|  | 1/2 MIC | 0.315 | 0.32 | 0.315 | -0.57735 | 1.154701 | -0.57735 |
|  | MIC | 0.22 | 0.245 | 0.24 | -1.13389 | 0.755929 | 0.377964 |
| Composite organic acid salt | 1/4 MIC | 0.275 | 0.285 | 0.29 | -1.09109 | 0.218218 | 0.872872 |
|  | 1/2 MIC | 0.21 | 0.225 | 0.22 | -1.09109 | 0.872872 | 0.218218 |
|  | MIC | 0.145 | 0.15 | 0.16 | -0.87287 | -0.21822 | 1.091089 |

## Relative Conductivity

| CK | | | | Z-score normalization | | |
| --- | --- | --- | --- | --- | --- | --- |
| 0min | 12.07551 | 11.14385 | 14.86447 | -0.31978 | -0.801 | 1.120778 |
| 5min | 12.86076 | 11.18073 | 16.0581 | -0.20413 | -0.88219 | 1.086314 |
| 10min | 13.67162 | 13.02466 | 16.38363 | -0.38625 | -0.74927 | 1.13552 |
| 20min | 16.65898 | 13.28281 | 16.73629 | 0.557637 | -1.15448 | 0.596842 |
| 40min | 18.79282 | 13.76223 | 17.98418 | 0.720518 | -1.14169 | 0.421176 |
| 60min | 19.98777 | 15.60616 | 18.52673 | 0.872967 | -1.09104 | 0.218074 |
| 80min | 23.4019 | 16.19622 | 21.23952 | 0.844522 | -1.10423 | 0.259712 |
| 120min | 24.76755 | 16.71252 | 22.12117 | 0.868827 | -1.09309 | 0.224263 |
| 1/4 MIC | | | | Z-score normalization | | |
| 0min | 15.83558 | 13.17174 | 9.447834 | 0.940375 | 0.110132 | -1.05051 |
| 5min | 17.77994 | 14.65384 | 10.44102 | 0.947187 | 0.098358 | -1.04554 |
| 10min | 17.40602 | 16.34177 | 13.70436 | 0.83361 | 0.275163 | -1.10877 |
| 20min | 16.28428 | 19.7794 | 19.174 | -1.13944 | 0.731778 | 0.407658 |
| 40min | 19.67943 | 22.86709 | 21.50799 | -1.04532 | 0.947489 | 0.097831 |
| 60min | 22.28188 | 23.18615 | 23.49437 | -1.11964 | 0.315277 | 0.804365 |
| 80min | 19.67943 | 24.61678 | 24.48756 | -1.1544 | 0.600158 | 0.554238 |
| 120min | 24.63007 | 26.05771 | 27.11242 | -1.04615 | 0.099778 | 0.946371 |
| 1/2 MIC | | | | Z-score normalization | | |
| 0min | 11.04435 | 11.44972 | 15.22116 | -0.6631 | -0.48712 | 1.150222 |
| 5min | 12.49906 | 11.5435 | 18.23962 | -0.44039 | -0.70422 | 1.144609 |
| 10min | 14.91685 | 14.26318 | 19.30496 | -0.45403 | -0.69244 | 1.146467 |
| 20min | 17.49584 | 16.88909 | 20.7846 | -0.42653 | -0.71601 | 1.142541 |
| 40min | 22.81498 | 21.10929 | 22.20506 | 0.893059 | -1.08043 | 0.187374 |
| 60min | 26.24019 | 23.87586 | 22.26424 | 1.056811 | -0.12546 | -0.93135 |
| 80min | 26.32079 | 25.61083 | 24.39493 | 0.902109 | 0.173164 | -1.07527 |
| 120min | 27.3685 | 28.28363 | 25.75619 | 0.181606 | 0.896751 | -1.07836 |
| MIC | | | | Z-score normalization | | |
| 0min | 13.69404 | 20.89871 | 10.73961 | -0.27111 | 1.107603 | -0.83649 |
| 5min | 15.03166 | 24.44217 | 13.90684 | -0.4774 | 1.149231 | -0.67183 |
| 10min | 15.39281 | 28.98027 | 20.38206 | -0.90093 | 1.075959 | -0.17503 |
| 20min | 19.21839 | 31.6534 | 27.66669 | -1.09632 | 0.8621 | 0.234222 |
| 40min | 21.58596 | 32.33723 | 30.55239 | -1.14076 | 0.725275 | 0.41549 |
| 60min | 26.1071 | 33.76704 | 39.52621 | -1.04373 | 0.094117 | 0.949614 |
| 80min | 27.846 | 35.94285 | 40.08927 | -1.08882 | 0.21147 | 0.877352 |
| 120min | 33.46397 | 38.30515 | 40.19484 | -1.11111 | 0.283392 | 0.827719 |

## pH

| CK | | | | Z-score normalization | | |
| --- | --- | --- | --- | --- | --- | --- |
| 0min | 5.79 | 5.78 | 5.75 | 0.800641 | 0.320256 | -1.1209 |
| 5min | 5.75 | 5.72 | 5.7 | 1.059626 | -0.13245 | -0.92717 |
| 10min | 5.69 | 5.65 | 5.64 | 1.133893 | -0.37796 | -0.75593 |
| 20min | 5.72 | 5.69 | 5.71 | 0.872872 | -1.09109 | 0.218218 |
| 40min | 5.72 | 5.74 | 5.74 | -1.1547 | 0.57735 | 0.57735 |
| 60min | 5.76 | 5.8 | 5.81 | -1.13389 | 0.377964 | 0.755929 |
| 80min | 5.79 | 5.79 | 5.88 | -0.57735 | -0.57735 | 1.154701 |
| 120min | 5.65 | 5.73 | 5.8 | -1.02147 | 0.044412 | 0.977054 |
| 1/4 MIC | | | | Z-score normalization | | |
| 0min | 5.7 | 5.71 | 5.74 | -0.80064 | -0.32026 | 1.120897 |
| 5min | 5.64 | 5.58 | 5.61 | 1 | -1 | 2.96E-14 |
| 10min | 5.43 | 5.47 | 5.5 | -1.04407 | 0.094916 | 0.949158 |
| 20min | 5.61 | 5.64 | 5.62 | -0.87287 | 1.091089 | -0.21822 |
| 40min | 5.67 | 5.8 | 5.67 | -0.57735 | 1.154701 | -0.57735 |
| 60min | 5.62 | 5.83 | 5.73 | -1.01549 | 0.983755 | 0.031734 |
| 80min | 5.73 | 5.69 | 5.75 | 0.218218 | -1.09109 | 0.872872 |
| 120min | 5.69 | 5.71 | 5.68 | -0.21822 | 1.091089 | -0.87287 |
| 1/2 MIC | | | | Z-score normalization | | |
| 0min | 5.64 | 5.65 | 5.6 | 0.377964 | 0.755929 | -1.13389 |
| 5min | 5.52 | 5.5 | 5.49 | 1.091089 | -0.21822 | -0.87287 |
| 10min | 5.41 | 5.47 | 5.46 | -1.14065 | 0.725866 | 0.414781 |
| 20min | 5.52 | 5.51 | 5.52 | 0.57735 | -1.1547 | 0.57735 |
| 40min | 5.55 | 5.57 | 5.6 | -0.92717 | -0.13245 | 1.059626 |
| 60min | 5.61 | 5.62 | 5.66 | -0.75593 | -0.37796 | 1.133893 |
| 80min | 5.55 | 5.79 | 5.71 | -1.09109 | 0.872872 | 0.218218 |
| 120min | 5.52 | 5.7 | 5.68 | -1.14875 | 0.675737 | 0.473016 |
| MIC | | | | Z-score normalization | | |
| 0min | 5.46 | 5.5 | 5.52 | -1.09109 | 0.218218 | 0.872872 |
| 5min | 5.24 | 5.4 | 5.31 | -0.95585 | 1.038965 | -0.08312 |
| 10min | 5.12 | 5.13 | 5.09 | 0.320256 | 0.800641 | -1.1209 |
| 20min | 5.22 | 5.33 | 5.34 | -1.15144 | 0.500626 | 0.650814 |
| 40min | 5.44 | 5.47 | 5.49 | -1.05963 | 0.132453 | 0.927173 |
| 60min | 5.46 | 5.52 | 5.57 | -1.02889 | 0.060523 | 0.968364 |
| 80min | 5.42 | 5.48 | 5.5 | -1.1209 | 0.320256 | 0.800641 |
| 120min | 5.34 | 5.47 | 5.46 | -1.15194 | 0.645086 | 0.506853 |

## Total Lipids

| Group | | Lipid (mg/g) | | | Z-score normalization | | |
| --- | --- | --- | --- | --- | --- | --- | --- |
| CK | 0 | 78.2814 | 80.1373 | 83.0553 | -0.9183 | -0.14711 | 1.065405 |
| Sodium diacetate | 1/4 MIC | 44.5414 | 48.2210 | 43.9337 | -0.44139 | 1.144752 | -0.70336 |
|  | 1/2 MIC | 34.3425 | 37.7017 | 38.4420 | -1.138 | 0.399566 | 0.738439 |
|  | MIC | 30.3047 | 32.3068 | 28.0510 | 0.039395 | 0.97972 | -1.01912 |
| Sodium dehydroacetate | 1/4 MIC | 34.4925 | 32.2717 | 33.9942 | 0.77782 | -1.128 | 0.350175 |
|  | 1/2 MIC | 22.9090 | 24.7908 | 24.4954 | -1.14234 | 0.717114 | 0.425223 |
|  | MIC | 11.4983 | 10.8200 | 12.6908 | -0.18097 | -0.89716 | 1.078128 |
| Sodium benzoate | 1/4 MIC | 51.6548 | 52.0624 | 54.5835 | -0.70105 | -0.44408 | 1.145129 |
|  | 1/2 MIC | 30.7095 | 30.8626 | 32.6022 | -0.64866 | -0.50297 | 1.151633 |
|  | MIC | 13.9708 | 15.7103 | 15.2727 | -1.12043 | 0.802035 | 0.318395 |
| Composite organic acid salt | 1/4 MIC | 19.3803 | 19.6403 | 19.8678 | -1.02147 | 0.044414 | 0.977053 |
|  | 1/2 MIC | 13.9227 | 14.6961 | 15.2155 | -1.05873 | 0.130218 | 0.928512 |
|  | MIC | 5.5801 | 6.0442 | 5.9006 | -1.10068 | 0.852638 | 0.248037 |

## Ergosterol

| Group | | Ergosterol (%) | | | Z-score normalization | | |
| --- | --- | --- | --- | --- | --- | --- | --- |
| CK | 0 | 0.21761 | 0.228972 | 0.305428 | -0.6922 | -0.4543 | 1.146502 |
| Sodium diacetate | 1/4 MIC | 0.199024 | 0.263474 | 0.27294 | -1.14668 | 0.455678 | 0.691002 |
|  | 1/2 MIC | 0.178102 | 0.262153 | 0.26634 | -1.15368 | 0.534784 | 0.618895 |
|  | MIC | 0.174395 | 0.258883 | 0.260348 | -1.15457 | 0.562398 | 0.592174 |
| Sodium dehydroacetate | 1/4 MIC | 0.19848 | 0.259934 | 0.262603 | -1.15392 | 0.540174 | 0.613745 |
|  | 1/2 MIC | 0.185115 | 0.254007 | 0.257697 | -1.15352 | 0.531634 | 0.62189 |
|  | MIC | 0.179101 | 0.272094 | 0.269734 | -1.15441 | 0.599463 | 0.554952 |
| Sodium benzoate | 1/4 MIC | 0.212922 | 0.278301 | 0.279401 | -1.15458 | 0.562842 | 0.591738 |
|  | 1/2 MIC | 0.192438 | 0.249657 | 0.257274 | -1.14801 | 0.466547 | 0.681467 |
|  | MIC | 0.185368 | 0.258609 | 0.260653 | -1.15437 | 0.553361 | 0.601011 |
| Composite organic acid salt | 1/4 MIC | 0.196871 | 0.297758 | 0.303627 | -1.15332 | 0.527757 | 0.625562 |
|  | 1/2 MIC | 0.181097 | 0.266047 | 0.267867 | -1.15451 | 0.558898 | 0.595608 |
|  | MIC | 0.150998 | 0.248979 | 0.244348 | -1.15369 | 0.618724 | 0.534964 |

## Trehalose

| Group | | Trehalose (ug/mg) | | | Z-score normalization | | |
| --- | --- | --- | --- | --- | --- | --- | --- |
| CK | 0 | 46.28934 | 46.05477 | 45.92395 | 1.080231 | -0.18681 | -0.89342 |
| Sodium diacetate | 1/4 MIC | 35.31785 | 33.82719 | 35.04655 | 0.739706 | -1.13772 | 0.398018 |
|  | 1/2 MIC | 21.20711 | 20.94067 | 20.6966 | 1.014282 | -0.0292 | -0.98508 |
|  | MIC | 19.60971 | 19.26266 | 19.11083 | 1.102646 | -0.25446 | -0.84819 |
| Sodium dehydroacetate | 1/4 MIC | 23.16642 | 22.70068 | 22.48023 | 1.09606 | -0.2334 | -0.86266 |
|  | 1/2 MIC | 20.41127 | 21.1443 | 20.7807 | -1.00264 | 0.997344 | 0.005292 |
|  | MIC | 16.60711 | 16.29947 | 16.01489 | 1.012731 | -0.02597 | -0.98676 |
| Sodium benzoate | 1/4 MIC | 28.93414 | 29.5592 | 29.28278 | -1.03621 | 0.959355 | 0.076854 |
|  | 1/2 MIC | 22.47943 | 22.43461 | 22.13827 | 0.694007 | 0.452225 | -1.14623 |
|  | MIC | 16.44711 | 16.57323 | 17.38102 | -0.69731 | -0.44841 | 1.145724 |
| Composite organic acid salt | 1/4 MIC | 16.59312 | 16.44334 | 16.18123 | 0.898027 | 0.179605 | -1.07763 |
|  | 1/2 MIC | 14.30018 | 14.49161 | 14.21784 | -0.2589 | 1.10399 | -0.84509 |
|  | MIC | 14.33452 | 14.08602 | 13.71597 | 0.928542 | 0.130168 | -1.05871 |

## SOD

| Group | | SOD (U/g FW) | | | Z-score normalization | | |
| --- | --- | --- | --- | --- | --- | --- | --- |
| CK | 0 | 87.8742 | 89.15474 | 86.27352 | 0.07392 | 0.960989 | -1.03491 |
| Sodium diacetate | 1/4 MIC | 102.4502 | 101.9043 | 101.5524 | 1.063798 | -0.14299 | -0.92081 |
|  | 1/2 MIC | 102.2457 | 109.0024 | 106.9131 | -1.1008 | 0.852366 | 0.248431 |
|  | MIC | 114.7367 | 110.0283 | 111.4042 | 1.107103 | -0.8377 | -0.2694 |
| Sodium dehydroacetate | 1/4 MIC | 102.7971 | 108.151 | 106.2148 | -1.07856 | 0.896395 | 0.182165 |
|  | 1/2 MIC | 106.8036 | 107.9009 | 106.9967 | -0.7343 | 1.138902 | -0.4046 |
|  | MIC | 115.1789 | 116.8052 | 116.5555 | -1.1429 | 0.714047 | 0.428853 |
| Sodium benzoate | 1/4 MIC | 110.5693 | 111.1001 | 110.2019 | -0.12068 | 1.054865 | -0.93418 |
|  | 1/2 MIC | 118.393 | 117.1362 | 113.8647 | 0.824931 | 0.28726 | -1.11219 |
|  | MIC | 119.8723 | 121.0751 | 117.6236 | 0.199018 | 0.885526 | -1.08454 |
| Composite organic acid salt | 1/4 MIC | 109.4776 | 107.1953 | 104.0573 | 0.943463 | 0.104817 | -1.04828 |
|  | 1/2 MIC | 113.0804 | 116.6178 | 109.7783 | -0.02293 | 1.011269 | -0.98834 |
|  | MIC | 117.6509 | 126.4332 | 122.2364 | -1.01442 | 0.984923 | 0.029501 |

## CAT

| Group | | CAT (umol/min/g FW) | | | Z-score normalization | | |
| --- | --- | --- | --- | --- | --- | --- | --- |
| CK | 0 | 1407.869 | 1370.820 | 1383.169 | 1.091089 | -0.87287 | -0.21822 |
| Sodium diacetate | 1/4 MIC | 1209.577 | 1320.986 | 1305.070 | -1.14459 | 0.704361 | 0.440225 |
|  | 1/2 MIC | 833.422 | 833.422 | 810.897 | 0.57735 | 0.57735 | -1.1547 |
|  | MIC | 757.765 | 757.765 | 797.647 | -0.57735 | -0.57735 | 1.154701 |
| Sodium dehydroacetate | 1/4 MIC | 834.258 | 953.438 | 860.742 | -0.77588 | 1.128553 | -0.35267 |
|  | 1/2 MIC | 809.552 | 910.746 | 832.040 | -0.77588 | 1.128553 | -0.35267 |
|  | MIC | 727.192 | 727.192 | 748.580 | -0.57735 | -0.57735 | 1.154701 |
| Sodium benzoate | 1/4 MIC | 1376.201 | 1388.827 | 1350.950 | 0.218218 | 0.872872 | -1.09109 |
|  | 1/2 MIC | 1134.612 | 1148.449 | 1106.939 | 0.218218 | 0.872872 | -1.09109 |
|  | MIC | 760.090 | 760.090 | 820.897 | -0.57735 | -0.57735 | 1.154701 |
| Composite organic acid salt | 1/4 MIC | 1079.534 | 1237.515 | 1000.544 | -0.21822 | 1.091089 | -0.87287 |
|  | 1/2 MIC | 954.466 | 938.010 | 938.010 | 1.154701 | -0.57735 | -0.57735 |
|  | MIC | 671.892 | 732.973 | 671.892 | -0.57735 | 1.154701 | -0.57735 |

## GSH-Px

| Group | | GSH-Px (nmol/min/g FW) | | | Z-score normalization | | |
| --- | --- | --- | --- | --- | --- | --- | --- |
| CK | 0 | 224.812 | 177.483 | 224.812 | 0.57735 | -1.1547 | 0.57735 |
| Sodium diacetate | 1/4 MIC | 417.662 | 338.108 | 357.996 | 1.120897 | -0.80064 | -0.32026 |
|  | 1/2 MIC | 330.411 | 489.498 | 391.598 | -0.91499 | 1.06749 | -0.1525 |
|  | MIC | 573.541 | 688.249 | 615.253 | -0.89803 | 1.077632 | -0.17961 |
| Sodium dehydroacetate | 1/4 MIC | 279.923 | 248.820 | 290.290 | 0.320256 | -1.1209 | 0.800641 |
|  | 1/2 MIC | 283.598 | 255.238 | 311.958 | 2E-15 | -1 | 1 |
|  | MIC | 312.315 | 320.756 | 261.669 | 0.440225 | 0.704361 | -1.14459 |
| Sodium benzoate | 1/4 MIC | 387.470 | 322.892 | 339.036 | 1.120897 | -0.80064 | -0.32026 |
|  | 1/2 MIC | 388.189 | 432.981 | 403.120 | -0.87287 | 1.091089 | -0.21822 |
|  | MIC | 580.805 | 597.399 | 613.994 | -1 | 6.85E-15 | 1 |
| Composite organic acid salt | 1/4 MIC | 273.869 | 224.964 | 254.307 | 0.927173 | -1.05963 | 0.132453 |
|  | 1/2 MIC | 335.000 | 345.469 | 324.531 | 5.43E-15 | 1 | -1 |
|  | MIC | 722.697 | 752.809 | 722.697 | -0.57735 | 1.154701 | -0.57735 |

## MDA

| Group | | MDA (nmol/g) | | | Z-score normalization | | |
| --- | --- | --- | --- | --- | --- | --- | --- |
| CK | 0 | 5.771 | 6.899 | 5.307 | -0.27009 | 1.107304 | -0.83722 |
| Sodium diacetate | 1/4 MIC | 12.242 | 10.838 | 11.150 | 1.128553 | -0.77588 | -0.35267 |
|  | 1/2 MIC | 10.994 | 12.008 | 12.164 | -1.14596 | 0.450199 | 0.695764 |
|  | MIC | 13.969 | 14.046 | 13.511 | 0.440228 | 0.704359 | -1.14459 |
| Sodium dehydroacetate | 1/4 MIC | 9.603 | 10.155 | 9.741 | -0.80064 | 1.120897 | -0.32026 |
|  | 1/2 MIC | 8.702 | 13.684 | 10.947 | -0.96557 | 1.031195 | -0.06562 |
|  | MIC | 15.019 | 12.607 | 12.140 | 1.141447 | -0.41965 | -0.7218 |
| Sodium benzoate | 1/4 MIC | 8.583 | 10.866 | 8.346 | -0.49039 | 1.150534 | -0.66014 |
|  | 1/2 MIC | 10.858 | 13.105 | 8.674 | -0.00939 | 1.004662 | -0.99527 |
|  | MIC | 14.989 | 13.517 | 12.966 | 1.113799 | -0.2931 | -0.8207 |
| Composite organic acid salt | 1/4 MIC | 9.573 | 12.512 | 11.469 | -1.08168 | 0.890799 | 0.190885 |
|  | 1/2 MIC | 13.633 | 12.327 | 10.449 | 0.935162 | 0.119023 | -1.05418 |
|  | MIC | 15.612 | 16.621 | 11.961 | 0.35905 | 0.770902 | -1.12995 |

## qPCR

| Group | | Relative expression level | | | Z-score normalization | | |
| --- | --- | --- | --- | --- | --- | --- | --- |
| *aflR* | CK | 1.009878 | 0.906575 | 1.092264 | 0.074942 | -1.03536 | 0.960421 |
|  | MIC | 0.129371 | 0.126477 | 0.129617 | 0.505464 | -1.15183 | 0.646367 |
|  | 1/2 MIC | 0.820551 | 0.945928 | 0.973427 | -1.13815 | 0.400356 | 0.737791 |
| *aflS* | CK | 1.048461 | 0.870188 | 1.096061 | 0.365818 | -1.1314 | 0.765581 |
|  | MIC | 0.103174 | 0.084271 | 0.126764 | -0.07337 | -0.96129 | 1.034665 |
|  | 1/2 MIC | 0.767279 | 0.863773 | 0.889436 | -1.13156 | 0.366563 | 0.764992 |
| *aflD* | CK | 0.957936 | 0.884035 | 1.180847 | -0.32146 | -0.79974 | 1.121197 |
|  | MIC | 0.177152 | 0.137046 | 0.217495 | -0.00197 | -0.99901 | 1.000983 |
|  | 1/2 MIC | 1.113226 | 1.449963 | 1.238332 | -0.90445 | 1.073897 | -0.16944 |
| *aflM* | CK | 1.203855 | 0.818648 | 1.014679 | 0.994014 | -1.00588 | 0.011866 |
|  | MIC | 0.160663 | 0.07371 | 0.198322 | 0.257104 | -1.10345 | 0.846344 |
|  | 1/2 MIC | 1.145639 | 1.21286 | 1.315758 | -0.92335 | -0.1388 | 1.062149 |
| *aflC* | CK | 0.933382 | 0.992728 | 1.079221 | -0.93258 | -0.12339 | 1.055968 |
|  | MIC | 0.122105 | 0.117706 | 0.111212 | 0.930157 | 0.127463 | -1.05762 |
|  | 1/2 MIC | 0.563985 | 0.851011 | 0.764759 | -1.10408 | 0.844875 | 0.259208 |
| *aflT* | CK | 1.149332 | 0.927587 | 0.937994 | 1.153702 | -0.61844 | -0.53527 |
|  | MIC | 0.118617 | 0.114716 | 0.112451 | 1.075874 | -0.1748 | -0.90108 |
|  | 1/2 MIC | 0.769147 | 0.765956 | 0.715184 | 0.629253 | 0.523843 | -1.1531 |
